# Supplementary material for: Consensus-building conversation leads to neural alignment
Source: Nat Commun. 2024 May 10;15:3936. doi: 10.1038/s41467-023-43253-8 (PMC11087652; doi:10.1038/s41467-023-43253-8)
Supplement: Supplementary file 1 — Supplementary Information [file 41467_2023_43253_MOESM1_ESM.pdf]

# Consensus-building conversation leads to neural alignment

# Supplementary information

Beau Sievers[1,2], Christopher Welker[1], Uri Hasson[3], Adam M. Kleinbaum[4], Thalia Wheatley[1,5]

[1]Department of Psychological and Brain Sciences, Dartmouth College, Hanover, NH 03755

[2]Department of Psychology, Stanford University, Stanford, CA 94305

[3]Princeton Neuroscience Institute and Department of Psychology, Princeton University, Princeton, NJ 08544

[4]Tuck School of Business, Dartmouth College, Hanover, NH 03755

[5]Santa Fe Institute, Santa Fe, NM 87501

Correspondence to: Beau Sievers, 450 Jane Stanford Way, Stanford University, Stanford, CA 94305. beau@beausievers.com

## Supplementary discussion

### Comparing behavioral and neural results

To confirm that the reported results were related to shifts in participants' beliefs about the movie clips, we tested whether neural alignment and influence were correlated with behavioral alignment and influence. For the change analysis, we assessed the Pearson correlation between change in participants' meaning vectors (yes-or-no survey answers) and the summed positive and negative mass of the unthresholded pairwise change maps. This analysis was conducted over all unordered pairs of participants where meaning vector change could be calculated across all five runs. Meaning vector change was correlated with positive ($R(3476)=0.09$, two-tailed permutation $p<.001$, $n=3478$ unordered participant pairs)—but not negative ($R(3476)=0.02$, two-tailed permutation $p=0.286$, $n=3478$ unordered participant pairs)—change map mass, indicating that change in belief predicted neural change, regardless of group membership (see manuscript Figure 1). For the neural influence analysis, we assessed the Pearson correlation between influence assessed over the yes-or-no survey answers and the summed positive or negative mass of the unthresholded pairwise neural influence maps. This analysis was conducted over all ordered pairs of participants where influence could be calculated across all five runs. Survey answer influence was correlated with positive mass ($R(8618)=0.06$, two-tailed permutation $p<.001$, $n=8620$ ordered participant pairs) but not negative mass ($R(8618)=0.02$, two-tailed permutation $p=0.092$, $n=8620$ unordered participant pairs), indicating a significant relationship between belief and neural influence, regardless of group membership (Supplementary Figure 13). P-values were calculated using permutation testing to account for network autocorrelation. Because the number of yes-or-no questions about each clip was limited, and these questions did not necessarily reflect what participants found salient, this analysis may understate the magnitude of neural alignment and influence that could be predicted from behavioral alignment and influence.

### What did participants discuss in conversation?

Participants drew on a rich repertoire of approaches to making and sharing inferences about the movie clips, carefully considering both the diegetic (within-narrative) content of the movies and the social context of their production (e.g., when the movies were made, the intentions of their makers, and so on). See https://osf.io/kr9fb/ for complete conversation transcripts. Below, we qualitatively describe a subset of participants' aproaches to conversation and provide transcript excerpts illustrating their use.

Participants discussed the content of the movies in terms of how actions taken by the characters revealed information about their attitudes and social roles. For example, in the excerpt below, the participants discuss whether the actions of a woman toward a child suggest whether the woman is the child's mother. Speakers 4 and 5 suggest that the woman's way of interacting with the child was detached and apathetic, making it unlikely that the woman was the child's mother.

> Group I, Movie Clip 1
>
> 04:26 Speaker 5: She didn't seem though that like…
>
> 04:28 Speaker 4: Attached to him.
>
> 04:29 Speaker 5: Attached, like positive or negative.
>
> 04:32 Speaker 4: Yeah.
>
> 04:32 Speaker 5: You know what I mean? She seemed a little like apathetic.
>
> 04:38 Speaker 3: She has like this Cruella-ish strange type of feeling with the child, it almost I don't know… It really just seemed like it was like…
>
> 04:45 Speaker 4: It doesn't have the motherly affection.
>
> 04:48 Speaker 3: Like zero, I didn't get any of that vibe, it literally felt like it was like some estranged sister's son or I don't know…

Participants discussed the content of the movies in terms of the social roles of the characters and the historical context of those roles. For example, in the excerpt below, the participants discuss whether it is reasonable to infer that a character with a gun is an "outlaw." Speaker 4 notes that the character appears to have a conventional nuclear family, implying this makes it less likely that the character is an outlaw. Speaker 5 then notes that in the old Western setting of the film, it might be reasonable for anyone to have a gun, outlaw or not. Finally, Speaker 3 suggests that even if anyone might have a gun, only criminals would be likely to keep their gun nearby at all times.

> Group H, Movie Clip 2
>
> 18:33 Speaker 4: Those times everyone was using a gun, right?
>
> 18:37 Speaker 2: Yeah, it also seemed like they're out west, like western cowboy vibe.
>
> 18:42 Speaker 4: But would you be an outlaw with that family?
>
> 18:45 Speaker 5: No, you could have a gun. Anyone.

> 18:50 Speaker 3: Even so, if you weren’t a criminal, are you gonna keep it on your side?
>
> 18:55 Speaker 4: Yeah. Fair point.

Participants discussed how technical decisions made by the filmmakers provide information about the narrative. In the excerpt below, the participants discuss whether two characters are married to each other. Speaker 4 asserts that they were in a relationship, but not married, and claims that if the director wanted the audience to believe the characters had a deep connection, then the characters would have been shown on the screen at the same time more than they were.

> Group A, Movie Clip 1
>
> 13:55 Speaker 4: Yeah. I didn’t think they were married. I thought they were in a relationship.
>
> 14:00 Speaker 2: I agree.
>
> 14:00 Speaker 3: It’s the same thing though, right?
>
> 14:01 Speaker 4: No, ’cause I feel like they would have been in the frame together more if they were married.
>
> 14:06 Speaker 2: I also didn’t remember seeing any rings.
>
> 14:08 Speaker 4: Yeah, I haven’t see any… He’s not… He doesn’t have any ring on.
>
> 14:11 Speaker 2: It’s on his right hand, though.
>
> 14:12 Speaker 4: Oh, yeah, that’s true. And, well, I feel like at least from what I remember, they weren’t really in the same shot together, the same frame.
>
> 14:20 Speaker 3: Why does that matter?
>
> 14:22 Speaker 4: ’Cause directors are really visual and they would try to show that they’re connected in a deep way.
>
> 14:29 Speaker 3: Okay.

## Supplementary methods

### Survey questions

#### All movies

The following free response questions were asked for each movie clip in each session.

- What happened during the clip you just watched? Explain who each of the characters are, what their relationships are, and why they did what they did.
- What do you think will happen in the next scene of the movie?
- How do you think this movie will end?

### Birth

fMRI session 1, group session:

- Is the man wearing the tuxedo the child's father?
- Are the man in the tuxedo and the woman in the beige dress married?
- Is the child related to the older couple who answer the door?
- Has the child been abandoned?
- Will this movie have a happy ending?

fMRI session 2:

- Does the child know why the woman in the jacket has dirty hands?
- Is the woman with short hair the child's mother?
- Are the three women discussing the child?
- Is the child in any serious danger?
- Will this movie have a happy ending?

### The Assassination of Jesse James by the Coward Robert Ford

fMRI session 1, group session:

- Is the man taking a bath related to the man standing in the doorframe?
- Is the man in the doorframe sexually attracted to the man in the bath?
- Are the man in the bath and the man in the doorframe business partners?
- Is the man in the bath a criminal?
- Will this movie have a happy ending?

fMRI session 2:

- Has the clean shaven man been romantically involved with the woman?
- Are the clean shaven man and the bearded man brothers?
- Did the clean shaven man steal from the bearded man?
- Was the bearded man playing a prank on the clean shaven man?
- Will this movie have a happy ending?

### Sexy Beast

fMRI session 1, group session:

- Are the bald man and the blonde man in the blue shirt close friends?
- Has the bald man been asked to help the other characters?
- Is the bald man angry at the blonde man in the blue shirt?
- Did the blonde man in the blue shirt commit a crime?
- Will this movie have a happy ending?

fMRI session 2:

- Did the bald man cause the blonde man’s injuries?
- Are the bald man and the blonde man arguing about the woman?
- Is the blonde man trying to protect the woman?
- Is the bald man angry because of something the blonde man did to him?
- Will this movie have a happy ending?

**The Master**

fMRI session 1, group session:

- Are the blonde man with the mustache and the woman on the couch related?
- Are the characters discussing politics?
- Does the dark haired man standing in the doorframe make a convincing argument?
- Are the young woman and the blonde man related?
- Will this movie have a happy ending?

fMRI session 2:

- Did the men on the porch commit a crime?
- Are the people standing behind the blonde man with the mustache his family members?
- Are the police justified in their arrest of the blonde man with the mustache?
- Is the man who struggles with the police trying to attack the blonde man?
- Will this movie have a happy ending?

**Y tu mamá también**

fMRI session 1, group session:

- Are the woman and the young man sitting by the tree related?
- Are the woman and the young man driving the car romantically involved?
- Are the two young men related?
- Did the actions of the young man sitting by the tree cause the argument?
- Will this movie have a happy ending?

fMRI session 2:

- Was the meeting of the characters on the beach planned in advance?
- Is everyone in this clip part of one family?
- Is the woman in the black bathing suit wealthy?
- Did the pigs destroy something important to one of the characters?
- Will this movie have a happy ending?

## Supplementary Figures

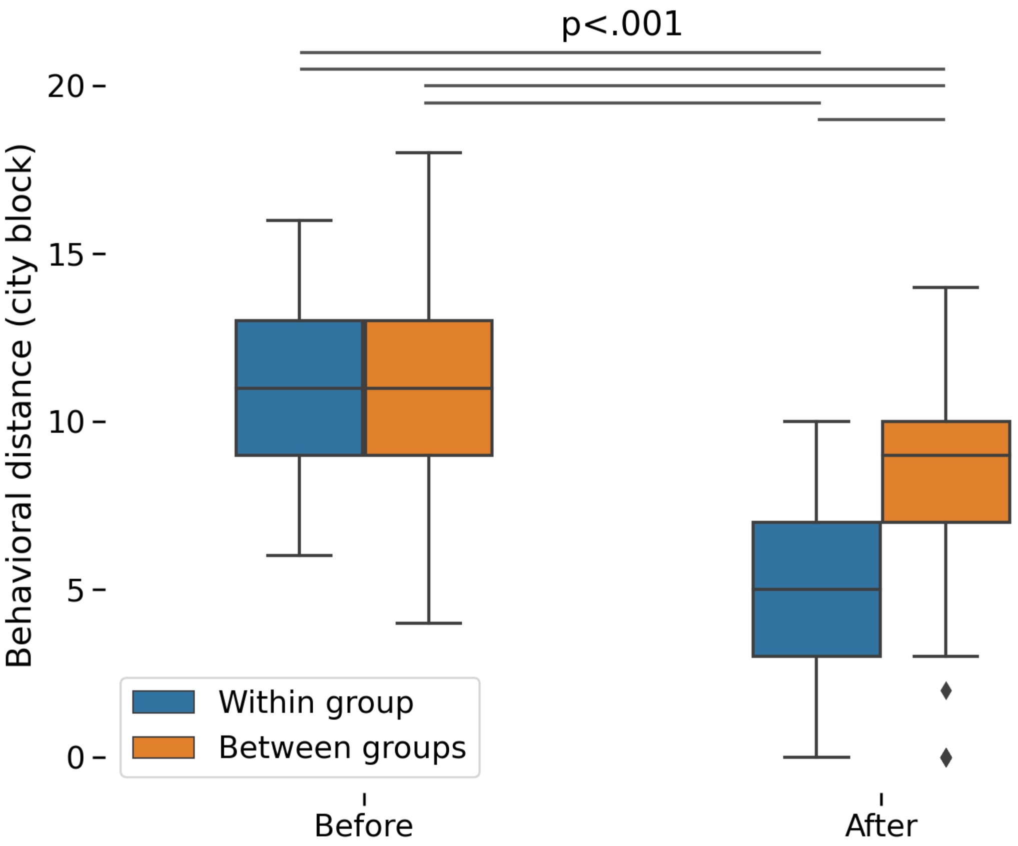


**Supplementary Figure 1:** Conversation-related change in consensus. After conversation, participants' survey answers became more similar to members of their conversation group. Box bounds show dataset quartiles, with the center line showing the median. Whiskers extend to points that lie within 1.5 times the range of the lower and upper quartiles, and diamonds show data points outside of this range. Hierarchical linear regresison was used to test the effect of session (before versus after) and comparison type (within versus between groups) on behavioral distance. The model included both predictors and their interaction, as well as random intercepts for participant pairs. The model significantly explained variance in behavioral distance (marginal $R^2$=0.28, p<.001, n=1369 participant pairs). Session, comparison type, and their interaction all significantly predicted behavioral distance. Distance was higher before conversation ({$\beta$}=2.78, 95% CI=[2.48, 3.08], p<.001), lower within groups ({$\beta$}=-3.67, 95% CI=[-4.4, -2.94], p<.001), and higher before conversation within group ({$\beta$}=3.67, 95% CI=[2.67, 4.67], p<.001). Horizontal lines at top denote t-tests significant at two-tailed p<.001. For all analyses in this figure, total n=1271 after-conversation participant pairs, including 1210 between-group pairs and 61 within-group pairs, excluding the control group.

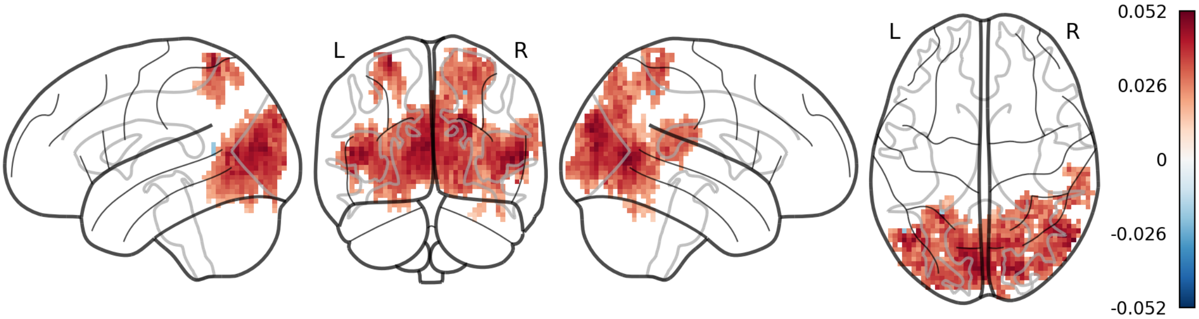


**Supplementary Figure 2:** Convergent change across conversation groups. Brain map shows the multiple regression beta weight for being in any conversation group across all movie clips, capturing between-group convergence of change in ISC (n=703 participant pairs, cluster forming threshold=.05, minimum cluster size=115 voxels, two-tailed permutation p corrected=.05) (see *Methods: Conversation-induced change in ISC*). This figure shows change that was shared across groups, and does not show group- and movie-specific change; see Supplementary Figure 4. Change was measured as before-conversation ISC subtracted from after-conversation ISC, and reflects changes in group neural alignment caused by conversation. Brain maps created using the Python package `nilearn`.

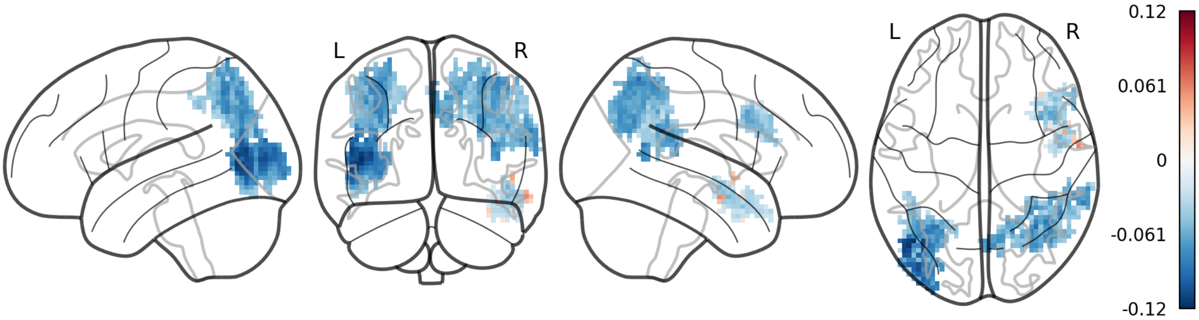


**Supplementary Figure 3:** Control group. Brain map shows the multiple regression beta weight for being in the control group across all movie clips (n=703 participant pairs, cluster forming threshold=.05, minimum cluster size=115 voxels, two-tailed permutation p corrected=.05) (see *Methods: Conversation-induced change in ISC*). For comparison with Supplementary Figure 2. Brain maps created using the Python package `nilearn`.

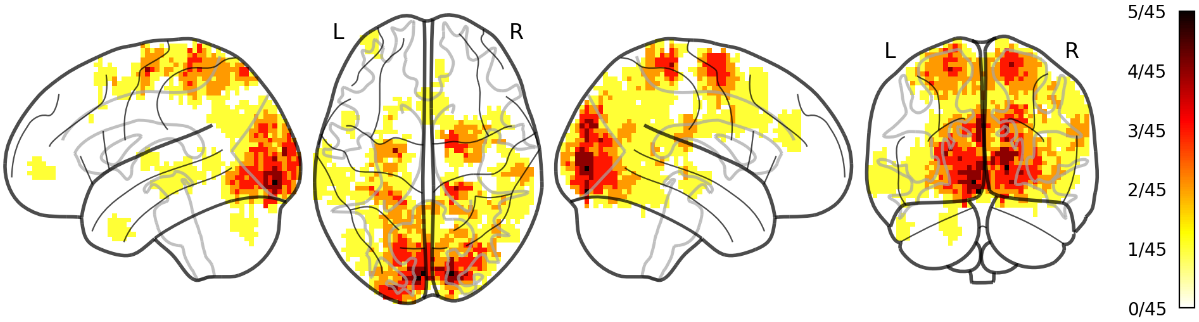


**Supplementary Figure 4:** Conjunction map showing group- and movie-specific change. Separate multiple regression models were fit for each movie clip. Brain map shows the number of clusters at each voxel that surivived multiple comparisons correction at the group–movie combination level (n=703 participant pairs, cluster forming threshold=.01, minimum cluster size=32 voxels, two-tailed permutation p corrected=.05) (see *Methods: Conversation-induced change in ISC*). The maximum possible number of overlapping clusters was 45, and the maximum observed number of overlapping clusters was 5 (or 11%). Change was measured as before-conversation ISC subtracted from after-conversation ISC, and reflects changes in group neural alignment caused by conversation. Brain maps created using the Python package `nilearn`.

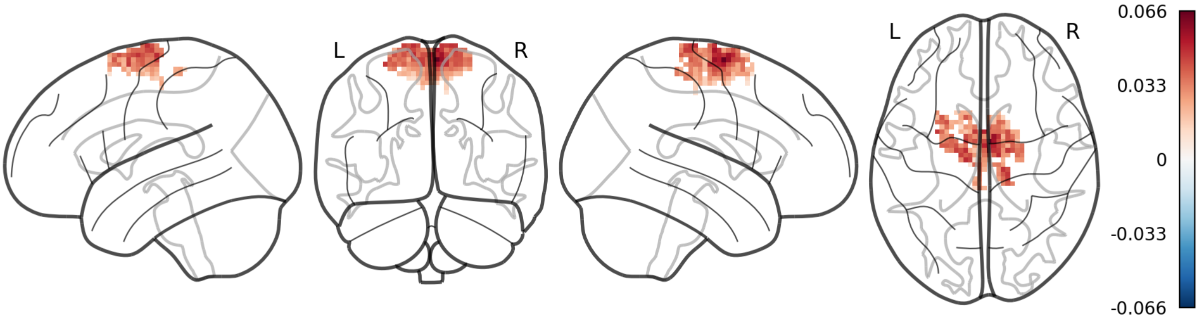


**Supplementary Figure 5:** Convergent alignment for novel movie clips across conversation groups. A regression model was used to predict ISC across all novel movie clips viewed only during the second fMRI session. Brain map shows the beta weight for being in any conversation group (n=703 participant pairs, cluster forming threshold=.05, minimum cluster size=115 voxels, two-tailed permutation p corrected=.05) (see *Methods: Conversation-induced change in ISC*). This figure shows alignment that was shared across groups, and does not show group- and movie-specific alignment; see Supplementary Figure 6. Brain maps created using the Python package `nilearn`.

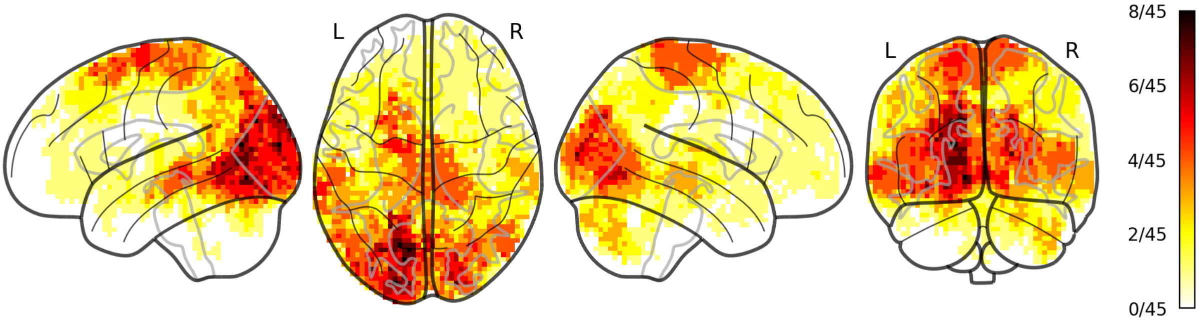


**Supplementary Figure 6:** Conjunction map showing group- and movie-specific alignment for novel movie clips. Separate regression models were fit for each movie clip, with predictors for each group. Brain map shows the number of clusters at each voxel that survived multiple comparisons correction at the group–movie combination level (n=703 participant pairs, cluster forming threshold=.01, minimum cluster size=32 voxels, two-tailed permutation p corrected=.05) (see *Methods: Conversation-induced change in ISC*). The maximum possible number of overlapping clusters was 45, and the maximum observed number of overlapping clusters was 8 (or 18%). Brain maps created using the Python package `nilearn`.

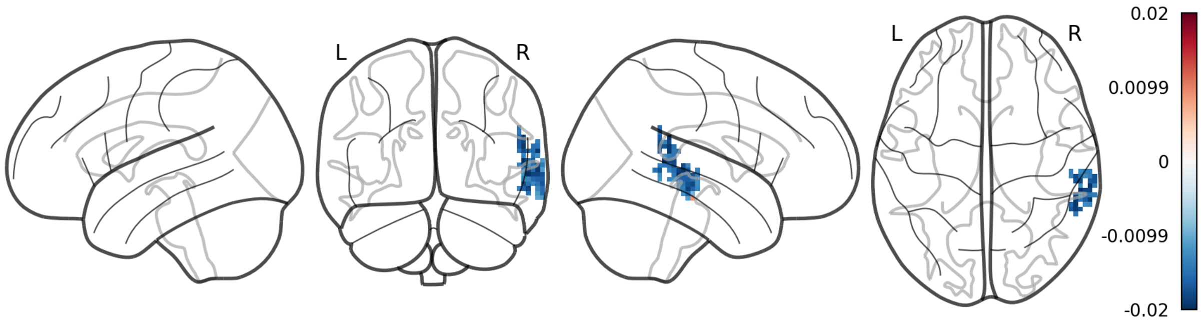


**Supplementary Figure 7:** Convergent neural influence, ego PCA centrality. Brain map shows the multiple regression beta weight for ego PCA centrality on neural influence across all conversation groups and movie clips (n=1,406 ordered participant pairs, cluster forming threshold=.05, minimum cluster size=115 voxels, two-tailed permutation p corrected=.05). This figure does not show group- and movie-specific neural influence; see Supplementary Figures 11 and 12. For each possible pair of participants, one was designated the ego while the other was designated the alter. Neural influence was measured by subtracting the pair's before-conversation ISC from the correlation of the ego's before-conversation timeseries and the alter's after-conversation timeseries, capturing the movement of the alter toward the ego's initial position (see *Methods: Neural influence*). Brain maps created using the Python package `nilearn`.

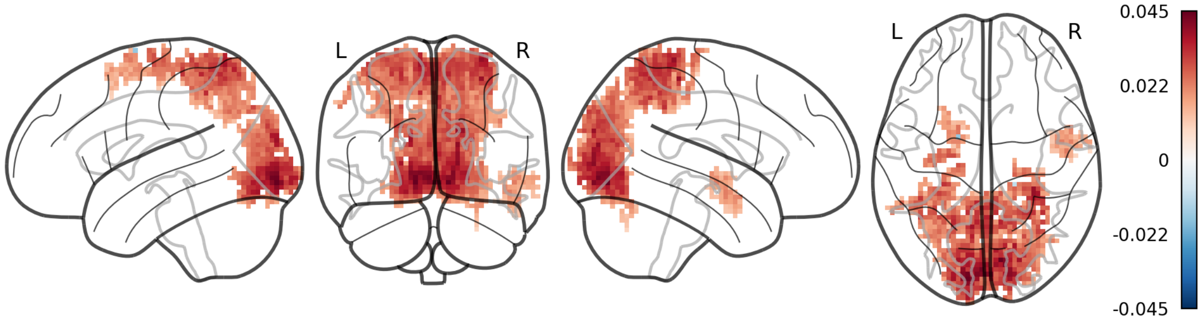


**Supplementary Figure 8:** Convergent neural influence, alter PCA centrality. Brain map shows the multiple regression beta weight for alter PCA centrality on neural influence, across all conversation groups and movie clips (n=1,406 ordered participant pairs, cluster forming threshold=.05, minimum cluster size=115 voxels, two-tailed permutation p corrected=.05) (see *Methods: Neural influence*). Brain maps created using the Python package `nilearn`.

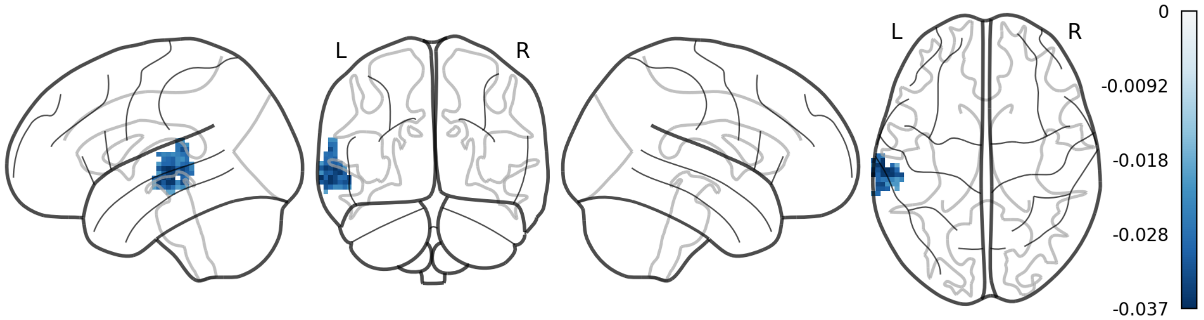


**Supplementary Figure 9:** Convergent neural influence, ego eigenvector centrality. Brain map shows beta weight for ego eigenvector centrality on neural influence, across all conversation groups and movie clips (n=1,406 ordered participant pairs, cluster forming threshold=.05, minimum cluster size=115 voxels, two-tailed permutation p corrected=.05) (see *Methods: Neural influence*).

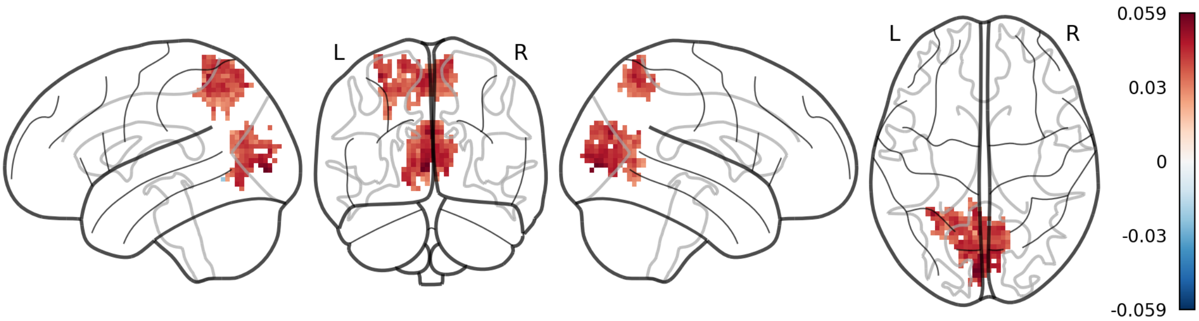


**Supplementary Figure 10:** Convergent neural influence, alter eigenvector centrality. Brain map shows multiple regression beta weight for alter eigenvector centrality on neural influence, across all conversation groups and movie clips (n=1,406 ordered participant pairs, cluster forming threshold=.05, minimum cluster size=115 voxels, two-tailed permutation p corrected=.05). Brain maps created using the Python package `nilearn`.

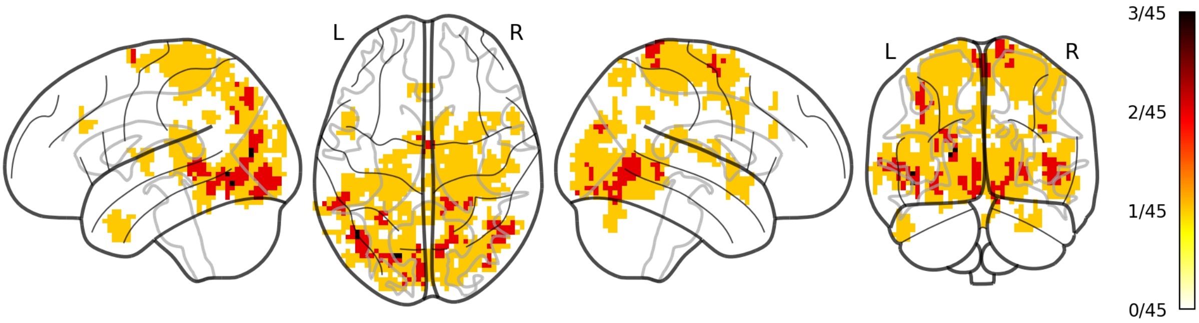


**Supplementary Figure 11:** Group- and movie-specific neural influence, ego PCA centrality. Brain map shows the number of movie–group combinations with significant PCA centrality-dependent neural influence values for the ego (n=1,406 ordered participant pairs, cluster forming threshold=.01, minimum cluster size=32 voxels, two-tailed permutation p corrected=.05) (see *Methods: Neural influence*). The maximum possible number of overlapping clusters was 45, and the maximum observed number of overlapping clusters was 3 (or 7%). Brain maps created using the Python package `nilearn`.

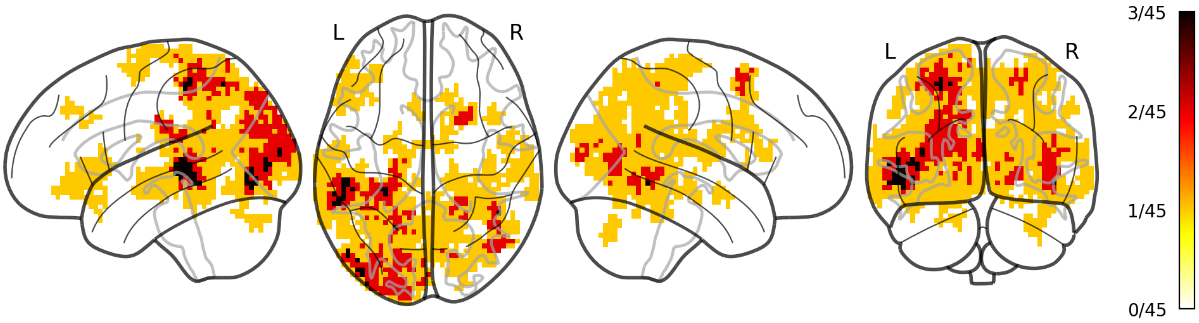


**Supplementary Figure 12:** Group- and movie-specific neural influence, alter PCA centrality. Brain map shows the number of movie–group combinations with significant PCA centrality-dependent neural influence values for the ego (n=1,406 ordered participant pairs, cluster forming threshold=.01, minimum cluster size=32 voxels, two-tailed permutation p corrected=.05) (see *Methods: Neural influence*). The maximum possible number of overlapping clusters was 45, and the maximum observed number of overlapping clusters was 3 (or 7%). Brain maps created using the Python package `nilearn`.

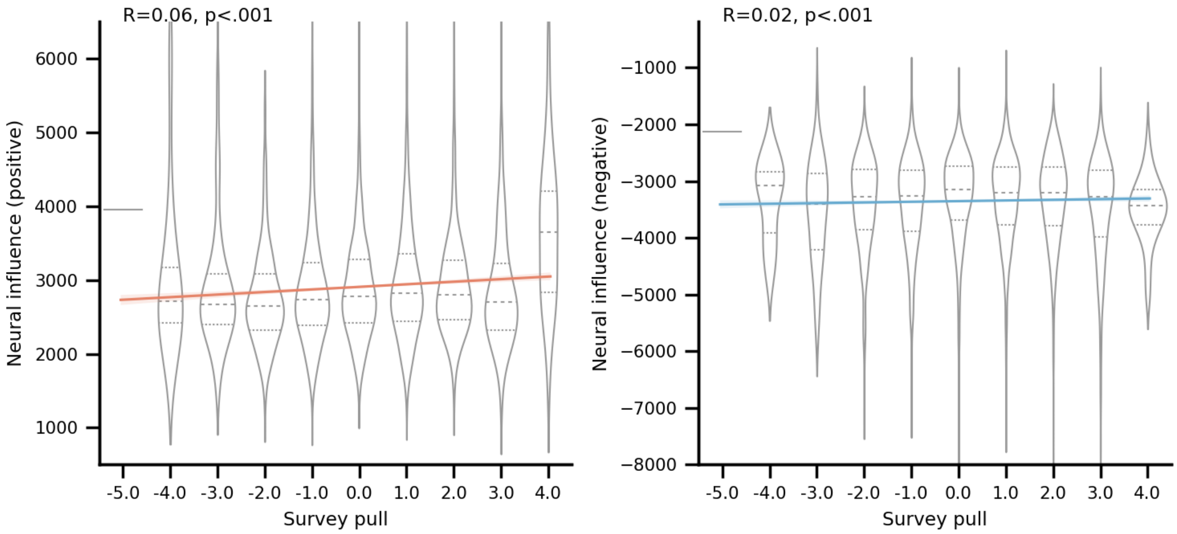


**Supplementary Figure 13:** Survey questions and neural influence. Correlation of pairwise influence over participants' answers to the yes-or-no survey questions with unthresholded neural influence (n=8,720 ordered participant pairs, over all five movie clips). The central diagonal line and shaded region shows the regression line of best fit and its 95% confidence interval. Violin plots use width to represent the density of the distribution at each x-axis value, with a central dashed line showing the median and dotted lines showing the lower and upper quartiles. The single solid horizontal line at x=-5 represents a single data point.

# Supplementary Tables

**Supplementary Table 1:** Regression results for content analysis at the group–movie level (n=45 group–movie combinations). `n_words` is the number of words spoken by the group; `speak_gi` is the Gini coefficient of words spoken; `cent_gi` is the Gini coefficient of PCA centrality; `status_gi` is the Gini coefficient of coder status judgments. The neural alignment DV was the positive mass of the change map, and the behavioral alignment DV was the change in survey responses. Separate hierarchical models were fit for each alignment DV, with random intercepts for movie clips. Both regression models significantly explained variance in outcomes (neural alignment: marginal $R^2$=0.53, two-tailed permutation p<.001, n=45 group–movie pairs; behavioral alignment: marginal $R^2$=0.34, two-tailed permutation p<.001, n=45 group–movie pairs).

| Predictor | perm. p | $\beta$ | [2.5% | 97.5%] | Alignment DV |
|---|---|---|---|---|---|
| (Intercept) | <.001 | 0.438 | 0.235 | 0.641 | Neural |
| cent_gi | <.001 | 0.516 | 0.298 | 0.734 | Neural |
| n_words | <.001 | 0.56 | 0.327 | 0.793 | Neural |
| speak_gi | 0.037 | -0.35 | -0.565 | -0.134 | Neural |
| cent_gi:n_words | 0.04 | 0.321 | 0.108 | 0.533 | Neural |
| (Intercept) | <.001 | 2.134 | -0.153 | 4.421 | Behavioral |
| cent_gi | <.001 | 7.239 | 4.149 | 10.33 | Behavioral |
| status_gi | 0.118 | -5.459 | -11.089 | 0.171 | Behavioral |

| Predictor | perm. p | $\beta$ | [2.5% | 97.5%] | Alignment DV |
|---|---|---|---|---|---|
| cent_gi:status_gi | <.001 | -17.224 | -24.766 | -9.681 | Behavioral |

**Supplementary Table 2:** Regression results showing word stems predicting speaker centrality and status at the speech turn level. Separate regressions were conducted for centrality and status. Word use in each speech turn was modestly predictive of participants' PCA centrality (F(350, 7771)=2.0, two-tailed permutation p<.001, $R^2_{adj}$=0.04) and perceived status (F(350, 7771)=2.39, two-tailed permutation p<.001, $R^2_{adj}$=0.06). To exclude speaker– and movie–specific words, word stems were included only if used by 10 or more speakers across discussions of 3 or more movie clips. RAREWORD is a placeholder for all words used less than 5 times across all conversations. For concision, only words with two-tailed permutation P values < .05 for at least one analysis are shown. See Supplementary Data 2 for a complete regression table. See Methods for text preprocessing and regression details.

| Word stem | $\beta$ cent. | p cent. | $\beta$ status | p status |
|---|---|---|---|---|
| cool | 0.86 | <.001 | 0.32 | 0.002 |
| see | 0.86 | 0.0008 | | |
| gotta | 0.84 | 0.0032 | 0.36 | 0.0132 |
| resolv | 0.83 | 0.0004 | 0.6 | <.001 |
| for | 0.82 | 0.0058 | 0.36 | 0.0124 |
| dude | 0.74 | <.001 | 0.22 | 0.0108 |
| expect | 0.7 | 0.0216 | | |
| sexual | 0.7 | 0.0064 | | |
| then | 0.64 | 0.0066 | 0.26 | 0.024 |
| far | 0.59 | 0.0144 | | |
| shit | 0.53 | 0.0292 | 0.37 | 0.0012 |
| write | 0.52 | 0.0094 | 0.31 | 0.0006 |
| younger | 0.51 | 0.0122 | | |
| clearli | 0.47 | 0.0156 | 0.32 | 0.0016 |
| interest | 0.46 | 0.0304 | 0.21 | 0.0362 |
| fuck | 0.46 | 0.0222 | | |
| type | 0.44 | 0.0068 | 0.17 | 0.0432 |
| mom | 0.44 | 0.019 | | |
| deal | 0.43 | 0.0152 | | |
| weird | 0.41 | 0.0084 | | |
| charact | 0.4 | 0.0014 | | |
| wasn | 0.38 | 0.0262 | | |

| Word stem | $\beta$ cent. | p cent. | $\beta$ status | p status |
|---|---|---|---|---|
| sit | 0.29 | 0.0402 | | |
| alright | 0.28 | 0.0028 | 0.23 | <.001 |
| gonna | 0.25 | <.001 | 0.11 | <.001 |
| caus | 0.25 | 0.0104 | | |
| what | 0.22 | 0.0234 | | |
| you | 0.22 | 0.0148 | | |
| right | 0.18 | 0.0162 | 0.12 | 0.0006 |
| like | -0.09 | 0.0042 | | |
| gui | -0.1 | 0.0404 | | |
| yeah | -0.1 | 0.0006 | | |
| go | -0.17 | 0.0342 | -0.09 | 0.0328 |
| kid | -0.19 | 0.009 | | |
| wai | -0.23 | 0.0102 | | |
| feel | -0.24 | 0.007 | | |
| point | -0.3 | 0.0428 | | |
| plan | -0.31 | 0.0442 | | |
| awai | -0.54 | 0.0006 | -0.28 | <.001 |
| ahead | -0.55 | 0.0468 | | |
| probabl | -0.59 | <.001 | -0.3 | <.001 |
| explain | -0.59 | 0.0424 | | |
| minut | -0.59 | 0.0252 | | |
| wear | -0.69 | 0.0122 | | |
| travel | -0.72 | 0.0056 | -0.32 | 0.0178 |
| confus | -0.78 | 0.0044 | -0.33 | 0.0176 |
| great | -0.98 | 0.0004 | | |
| eventu | -1.11 | 0.0012 | | |
| rest | | | 0.36 | 0.0256 |
| realli | | | 0.34 | 0.0206 |
| tri | | | 0.32 | 0.0168 |
| throw | | | 0.3 | 0.0154 |
| sister | | | 0.29 | 0.0012 |

| Word stem | $\beta$ cent. | p cent. | $\beta$ status | p status |
|---|---|---|---|---|
| super | | | 0.27 | 0.0084 |
| call | | | 0.27 | 0.0102 |
| di | | | 0.27 | 0.0058 |
| run | | | 0.25 | 0.038 |
| fine | | | 0.24 | 0.0088 |
| take | | | 0.2 | 0.0112 |
| involv | | | 0.19 | 0.0274 |
| kill | | | 0.18 | 0.0446 |
| sens | | | 0.16 | 0.0308 |
| movi | | | 0.1 | 0.0066 |
| thei | | | 0.1 | 0.0138 |
| RAREWORD | | | 0.05 | <.001 |
| think | | | 0.05 | 0.001 |
| but | | | -0.08 | 0.0238 |
| relat | | | -0.09 | 0.0172 |
| felt | | | -0.17 | 0.0154 |
| drive | | | -0.19 | 0.0372 |
| ladi | | | -0.2 | 0.0418 |
| wouldn | | | -0.22 | 0.0118 |
| big | | | -0.22 | 0.0276 |
| face | | | -0.22 | 0.0448 |
| reason | | | -0.23 | 0.026 |
| discuss | | | -0.24 | 0.0058 |
| place | | | -0.25 | 0.0084 |
| mind | | | -0.26 | 0.0316 |
| hard | | | -0.31 | 0.0308 |
| shot | | | -0.32 | 0.036 |
| show | | | -0.36 | 0.0024 |

**Supplementary Table 3:** Regression results for categorical speech turn type ratings. For descriptions of speech turn types, see Supplementary Table X. For each DV, a single hierarchical regression model was trained with rated speech turn type as a categorical predictor, the number of words spoken by the speaker as a control predictor, and random intercepts for speaker and rater identity. The most frequently occurring speech turn type, “propose”, was used as the reference category. For both models n=16,057 speech turns.

| Predictor | t | est. df | p | $\beta$ | [2.5% | 97.5%] | DV |
|---|---|---|---|---|---|---|---|
| propose (Intercept) | -1.8 | 10.368 | 0.101 | -0.785 | -1.675 | 0.106 | PCA Centrality |
| accept | 0.269 | 16023.7 | 0.788 | 0.006 | -0.039 | 0.051 | PCA Centrality |
| attend_movie | -0.041 | 16023.5 | 0.967 | -0.002 | -0.078 | 0.075 | PCA Centrality |
| attend_speaker | 3.353 | 16023.1 | 0.001 | 0.211 | 0.088 | 0.334 | PCA Centrality |
| clarify | 3.235 | 16023.3 | 0.001 | 0.155 | 0.061 | 0.249 | PCA Centrality |
| edit | -3.018 | 16023.4 | 0.003 | -0.129 | -0.213 | -0.045 | PCA Centrality |
| order | 1.507 | 16023.7 | 0.132 | 0.057 | -0.017 | 0.13 | PCA Centrality |
| other | 0.113 | 16023.5 | 0.91 | 0.003 | -0.044 | 0.049 | PCA Centrality |
| reject_hard | 1.349 | 16023.2 | 0.177 | 0.064 | -0.029 | 0.156 | PCA Centrality |
| reject_soft | 1.875 | 16023.1 | 0.061 | 0.089 | -0.004 | 0.181 | PCA Centrality |
| repair | 1.257 | 16023 | 0.209 | 0.255 | -0.143 | 0.653 | PCA Centrality |
| propose (Intercept) | -4.504 | 9.348 | 0.001 | -0.806 | -1.173 | -0.438 | Perceived status |
| accept | -1.688 | 16023.6 | 0.091 | -0.013 | -0.028 | 0.002 | Perceived status |
| attend_movie | -0.135 | 16023.4 | 0.893 | -0.002 | -0.027 | 0.024 | Perceived status |
| attend_speaker | 4.06 | 16023.1 | <.001 | 0.086 | 0.044 | 0.127 | Perceived status |
| clarify | 1.442 | 16023.3 | 0.149 | 0.023 | -0.008 | 0.055 | Perceived status |
| edit | -1.22 | 16023.4 | 0.223 | -0.017 | -0.046 | 0.011 | Perceived status |
| order | 7.939 | 16023.6 | <.001 | 0.1 | 0.075 | 0.124 | Perceived status |
| other | 2.68 | 16023.5 | 0.007 | 0.021 | 0.006 | 0.037 | Perceived status |
| reject_hard | -0.751 | 16023.2 | 0.453 | -0.012 | -0.043 | 0.019 | Perceived status |
| reject_soft | 2.42 | 16023.1 | 0.016 | 0.038 | 0.007 | 0.069 | Perceived status |
| repair | -0.67 | 16023 | 0.503 | -0.046 | -0.179 | 0.088 | Perceived status |

**Supplementary Table 4:** Categorical speech turn types. Raters classified each speech turn as one of the listed types. Count lists the number of speech turns classified as each type across all conversations. “Clarify” indicates clarification of meaning, e.g., “What do you mean by that?”, whereas “repair” indicates clarification of what was spoken, e.g., “Could you repeat that?”

| Speech turn type | Count | Description |
|---|---|---|
| propose | 5586 | Proposing an explanation of a movie clip |
| accept | 3272 | Accepting another speaker’s proposal |
| attend_movie | 812 | Directing attention to movie clip content |
| attend_speaker | 283 | Directing attention to another speaker |
| clarify | 509 | Requesting clarification of what another speaker meant |
| edit | 654 | Suggesting a change to another speaker’s proposal |
| order | 895 | Giving an order and/or setting a conversation subject |
| other | 2962 | Not captured by the rating rubric |
| reject_hard | 526 | Explicitly rejecting another speaker’s proposal |
| reject_soft | 521 | Implicitly rejecting another speaker’s proposal |
| repair | 26 | Requesting clarification of what another speaker said |

**Supplementary Table 5:** Regression results for continuous speech turn ratings. For descriptions of continuous predictors, see Supplementary Table X. Separate analyses were conducted for each predictor and each DV, ensuring that the results capture variance shared across predictors. Each model included the number of words spoken by the speaker as a control predictor, and random intercepts for speaker and rater identity. For all models n=16,057 speech turns.

| Predictor | t | est. df | p | $\beta$ | [2.5% | 97.5%] | DV |
|---|---|---|---|---|---|---|---|
| content_accepted | -2.615 | 9537.4 | 0.045 | -0.011 | -0.02 | -0.003 | PCA centrality |
| event_ignored | -1.052 | 15882.7 | 0.44 | -0.004 | -0.011 | 0.003 | PCA centrality |
| speaker_confidence | 2.692 | 15981 | 0.043 | 0.018 | 0.005 | 0.031 | PCA centrality |
| speaker_deference | -1.226 | 5299.19 | 0.44 | -0.009 | -0.024 | 0.006 | PCA centrality |
| matter_fact | 1.924 | 5581.59 | 0.163 | 0.014 | -<.001 | 0.028 | PCA centrality |
| joke_intended | 2.073 | 1309.42 | 0.154 | 0.019 | 0.001 | 0.037 | PCA centrality |
| content_accepted | 2.018 | 9538.11 | 0.087 | 0.004 | <.001 | 0.007 | Perceived status |
| event_ignored | -6.238 | 15975.1 | <.001 | -0.009 | -0.012 | -0.006 | Perceived status |
| speaker_confidence | 8.564 | 15990.5 | <.001 | 0.024 | 0.018 | 0.029 | Perceived status |
| speaker_deference | -1.159 | 5561.27 | 0.247 | -0.004 | -0.011 | 0.003 | Perceived status |
| matter_fact | 2.248 | 6222.96 | 0.074 | 0.007 | 0.001 | 0.013 | Perceived status |

| Predictor | t | est. df | p | $\beta$ | [2.5% | 97.5%] | DV |
|---|---|---|---|---|---|---|---|
| joke_intended | 2.851 | 1306.03 | 0.018 | 0.011 | 0.003 | 0.018 | Perceived status |

**Supplementary Table 6:** Continuous speech turn properties. Each speech turn was rated 0–10. Raters were instructed to always rate content_accepted and event_ignored, and to rate the other predictors when relevant.

| Predictor | Description |
|---|---|
| content_accepted | The extent this content was accepted by the group |
| event_ignored | The extent this speech turn was ignored by others |
| speaker_confidence | The perceived confidence of the speaker |
| speaker_deference | The perceived deference of the spaker to other speakers |
| matter_fact | The extent that this speech turn concerns a matter of fact |
| joke_intended | The extent that this speech turn was intended as a joke |

**Supplementary Table 7:** Structure of fMRI sessions.

| Session | Run # | Stimulus | TRs | Time (s) | Sound |
|---|---|---|---|---|---|
| before | 1 | Birth | 334 | 242.818 | Off |
| before | 2 | The Assassination of Jesse James by the Coward Robert Ford | 250 | 181.75 | Off |
| before | 3 | Sexy Beast | 388 | 282.076 | Off |
| before | 4 | The Master | 466 | 338.782 | Off |
| before | 5 | Y tu mamá también | 368 | 267.536 | Off |
| before | 6 | Michael Clayton (hyperalignment) | 1249 | 908.023 | On |
| before | 7 | High Maintenance, “Tick” (hyperalignment) | 1249 | 908.023 | On |
| after | 1 | Birth (repeat) | 334 | 242.818 | Off |
| after | 2 | The Assassination of Jesse James by the Coward Robert Ford (repeat) | 250 | 181.75 | Off |
| after | 3 | Sexy Beast (repeat) | 388 | 282.076 | Off |
| after | 4 | The Master (repeat) | 466 | 338.782 | Off |
| after | 5 | Y tu mamá también (repeat) | 368 | 267.536 | Off |
| after | 6 | Birth (novel) | 367 | 266.809 | Off |

| Session | Run # | Stimulus | TRs | Time (s) | Sound |
|---|---|---|---|---|---|
| after | 7 | The Assassination of Jesse James by the Coward Robert Ford (novel) | 412 | 299.524 | Off |
| after | 8 | Sexy Beast (novel) | 403 | 292.981 | Off |
| after | 9 | The Master (novel) | 326 | 237.002 | Off |
| after | 10 | Y tu mamá también (novel) | 452 | 328.604 | Off |

**Supplementary Table 8:** Peak beta values for whole-brain regression analyses across all conversation groups and movies, corrected for multiple comparisons (cluster forming threshold: p=.05, minimum cluster size: 115 voxels, p<.05 corrected). This table does not show group- and movie-specific results; see Supplementary Table 6. See *Methods* for complete descriptions of each analysis. For the neural influence analysis, the group column additionally specifies whether ego or alter centrality was mapped and which centrality measure was used (EC: eigenvector centrality; br.: brokerage; cent.: PCA centrality). Atlas labels from.[1]

| x | y | z | Atlas label | $\beta$ | Analysis |
|---|---|---|---|---|---|
| 58.0 | -68.0 | 6.0 | R Middle temporal gyrus (T2) | .05 | Conversation |
| -5.0 | -68.0 | 9.0 | L Middle occipital gyrus (O2, lateral occipital gyrus) | .05 | Conversation |
| -26.0 | -53.0 | 69.0 | L Superior parietal lobule (lateral part of P1) | .05 | Conversation |
| -44.0 | -68.0 | 6.0 | L Anterior occipital sulcus and preoccipital notch (temporo-occipital incisure) | -.12 | Control |
| 34.0 | -59.0 | 57.0 | R Superior parietal lobule (lateral part of P1) | -.09 | Control |
| 4.0 | -56.0 | 15.0 | R Superior temporal sulcus (parallel sulcus) | -.09 | Control |
| 37.0 | 1.0 | 36.0 | R Inferior part of the precentral sulcus | -.07 | Control |
| 61.0 | -8.0 | -18.0 | R Middle temporal gyrus (T2) | .06 | Control |
| 4.0 | -5.0 | 69.0 | R Superior frontal gyrus (F1) | .07 | Conversation, unseen |
| -71.0 | -26.0 | -6.0 | L Middle temporal gyrus (T2) | -.04 | Influ. EC ego |
| -32.0 | -5.0 | 6.0 | L Superior parietal lobule (lateral part of P1) | .05 | Influ. EC alter |
| -5.0 | -86.0 | .0 | L Lingual gyrus, lingual part of the medial occipito-temporal gyrus, (O5) | .06 | Influ. EC alter |

| x | y | z | Atlas label | $\beta$ | Analysis |
|---|---|---|---|---|---|
| 61.0 | -35.0 | -3.0 | R Middle temporal gyrus (T2) | -.02 | Influ. PCA ego |
| 1.0 | -8.0 | -9.0 | R Lingual gyrus, lingual part of the medial occipito-temporal gyrus, (O5) | .04 | Influ. PCA alter |
| 31.0 | -38.0 | 69.0 | R Postcentral gyrus | .04 | Influ. PCA alter |
| -8.0 | -59.0 | 66.0 | L Precuneus (medial part of P1) | .04 | Influ. PCA alter |
| -2.0 | -14.0 | 66.0 | L Superior part of the precentral sulcus | .03 | Influ. PCA alter |
| 52.0 | -11.0 | -9.0 | R Superior temporal sulcus (parallel sulcus) | .02 | Influ. PCA alter |

## Supplementary note 1

### Cluster reports

Each cluster report below is labeled by movie clip and group and contains the following elements. Upper left: multi-dimensional scaling plot showing relative distances between participants' patterns of brain activity before conversation (yellow) and after conversation (orange). Bottom left: A spatially contiguous cluster of active voxels that survived multiple comparisons correction for the indicated group and movie clip. Upper middle: Probability of Neurosynth terms given the pattern of brain activity. Right top and middle: Mean BOLD activation over time, with each group member shown in a different color. Right bottom: Change in group-level ISC over time.

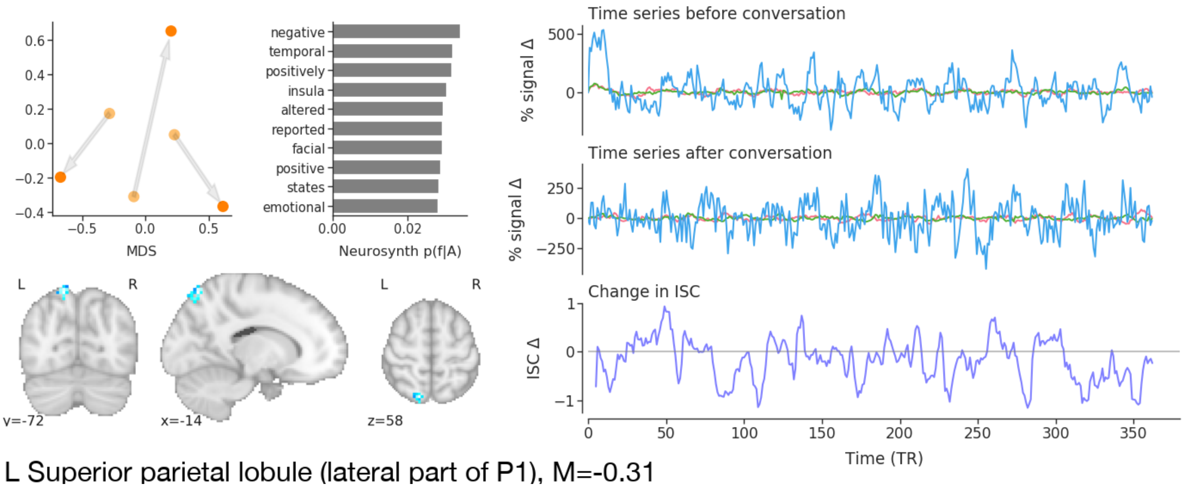


**Supplementary Figure 14:** Change in ISC. Movie clip: Y tu mamá también. Group: A

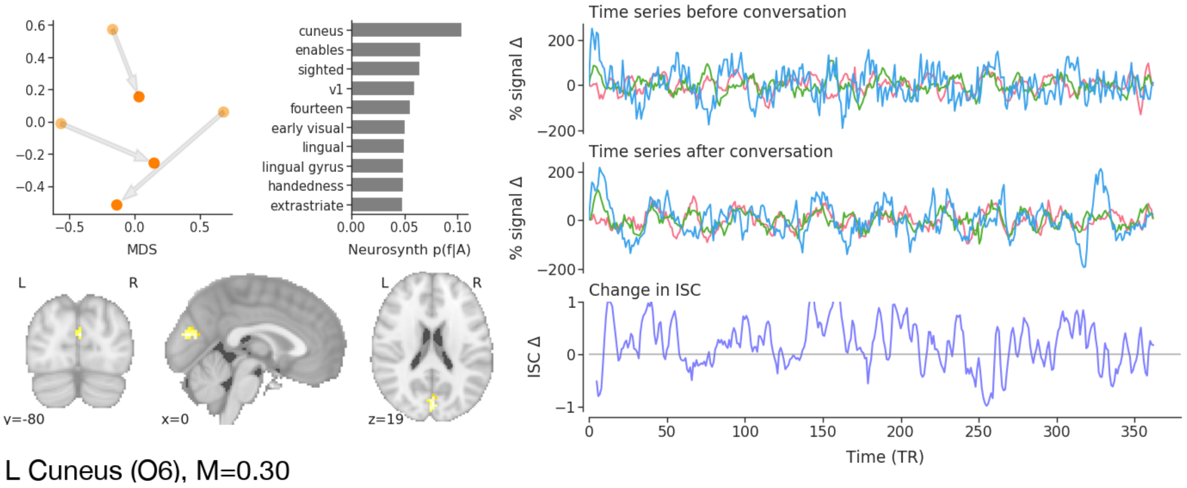


**Supplementary Figure 15:** Change in ISC. Movie clip: Y tu mamá también. Group: B

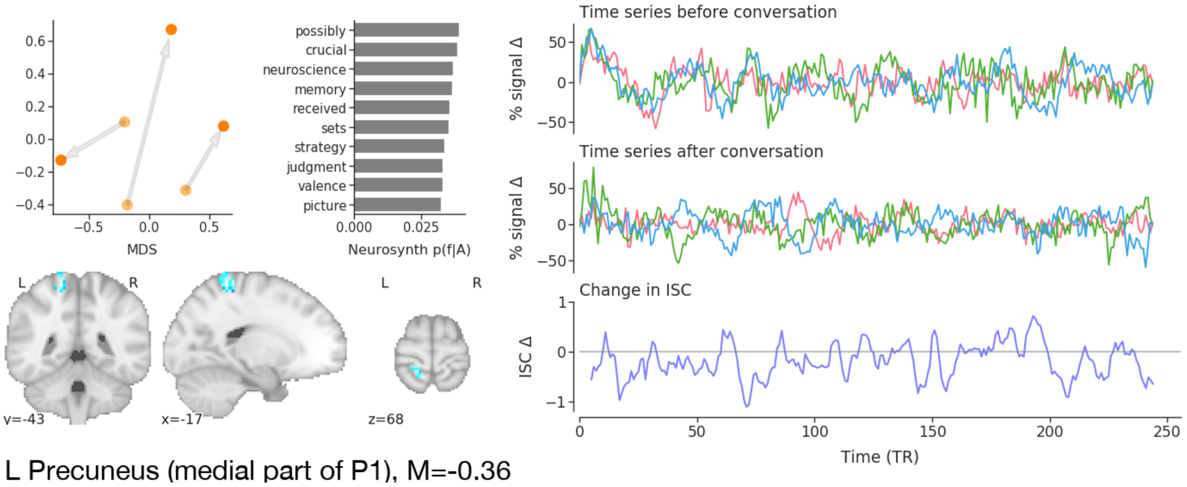


**Supplementary Figure 16:** Change in ISC. Movie clip: The Assassination of Jesse James by the Coward Robert Ford. Group: C

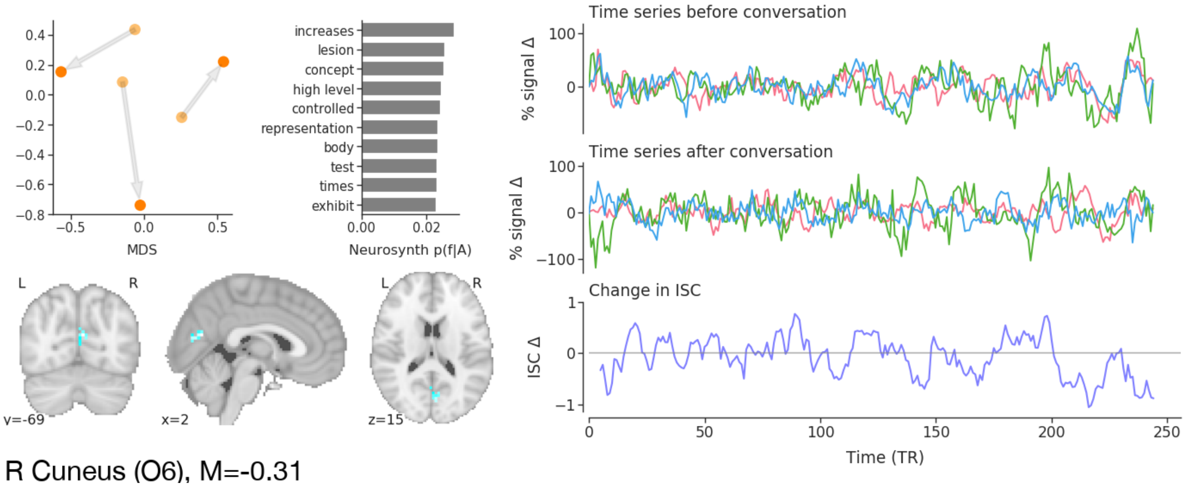


**Supplementary Figure 17:** Change in ISC. Movie clip: The Assassination of Jesse James by the Coward Robert Ford. Group: C

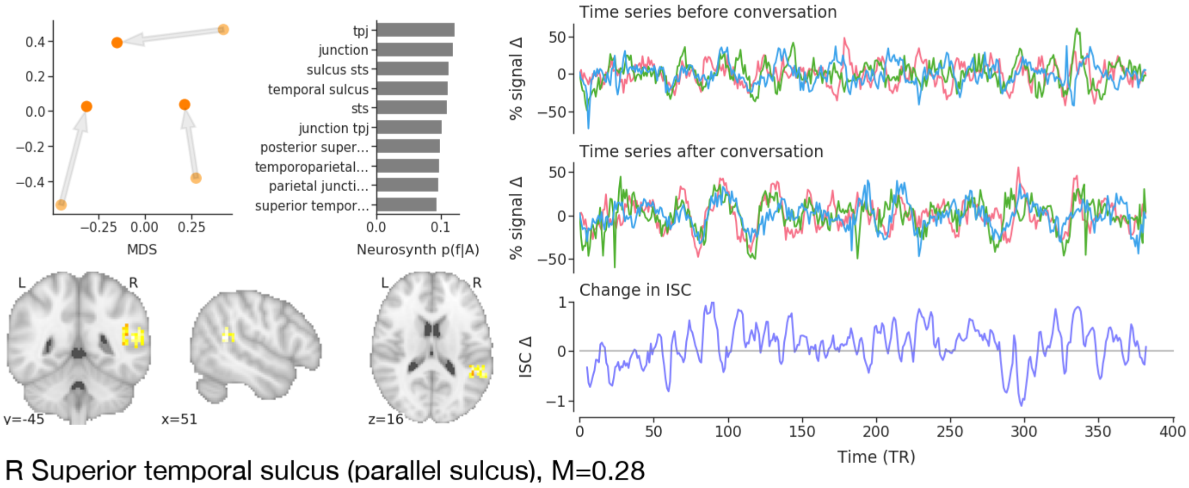


**Supplementary Figure 18:** Change in ISC. Movie clip: Sexy Beast. Group: C

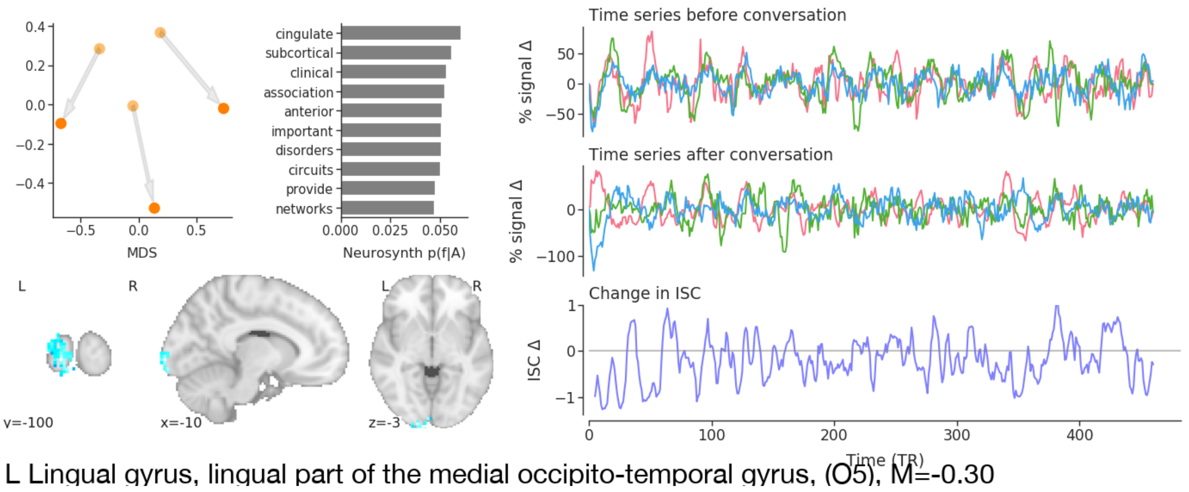


**Supplementary Figure 19:** Change in ISC. Movie clip: The Master. Group: C

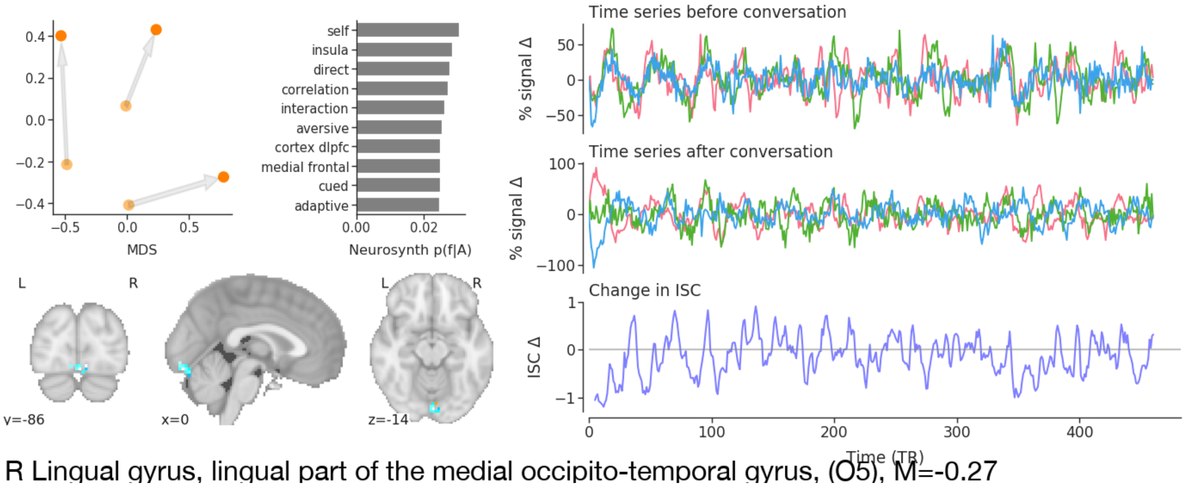


**Supplementary Figure 20:** Change in ISC. Movie clip: The Master. Group: C

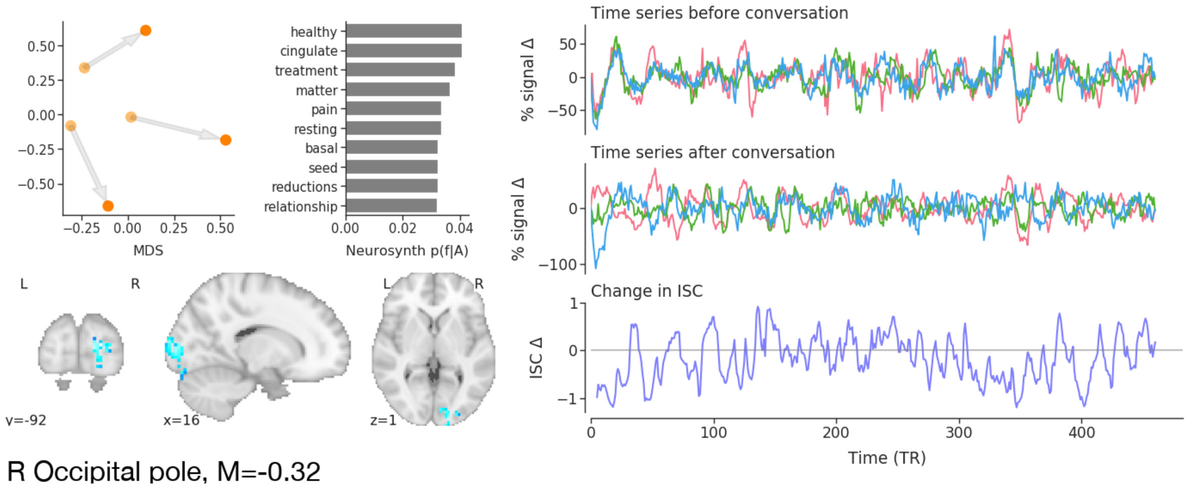


**Supplementary Figure 21:** Change in ISC. Movie clip: The Master. Group: C

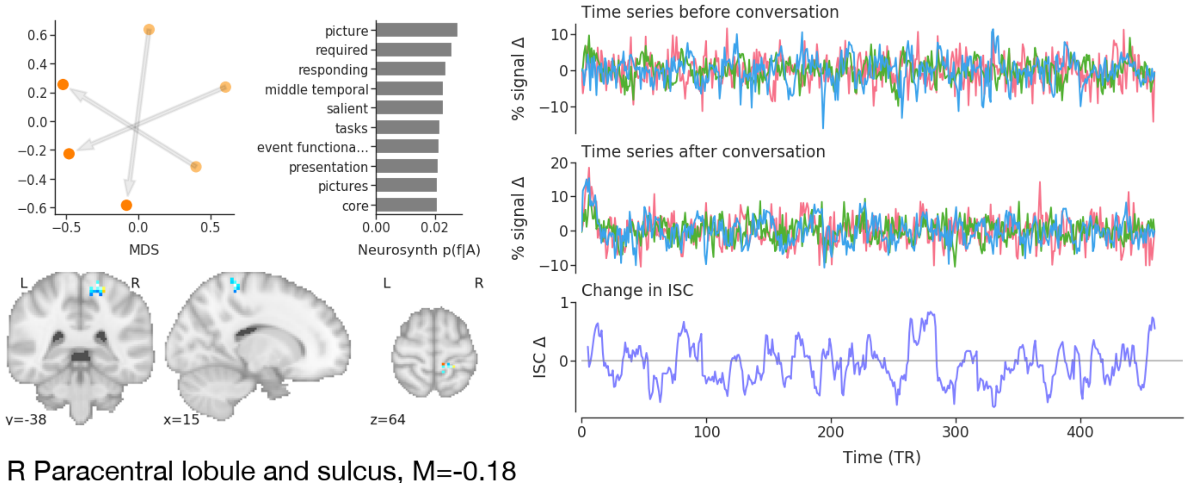


**Supplementary Figure 22:** Change in ISC. Movie clip: The Master. Group: C

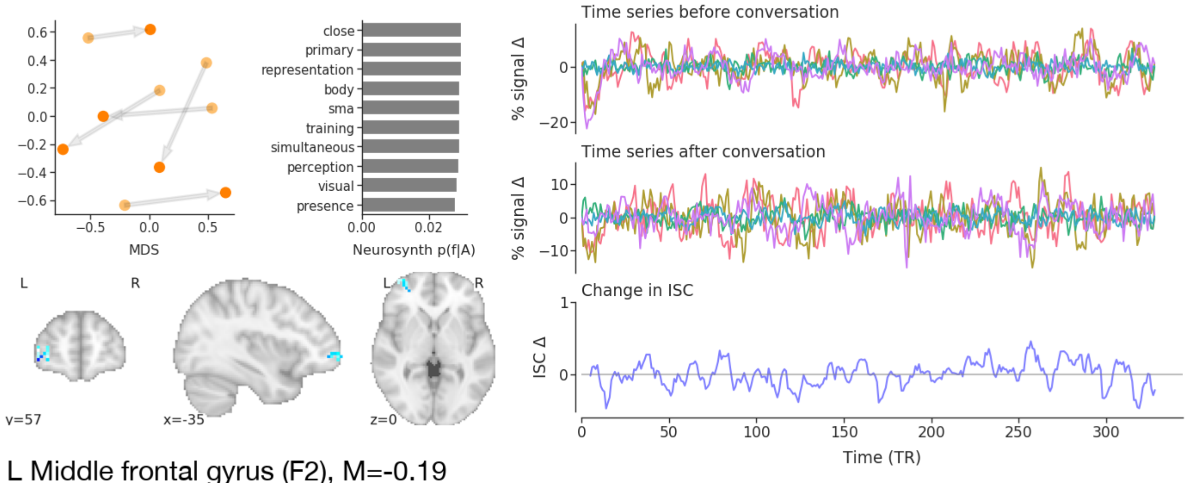


**Supplementary Figure 23:** Change in ISC. Movie clip: Birth. Group: D

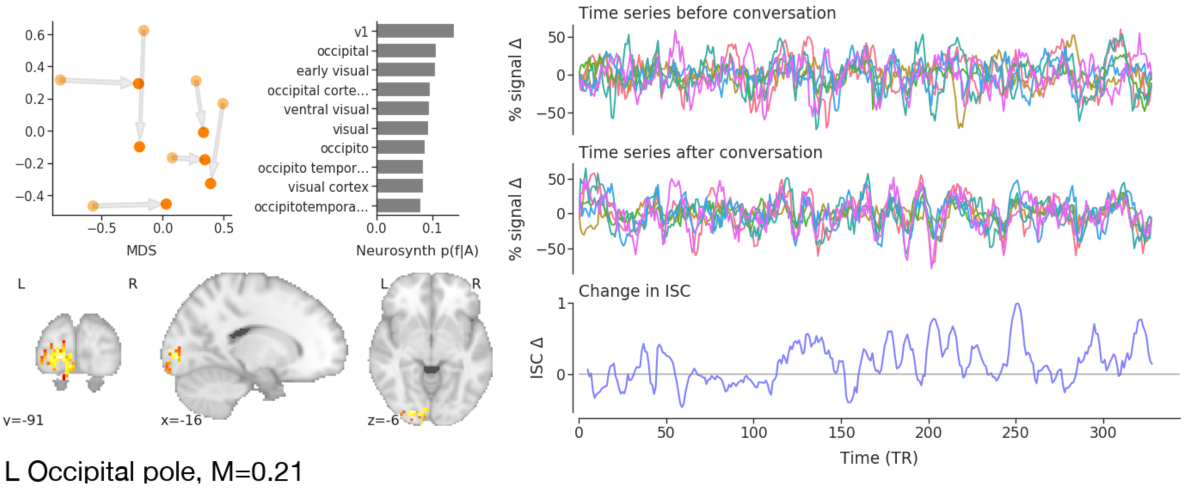


**Supplementary Figure 24:** Change in ISC. Movie clip: Birth. Group: E

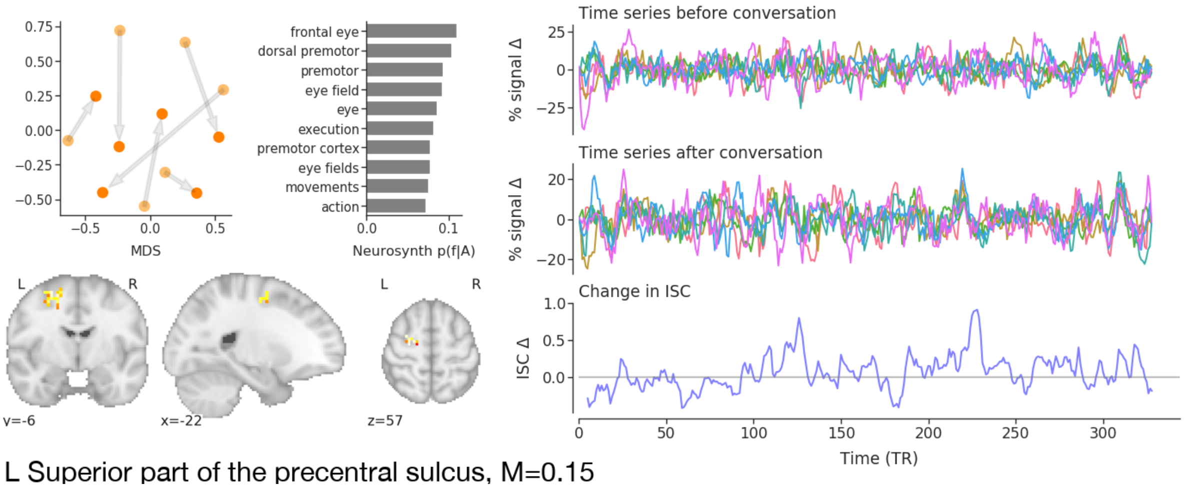


**Supplementary Figure 25:** Change in ISC. Movie clip: Birth. Group: E

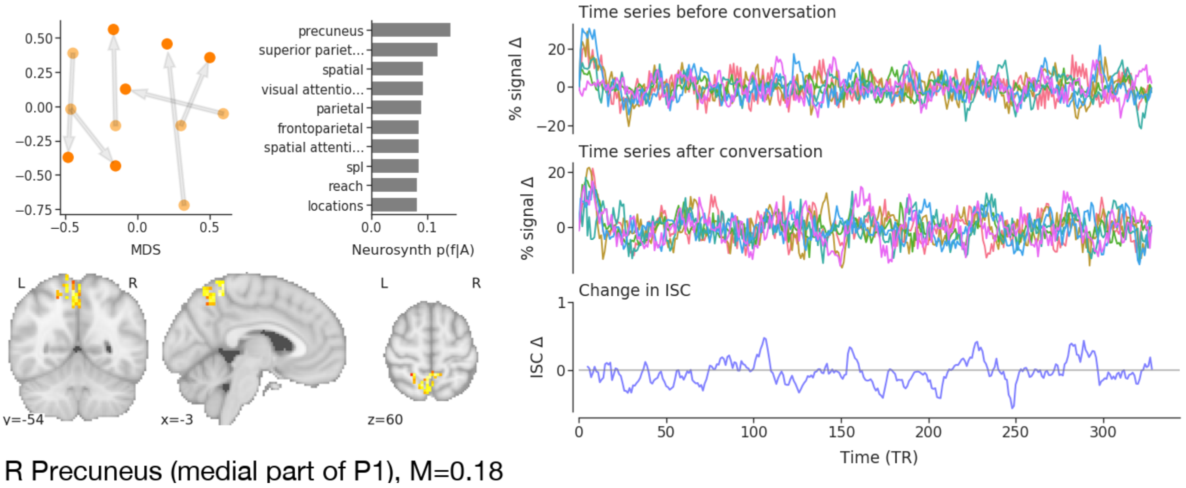


**Supplementary Figure 26:** Change in ISC. Movie clip: Birth. Group: E

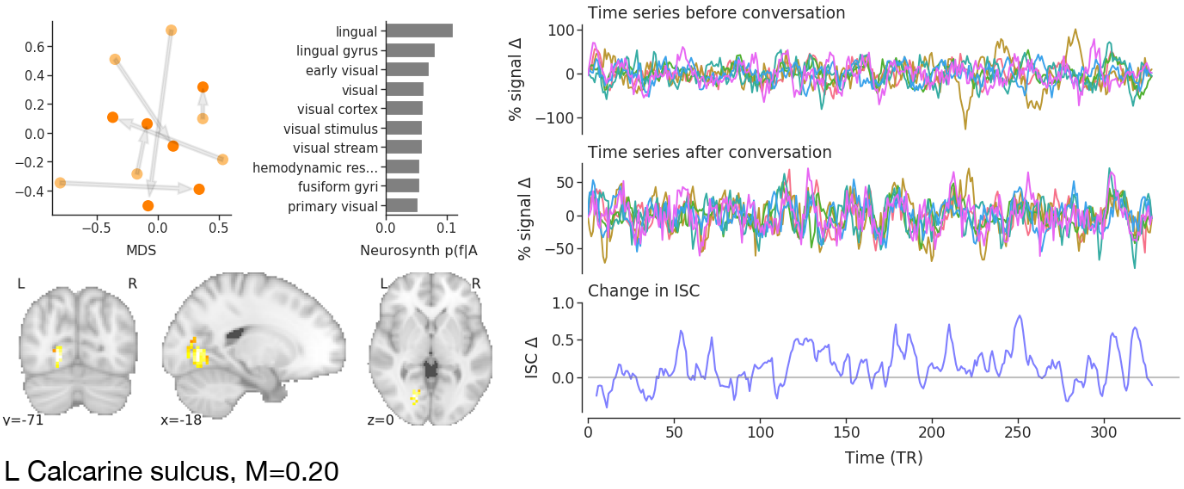


**Supplementary Figure 27:** Change in ISC. Movie clip: Birth. Group: E

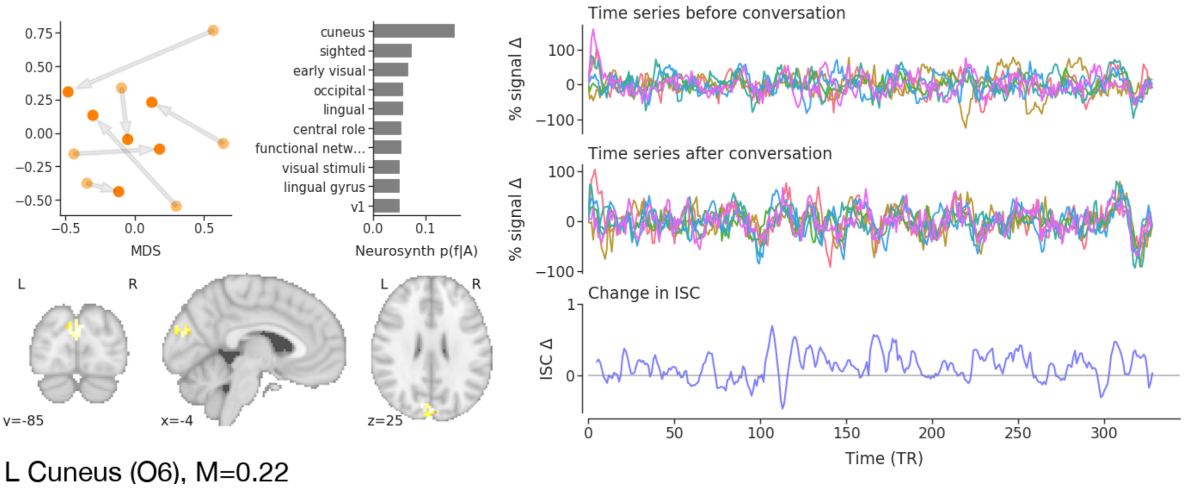


**Supplementary Figure 28:** Change in ISC. Movie clip: Birth. Group: E

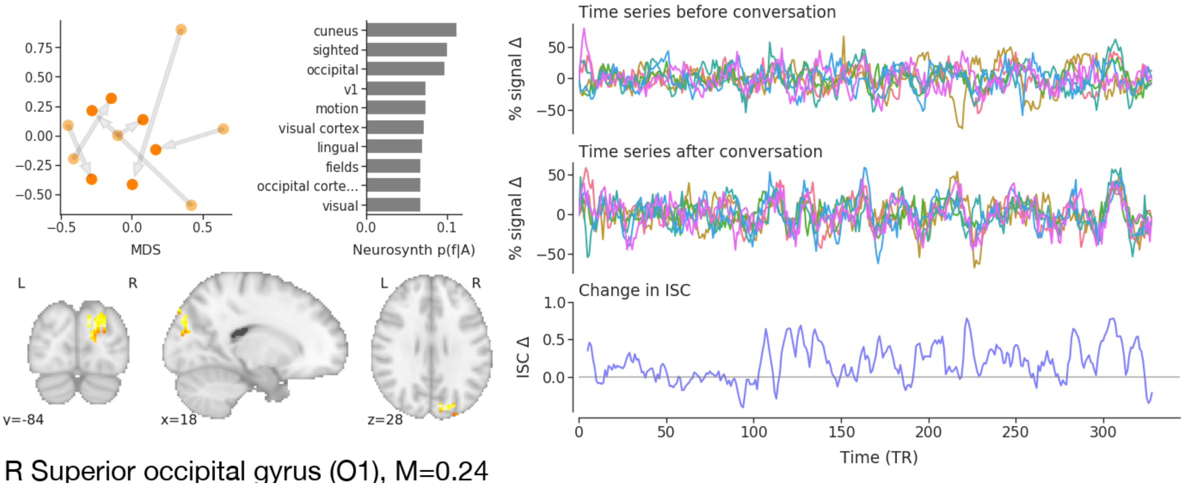


**Supplementary Figure 29:** Change in ISC. Movie clip: Birth. Group: E

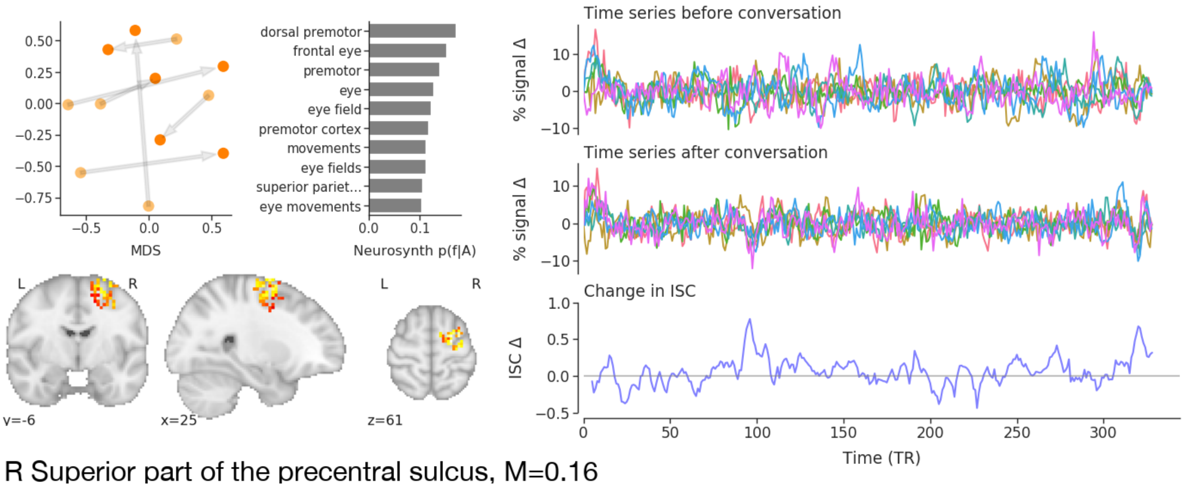


**Supplementary Figure 30:** Change in ISC. Movie clip: Birth. Group: E

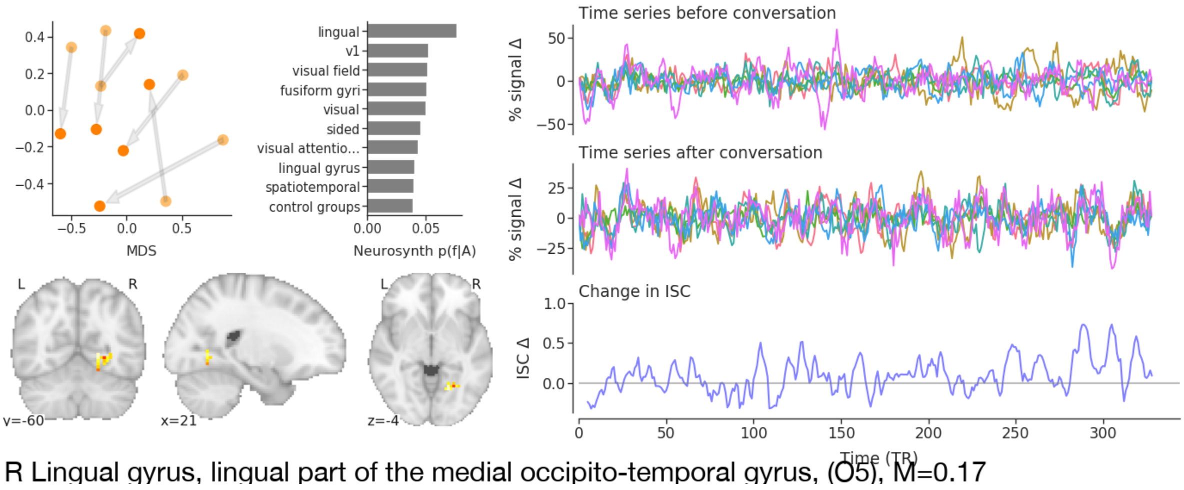


**Supplementary Figure 31:** Change in ISC. Movie clip: Birth. Group: E

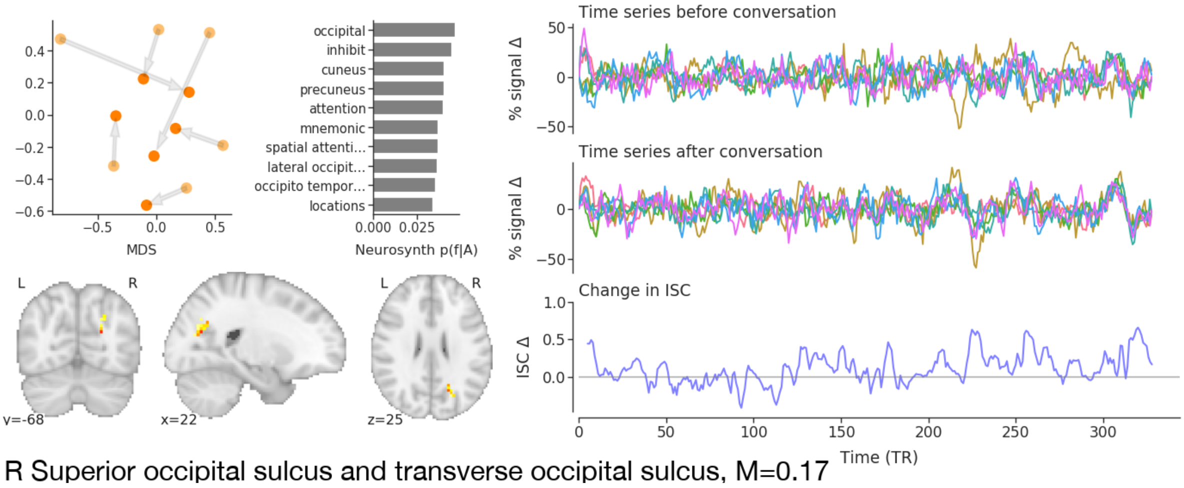


**Supplementary Figure 32:** Change in ISC. Movie clip: Birth. Group: E

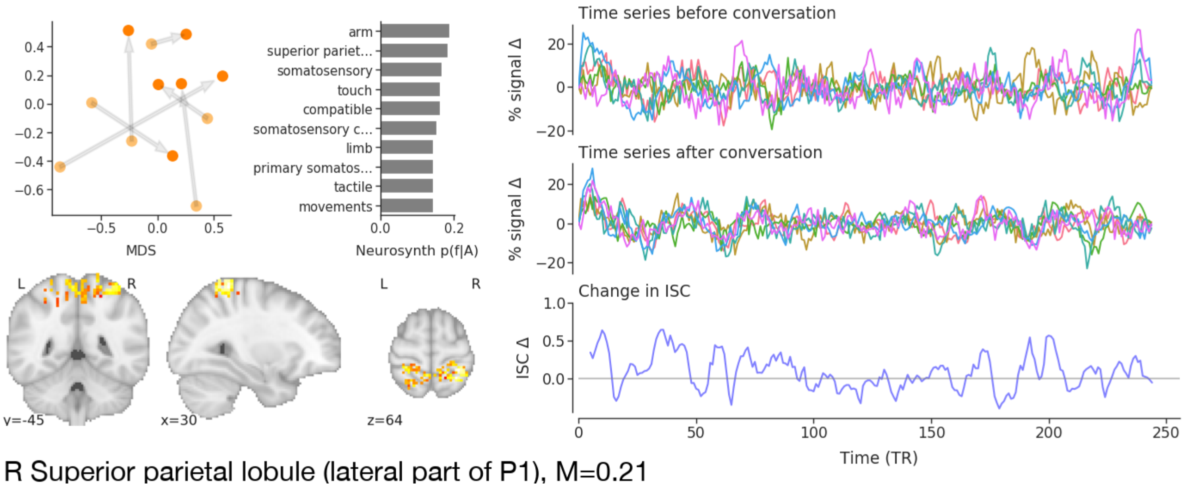


**Supplementary Figure 33:** Change in ISC. Movie clip: The Assassination of Jesse James by the Coward Robert Ford. Group: E

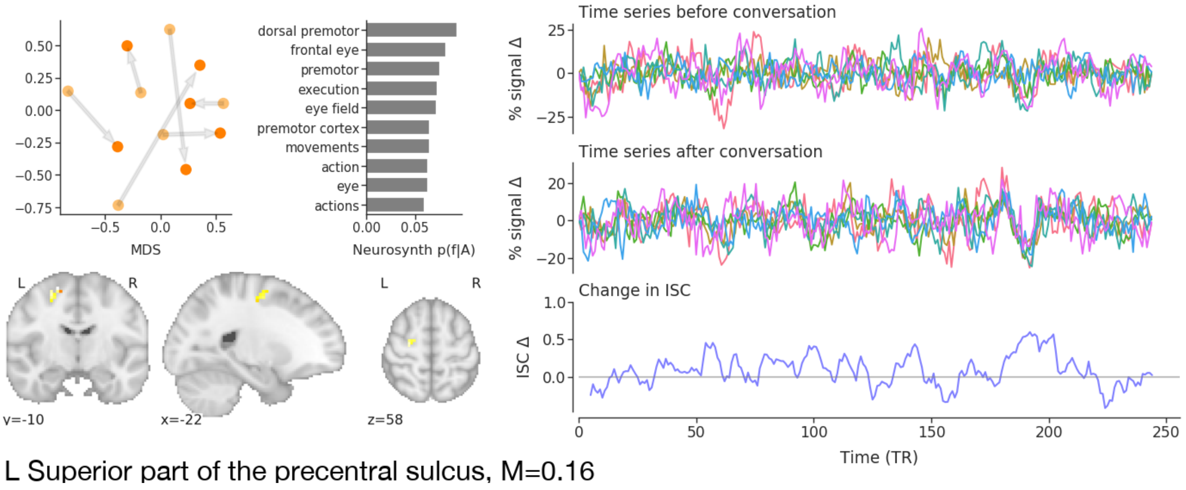


**Supplementary Figure 34:** Change in ISC. Movie clip: The Assassination of Jesse James by the Coward Robert Ford. Group: E

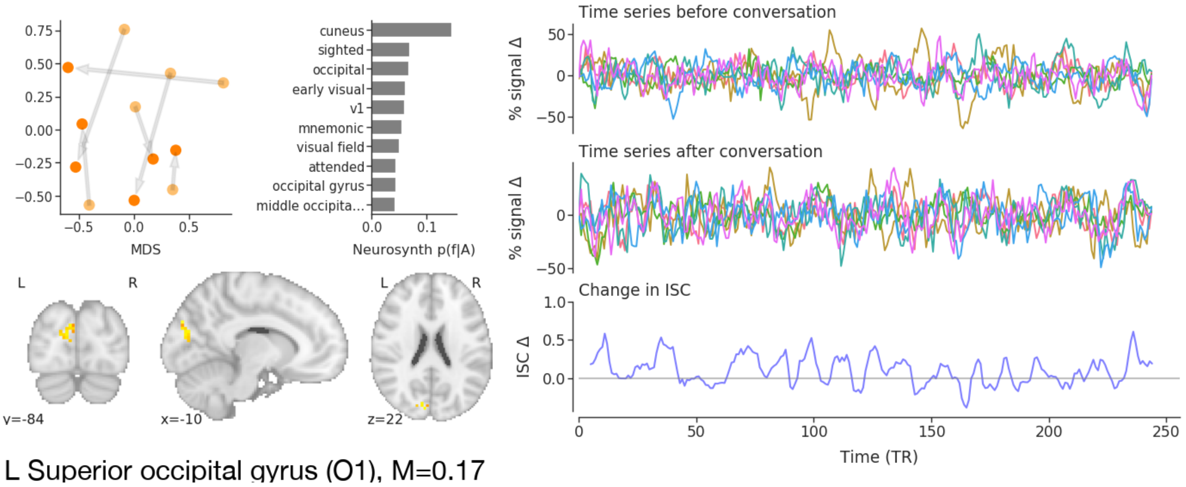


**Supplementary Figure 35:** Change in ISC. Movie clip: The Assassination of Jesse James by the Coward Robert Ford. Group: E

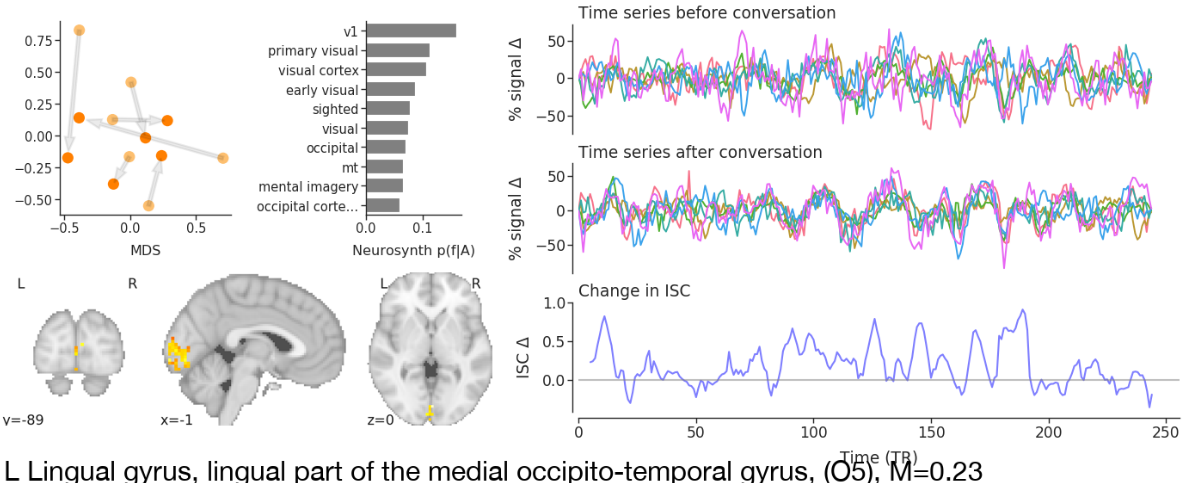


**Supplementary Figure 36:** Change in ISC. Movie clip: The Assassination of Jesse James by the Coward Robert Ford. Group: E

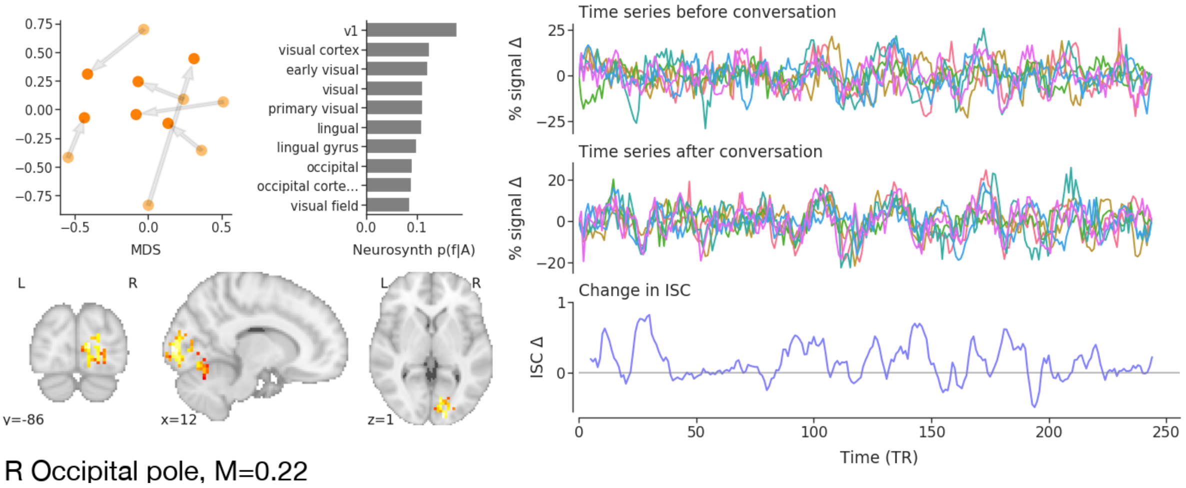


**Supplementary Figure 37:** Change in ISC. Movie clip: The Assassination of Jesse James by the Coward Robert Ford. Group: E

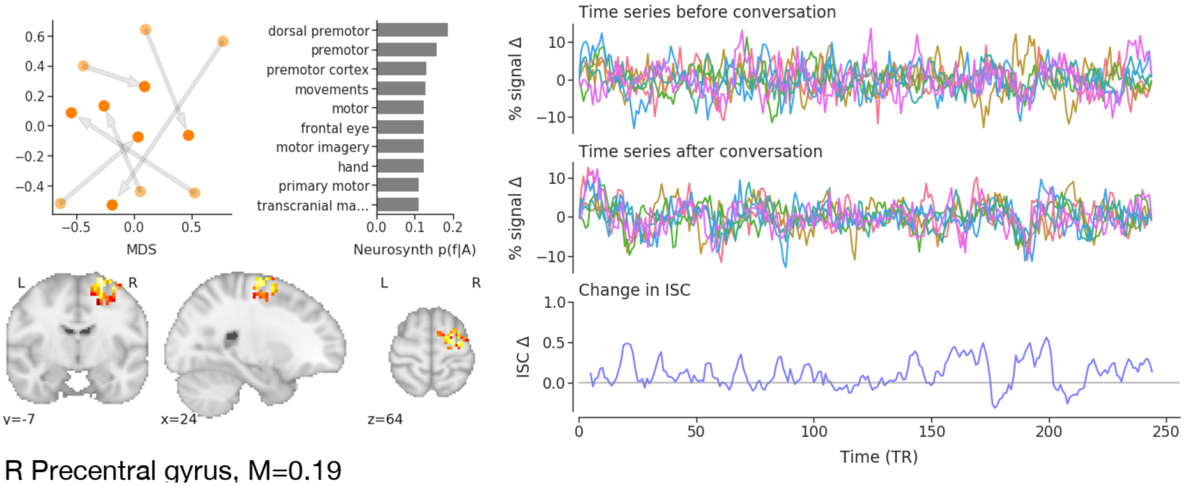


**Supplementary Figure 38:** Change in ISC. Movie clip: The Assassination of Jesse James by the Coward Robert Ford. Group: E

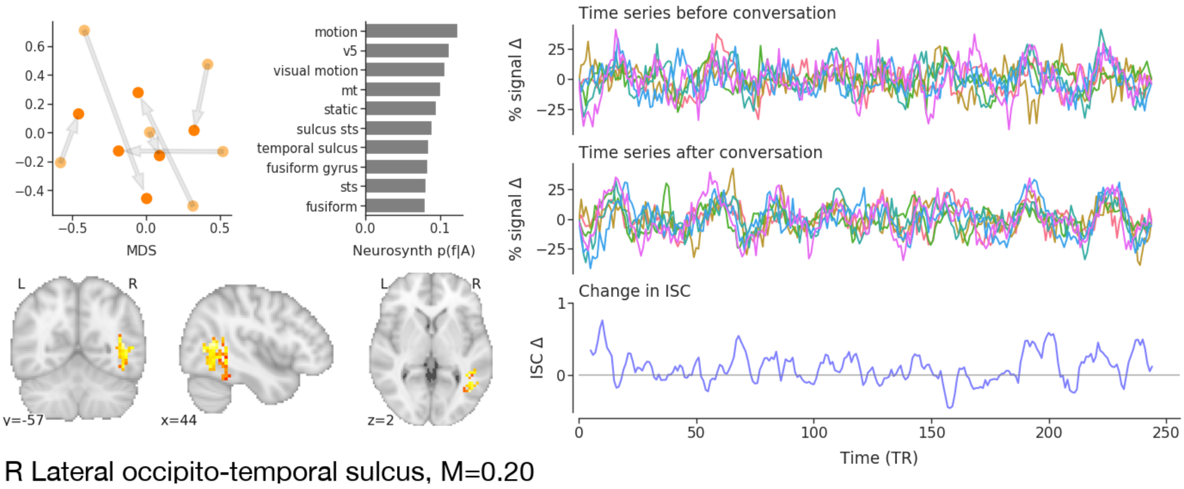


**Supplementary Figure 39:** Change in ISC. Movie clip: The Assassination of Jesse James by the Coward Robert Ford. Group: E

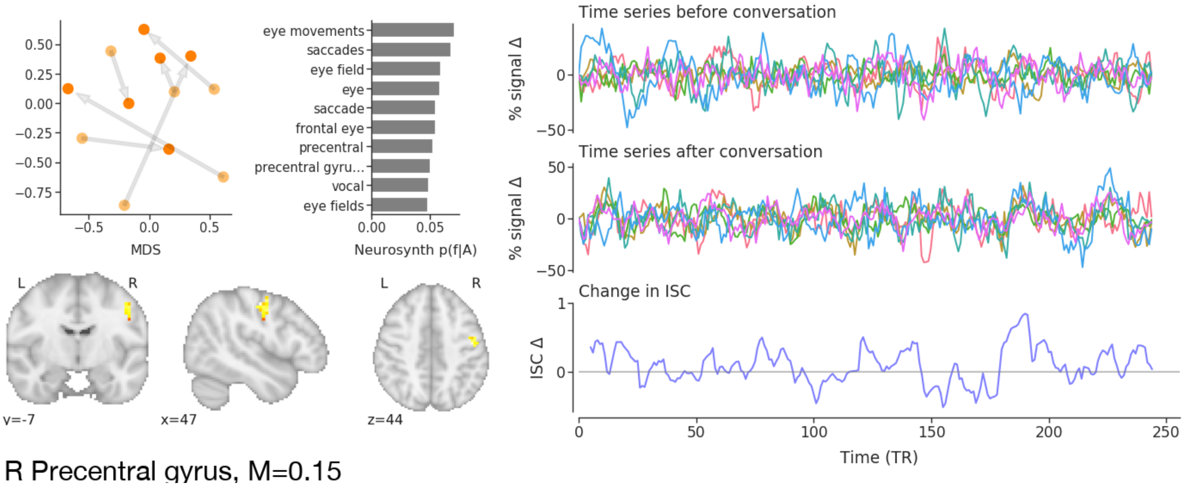


**Supplementary Figure 40:** Change in ISC. Movie clip: The Assassination of Jesse James by the Coward Robert Ford. Group: E

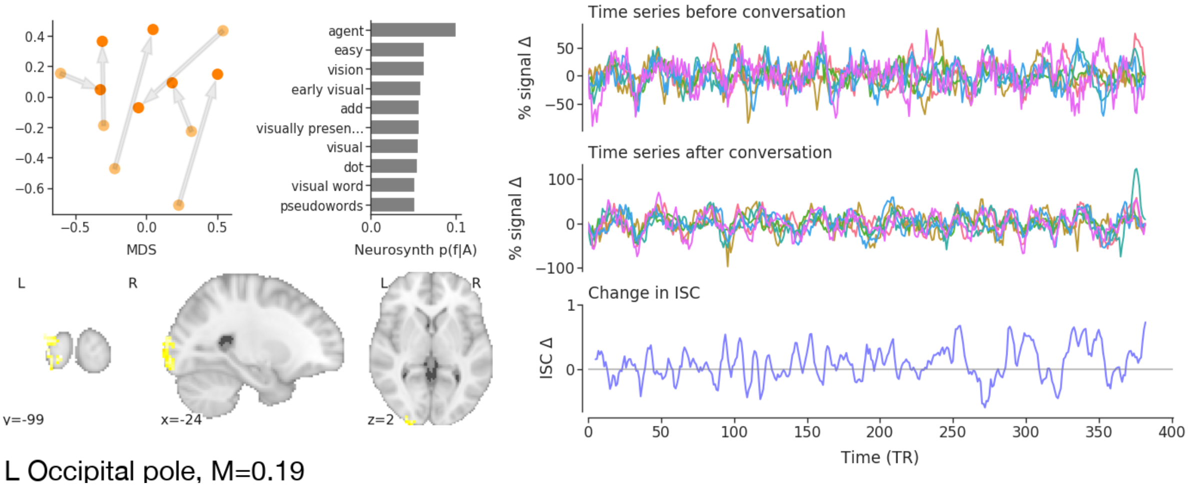


**Supplementary Figure 41:** Change in ISC. Movie clip: Sexy Beast. Group: E

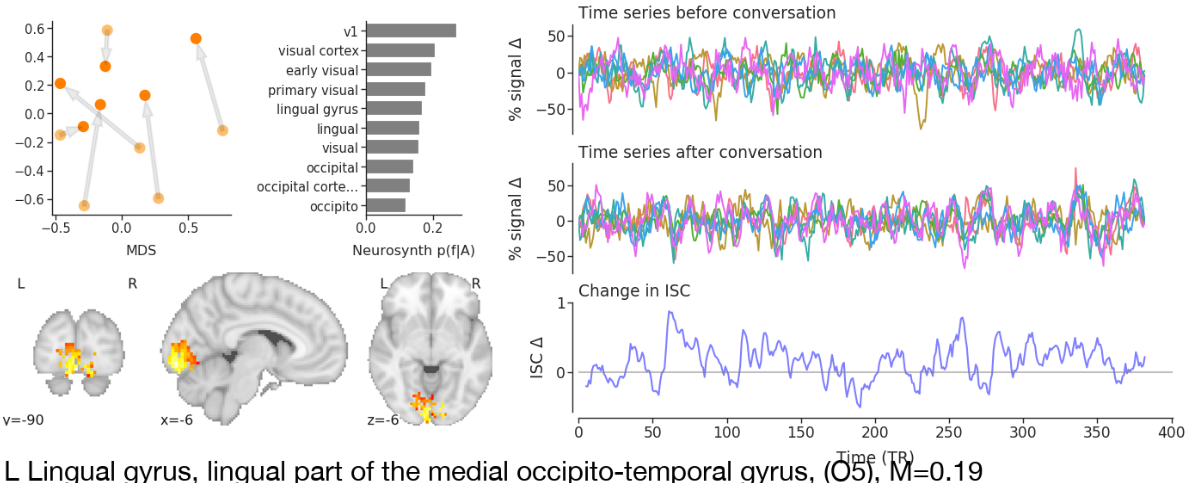


**Supplementary Figure 42:** Change in ISC. Movie clip: Sexy Beast. Group: E

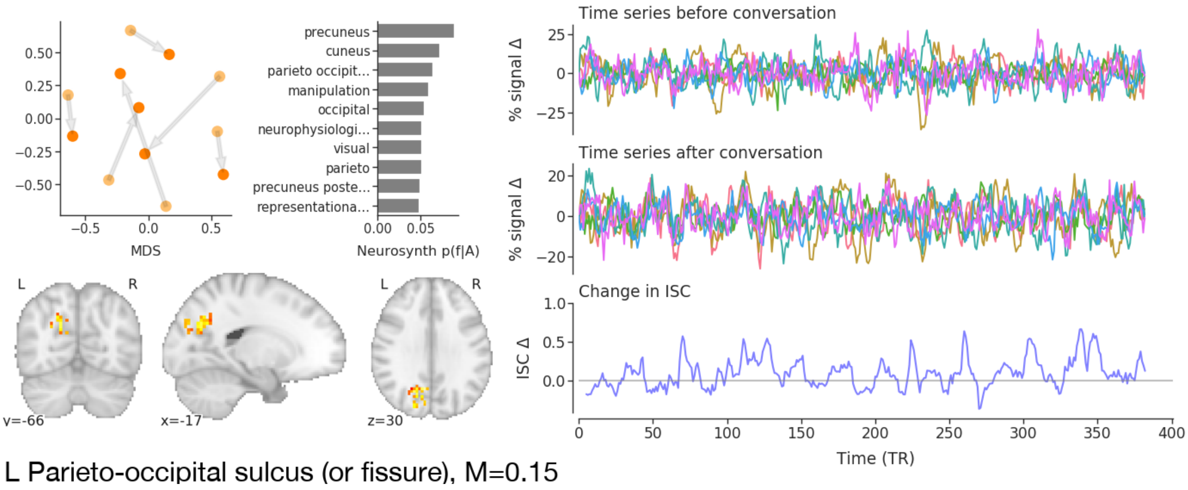


**Supplementary Figure 43:** Change in ISC. Movie clip: Sexy Beast. Group: E

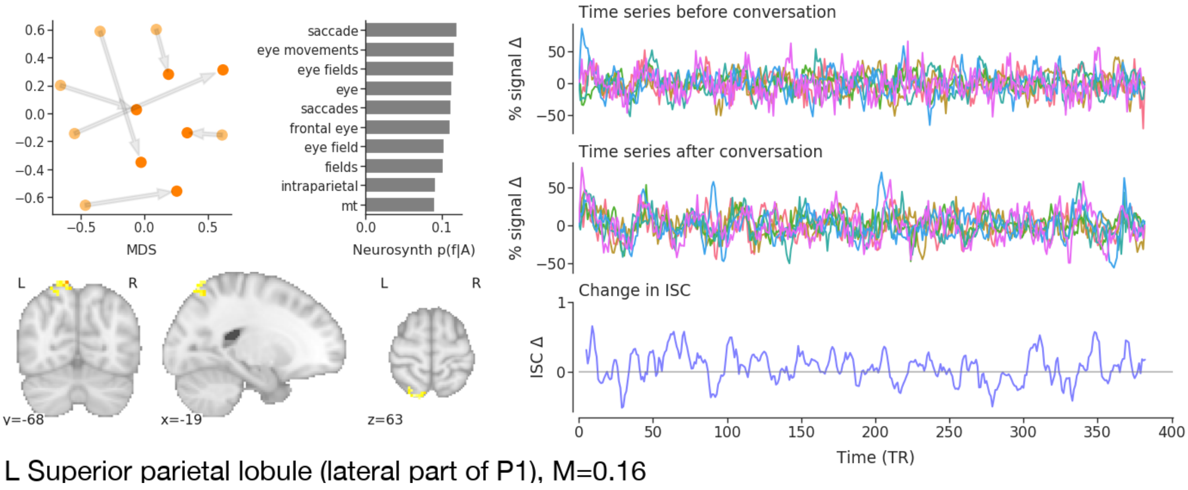


**Supplementary Figure 44:** Change in ISC. Movie clip: Sexy Beast. Group: E

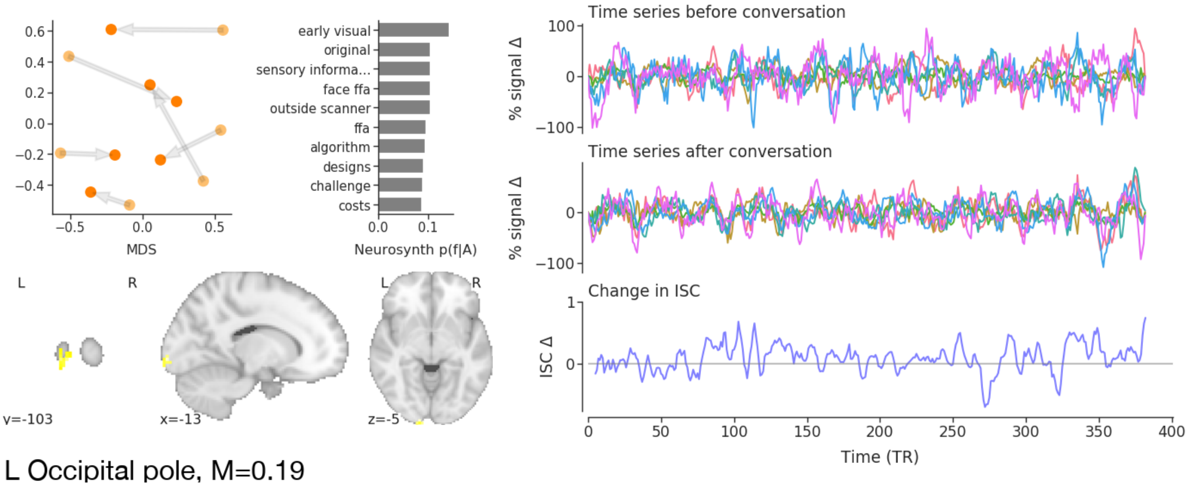


**Supplementary Figure 45:** Change in ISC. Movie clip: Sexy Beast. Group: E

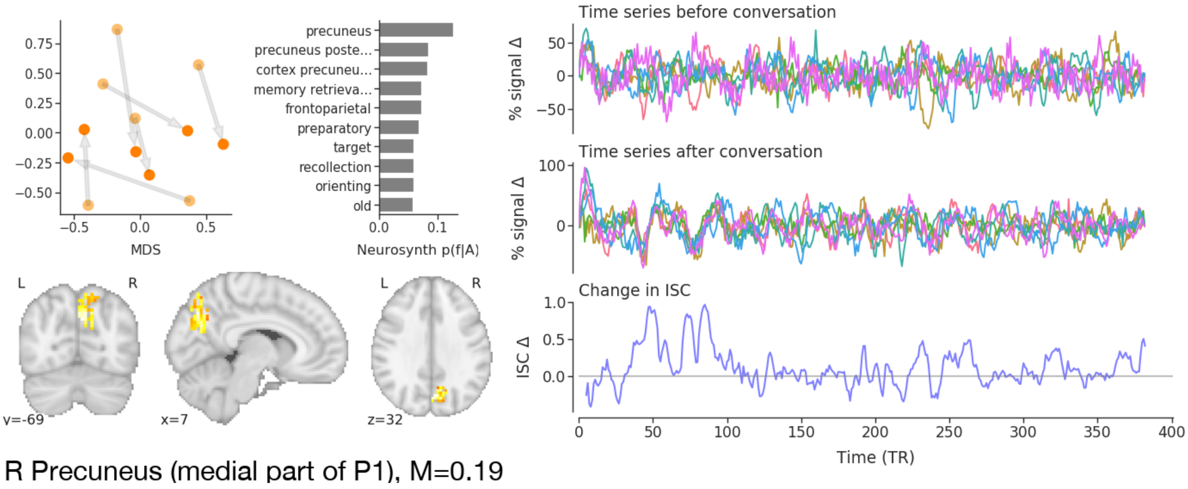


**Supplementary Figure 46:** Change in ISC. Movie clip: Sexy Beast. Group: E

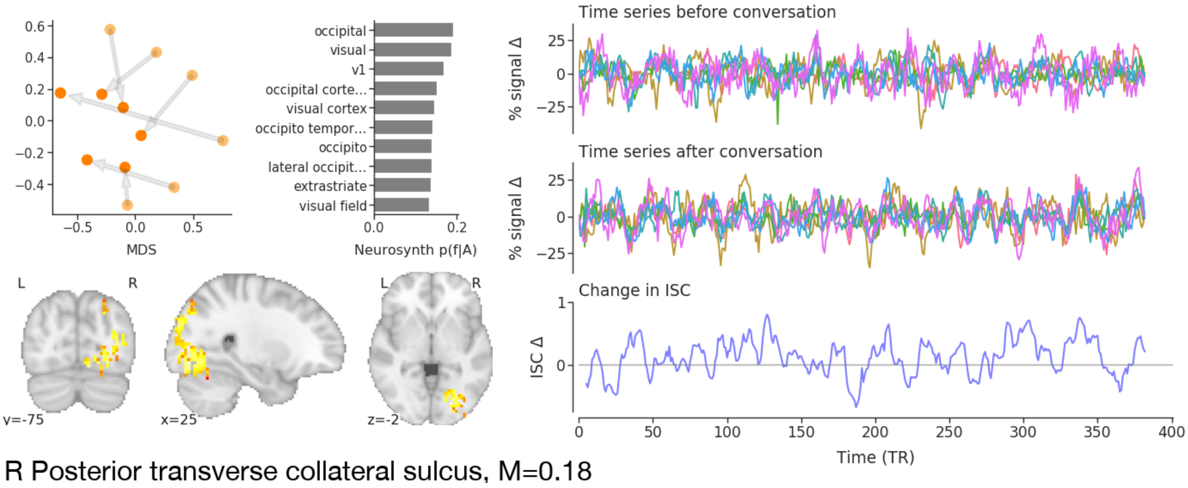


**Supplementary Figure 47:** Change in ISC. Movie clip: Sexy Beast. Group: E

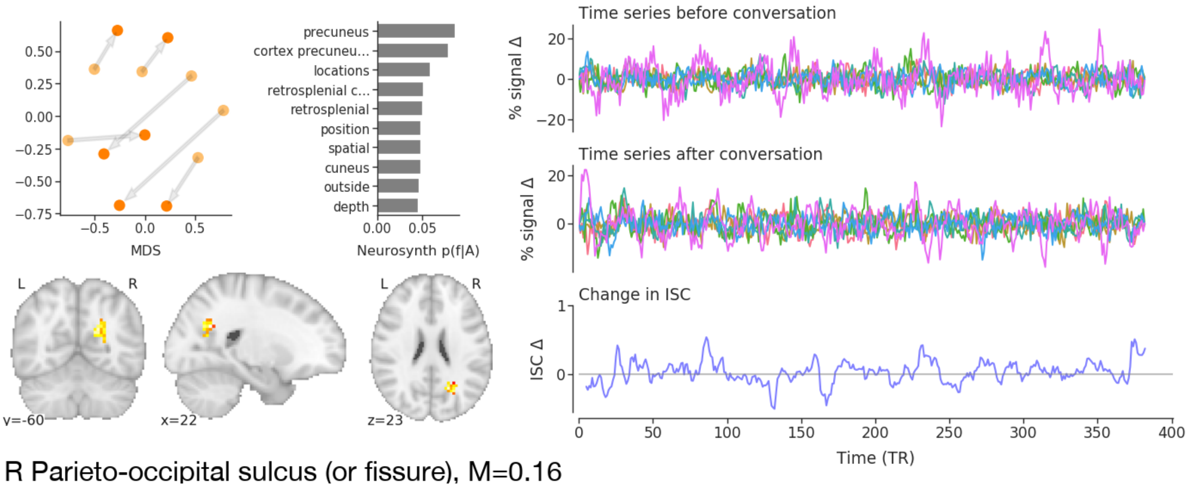


**Supplementary Figure 48:** Change in ISC. Movie clip: Sexy Beast. Group: E

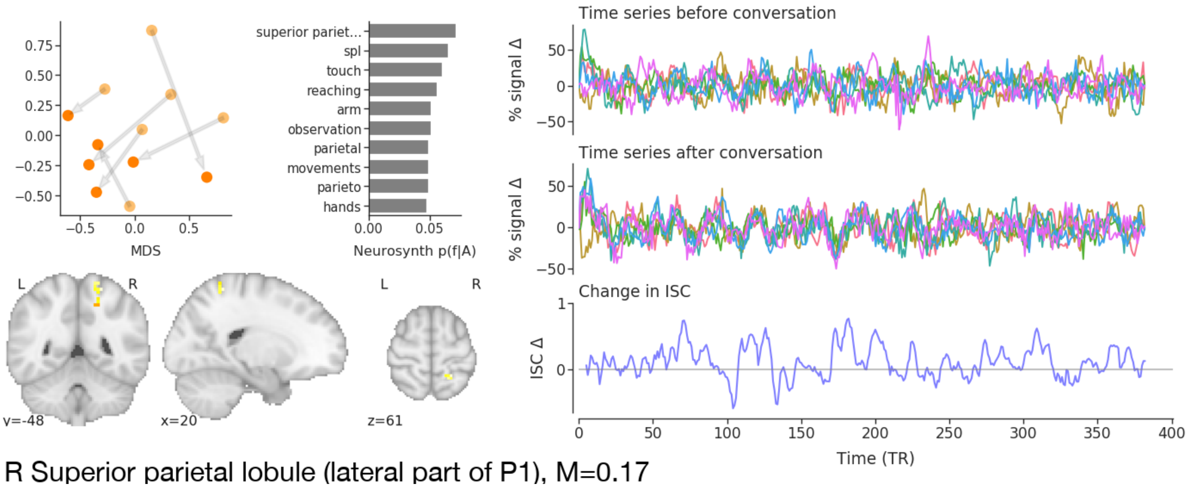


**Supplementary Figure 49:** Change in ISC. Movie clip: Sexy Beast. Group: E

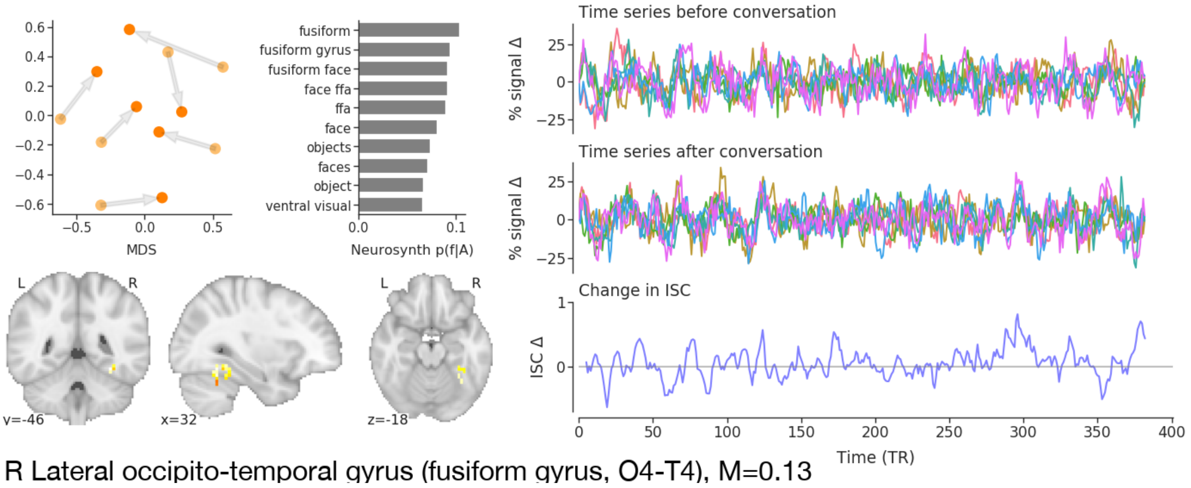


**Supplementary Figure 50:** Change in ISC. Movie clip: Sexy Beast. Group: E

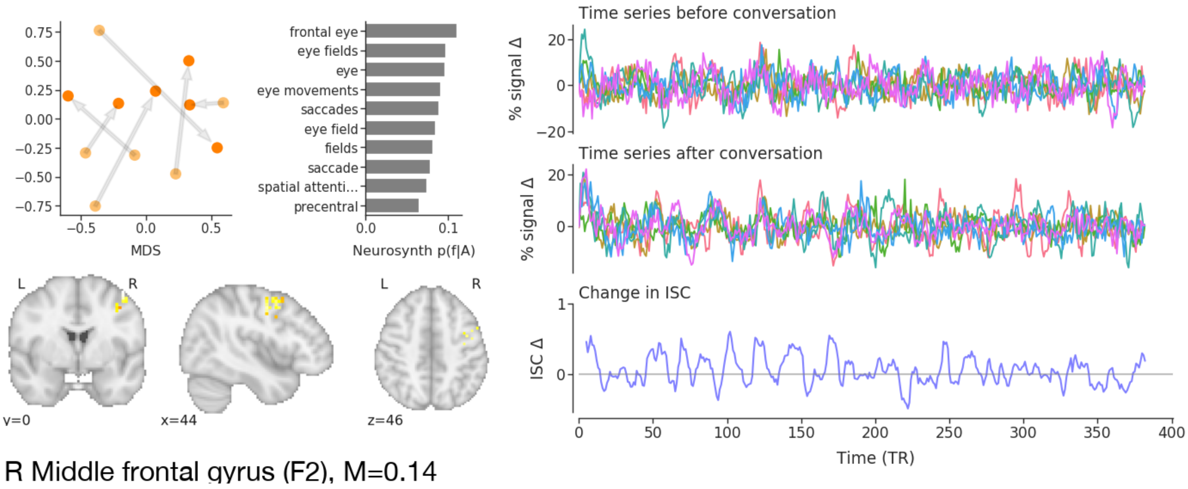


**Supplementary Figure 51:** Change in ISC. Movie clip: Sexy Beast. Group: E

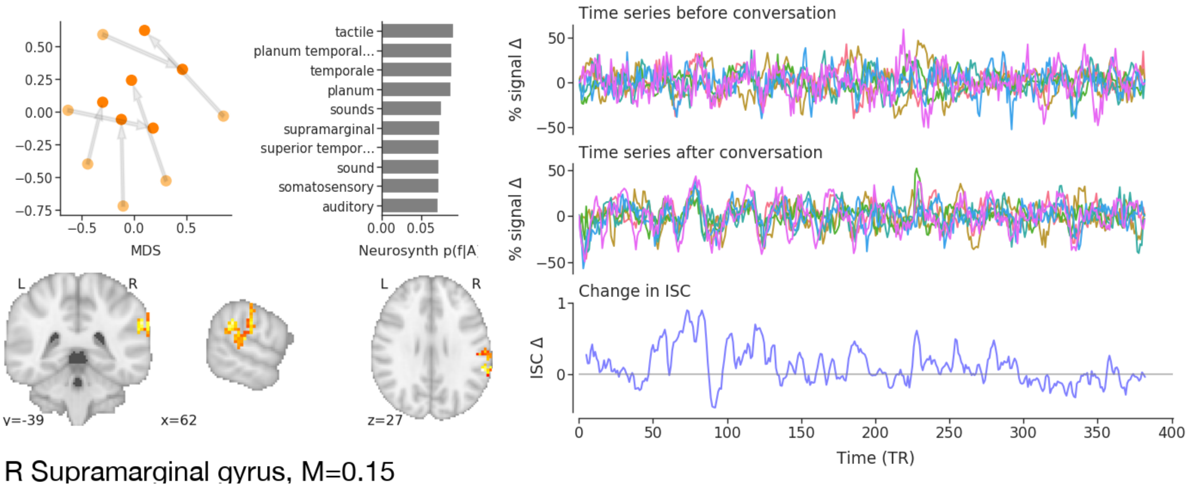


**Supplementary Figure 52:** Change in ISC. Movie clip: Sexy Beast. Group: E

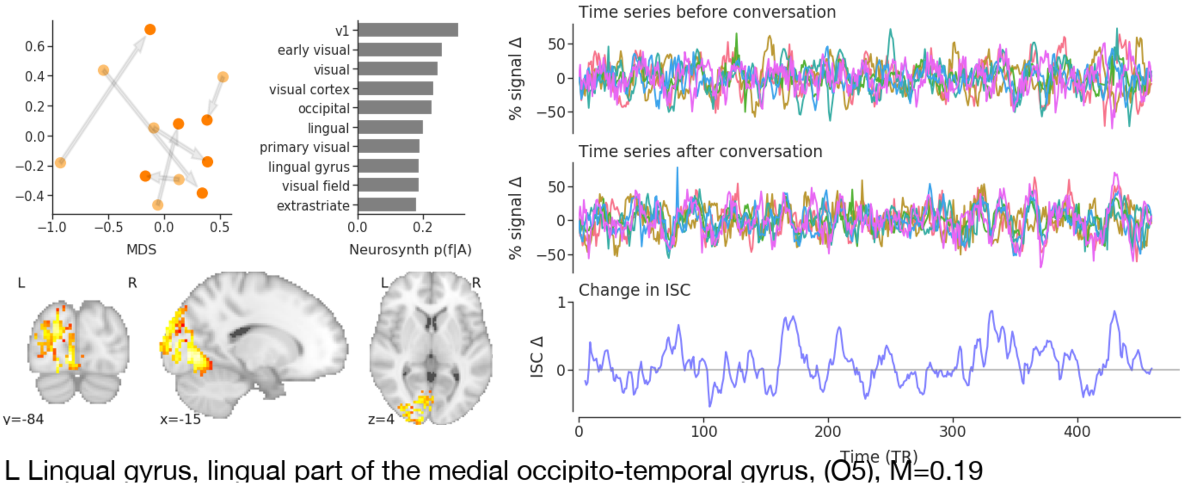


**Supplementary Figure 53:** Change in ISC. Movie clip: The Master. Group: E

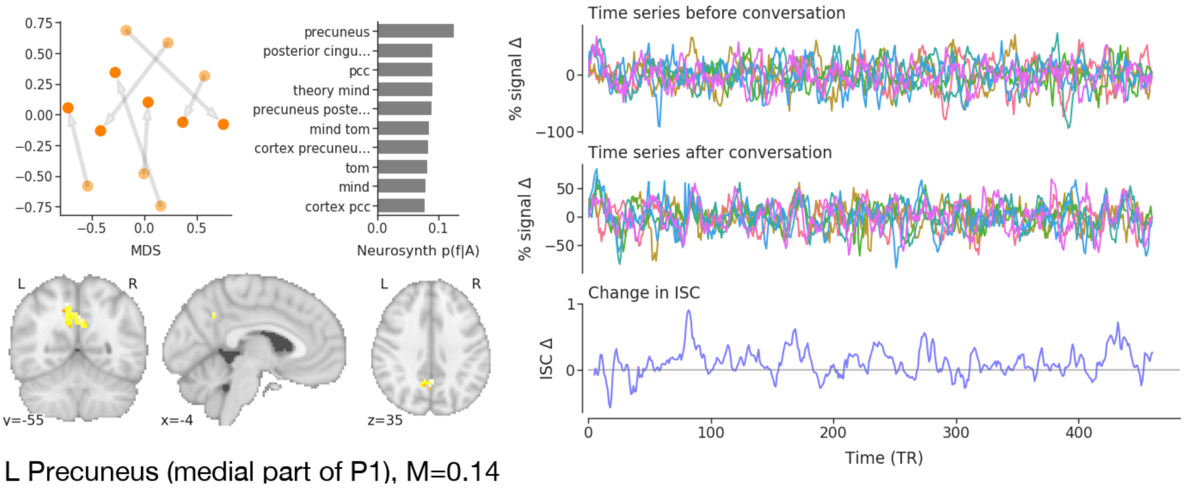


**Supplementary Figure 54:** Change in ISC. Movie clip: The Master. Group: E

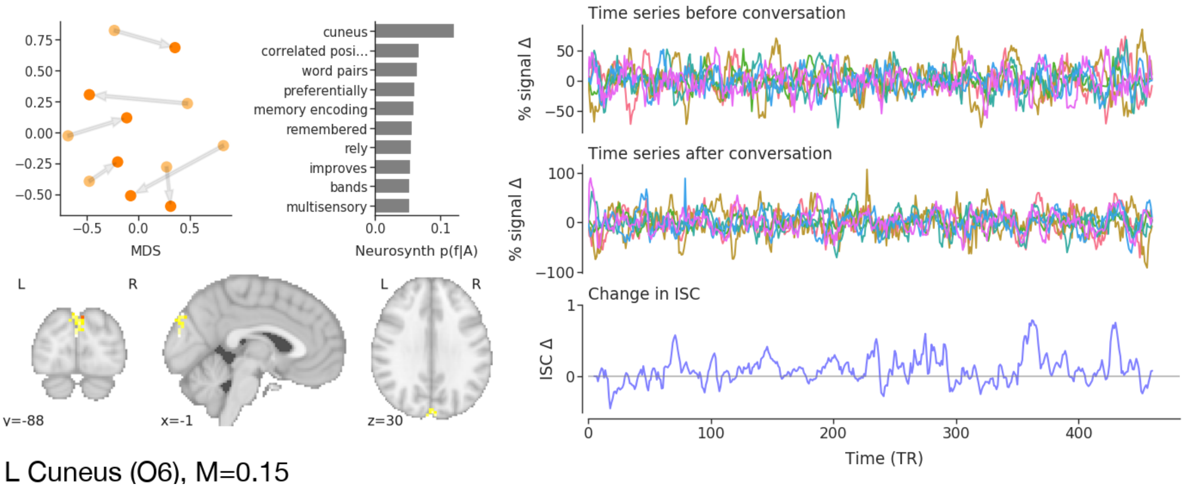


**Supplementary Figure 55:** Change in ISC. Movie clip: The Master. Group: E

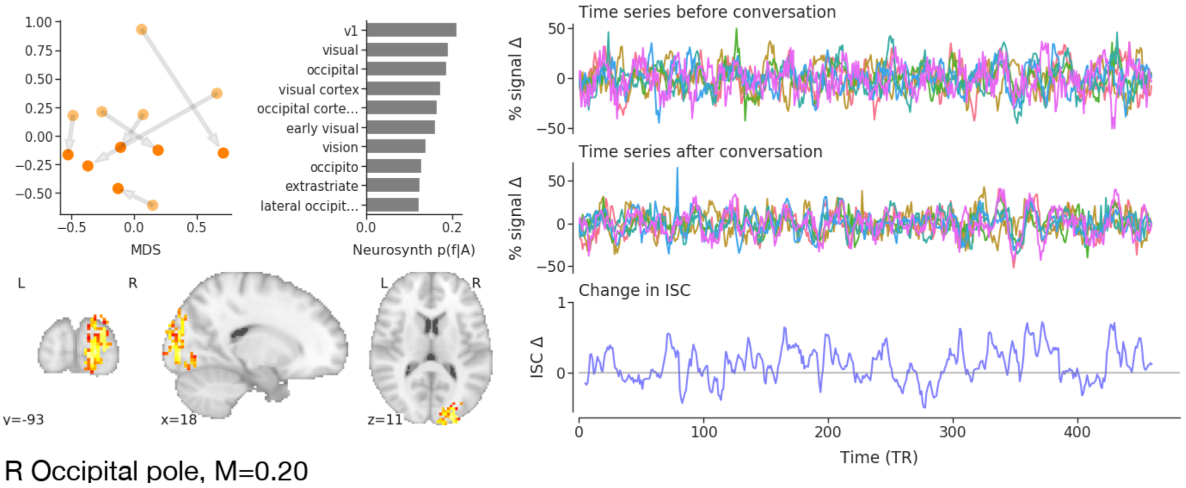


**Supplementary Figure 56:** Change in ISC. Movie clip: The Master. Group: E

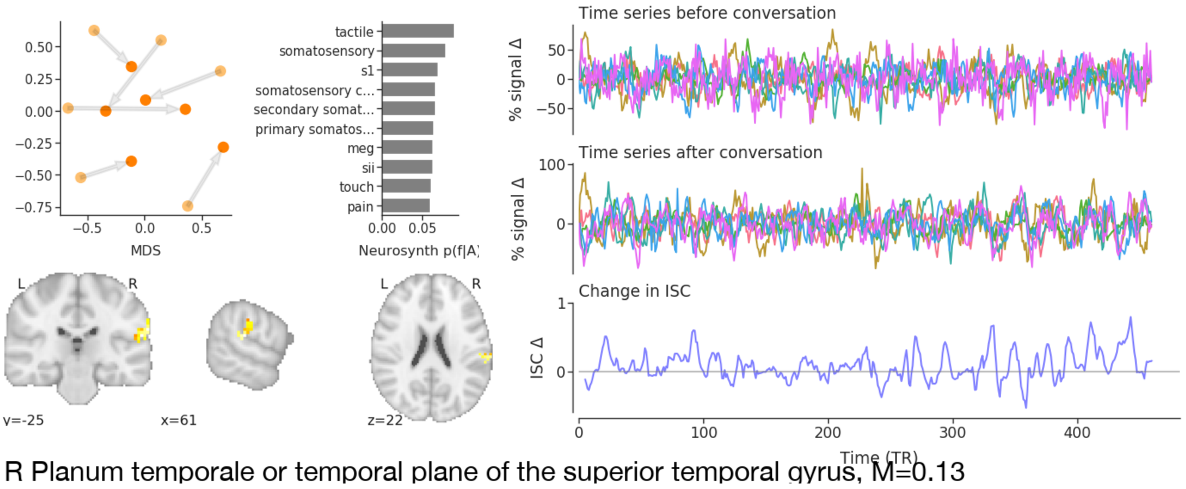


**Supplementary Figure 57:** Change in ISC. Movie clip: The Master. Group: E

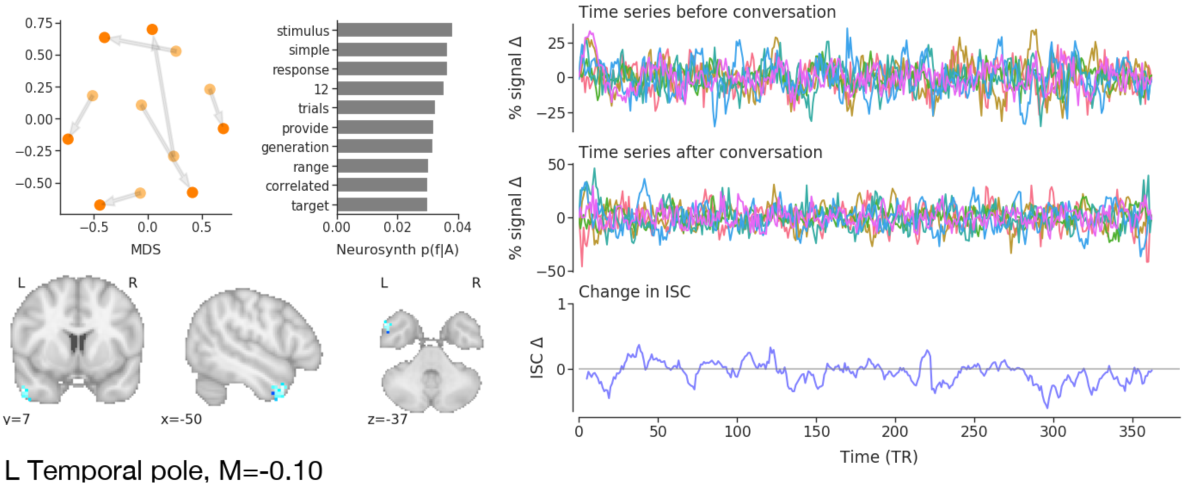


**Supplementary Figure 58:** Change in ISC. Movie clip: Y tu mamá también. Group: E

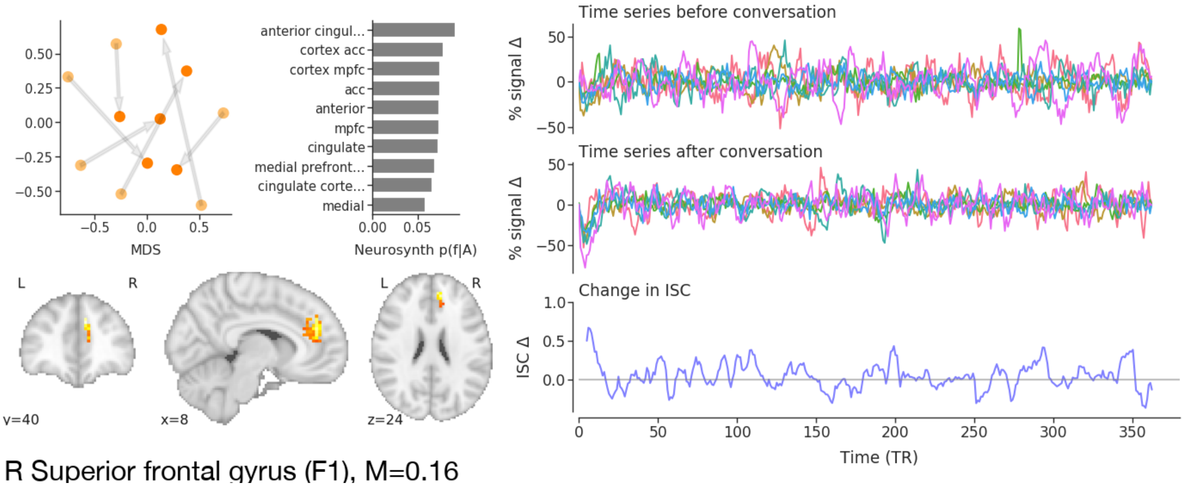


**Supplementary Figure 59:** Change in ISC. Movie clip: Y tu mamá también. Group: E

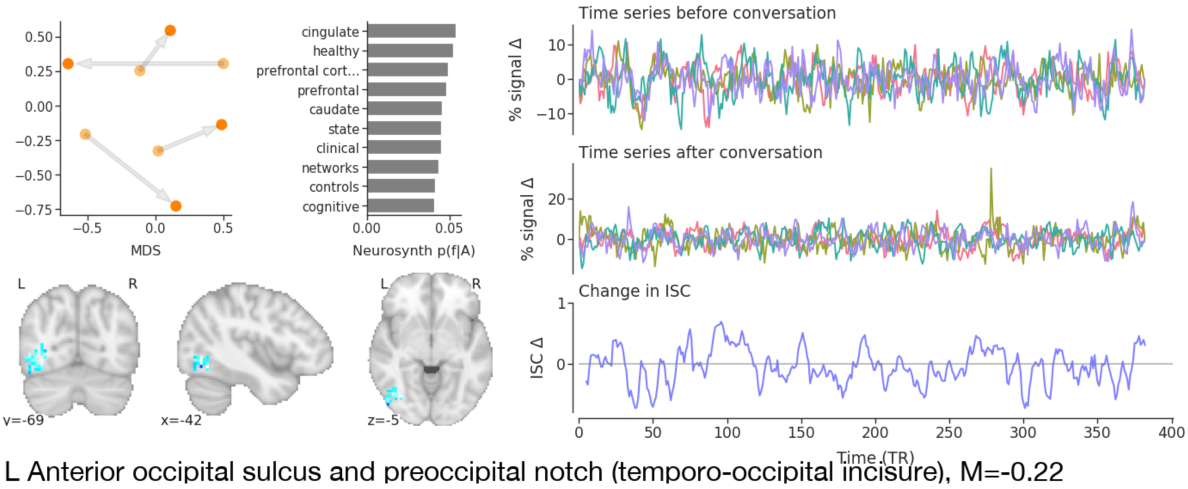


**Supplementary Figure 60:** Change in ISC. Movie clip: Sexy Beast. Group: F

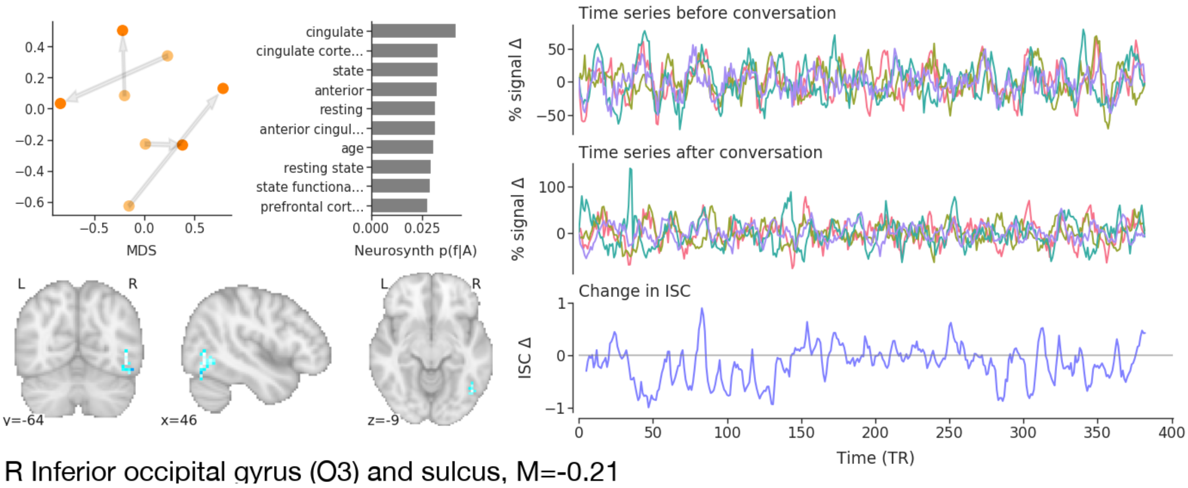


**Supplementary Figure 61:** Change in ISC. Movie clip: Sexy Beast. Group: F

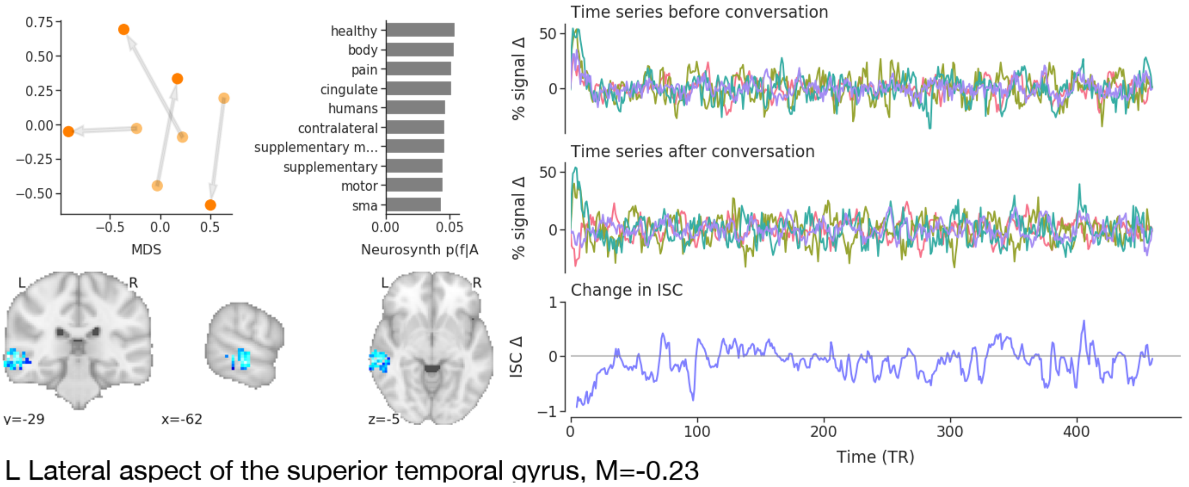


**Supplementary Figure 62:** Change in ISC. Movie clip: The Master. Group: F

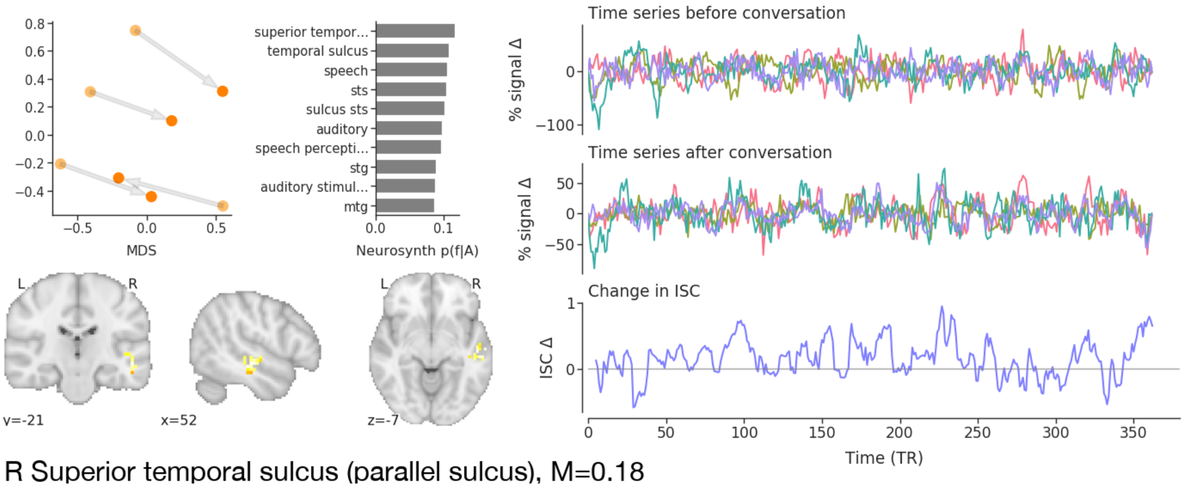


**Supplementary Figure 63:** Change in ISC. Movie clip: Y tu mamá también. Group: F

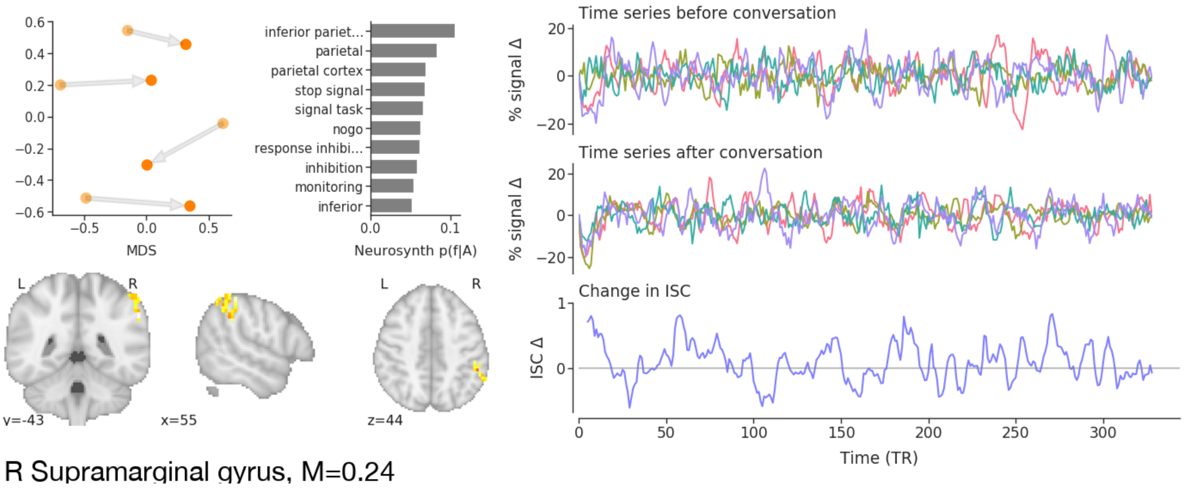


**Supplementary Figure 64:** Change in ISC. Movie clip: Birth. Group: G

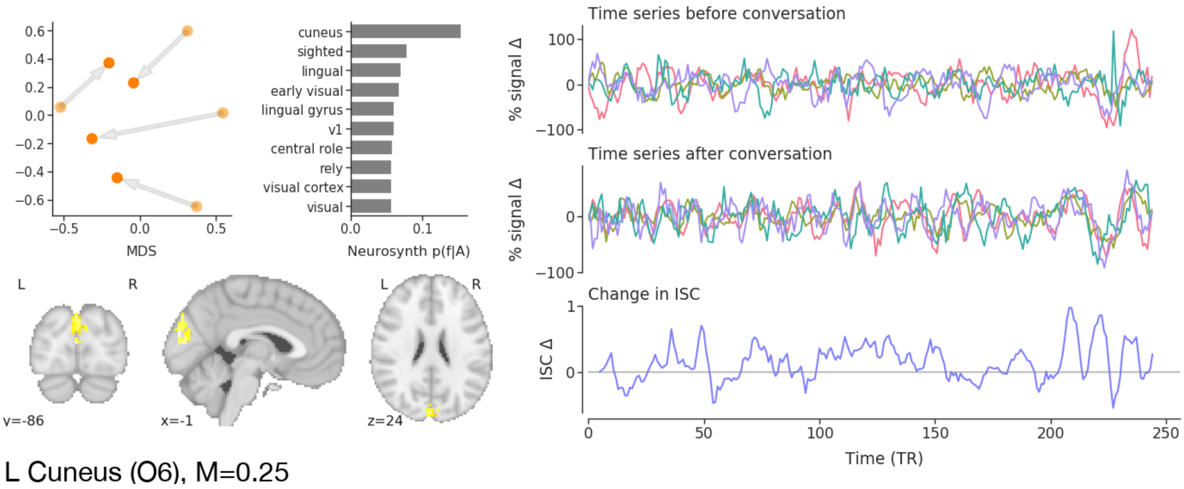


**Supplementary Figure 65:** Change in ISC. Movie clip: The Assassination of Jesse James by the Coward Robert Ford. Group: G

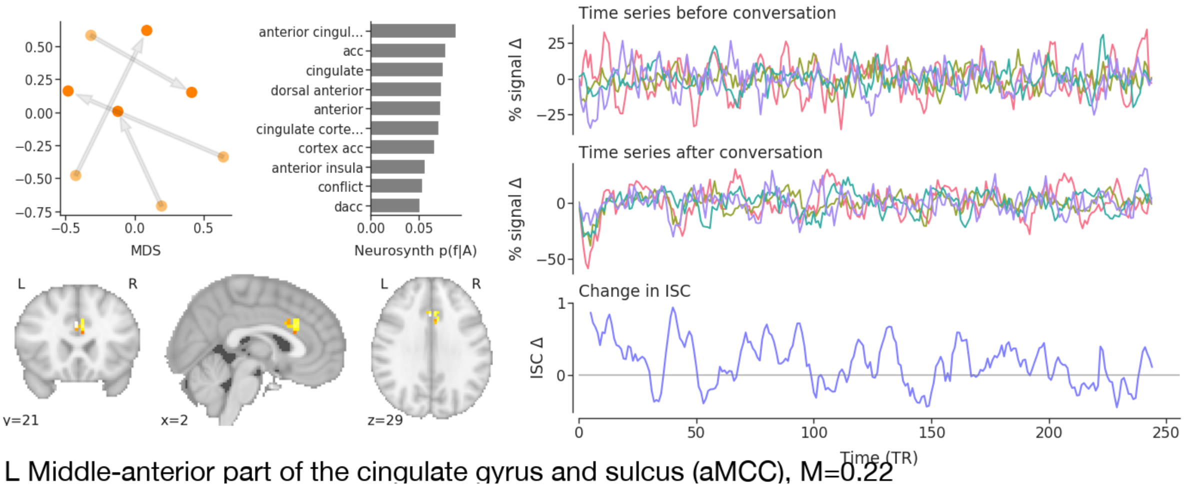


**Supplementary Figure 66:** Change in ISC. Movie clip: The Assassination of Jesse James by the Coward Robert Ford. Group: G

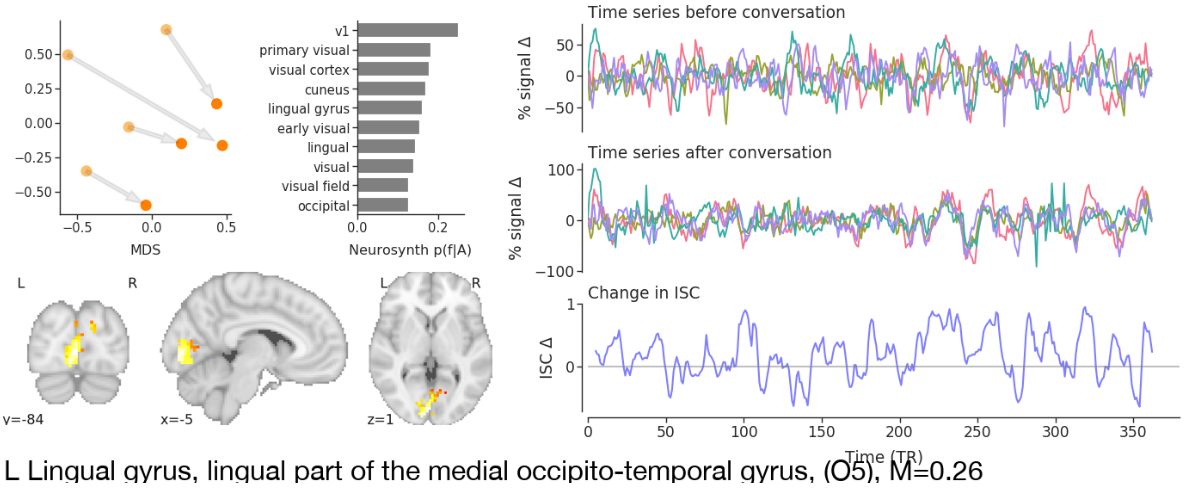


**Supplementary Figure 67:** Change in ISC. Movie clip: Y tu mamá también. Group: G

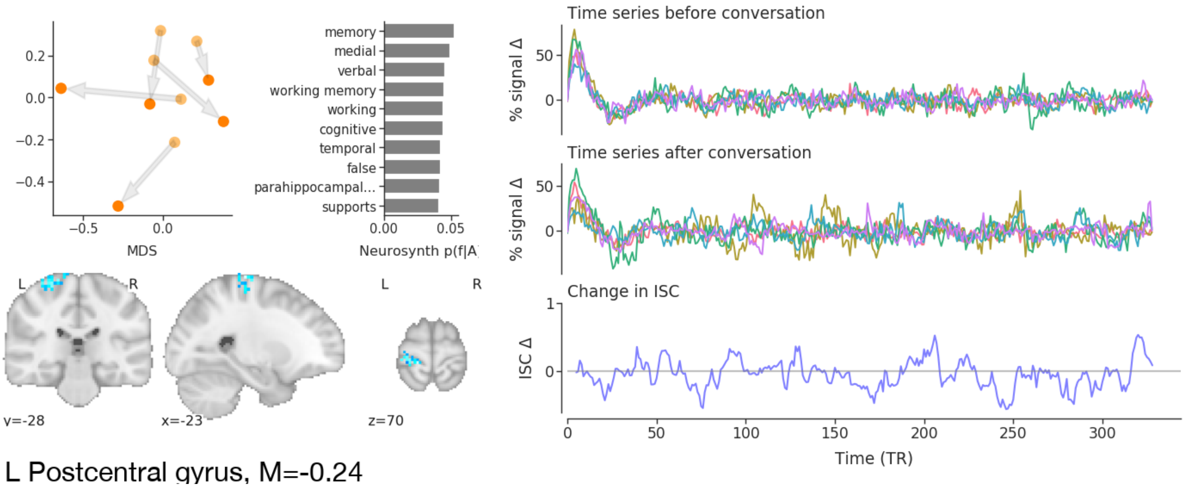


**Supplementary Figure 68:** Change in ISC. Movie clip: Birth. Group: H

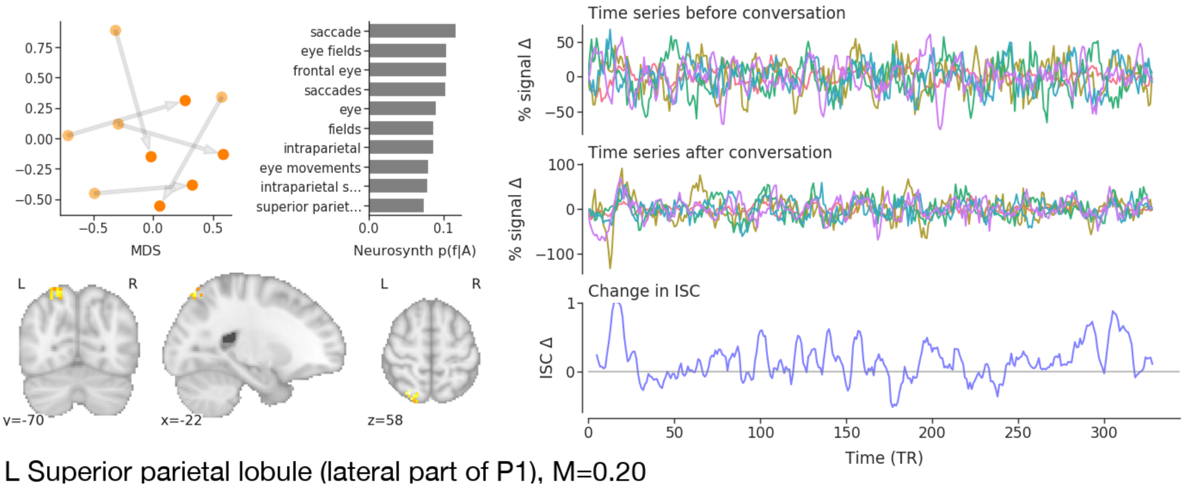


**Supplementary Figure 69:** Change in ISC. Movie clip: Birth. Group: H

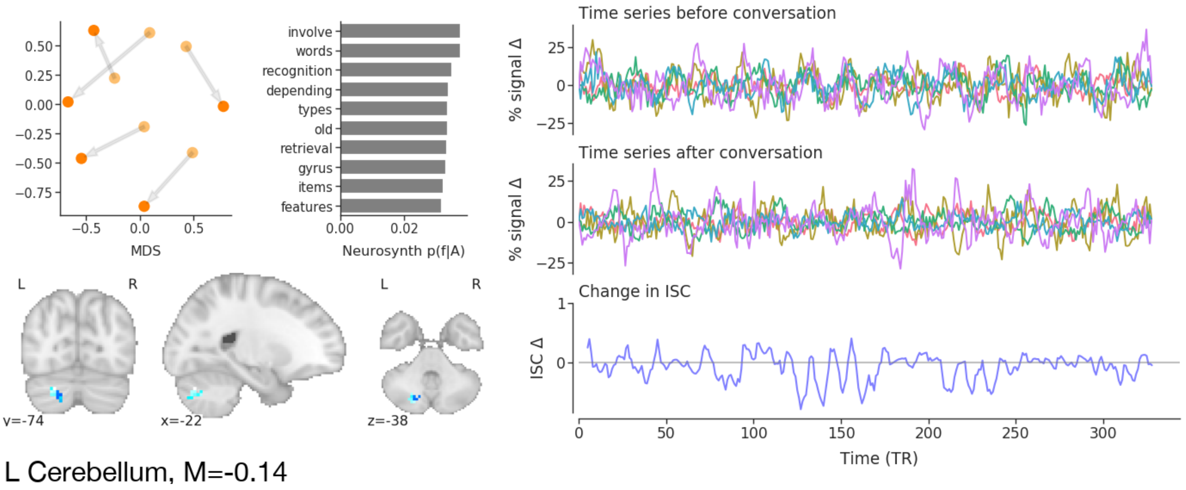


**Supplementary Figure 70:** Change in ISC. Movie clip: Birth. Group: H

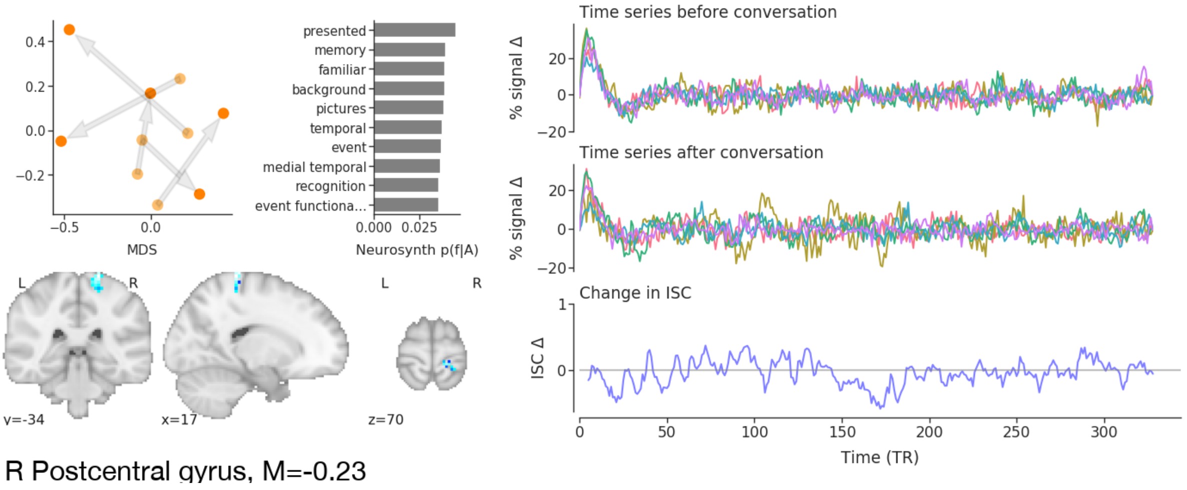


**Supplementary Figure 71:** Change in ISC. Movie clip: Birth. Group: H

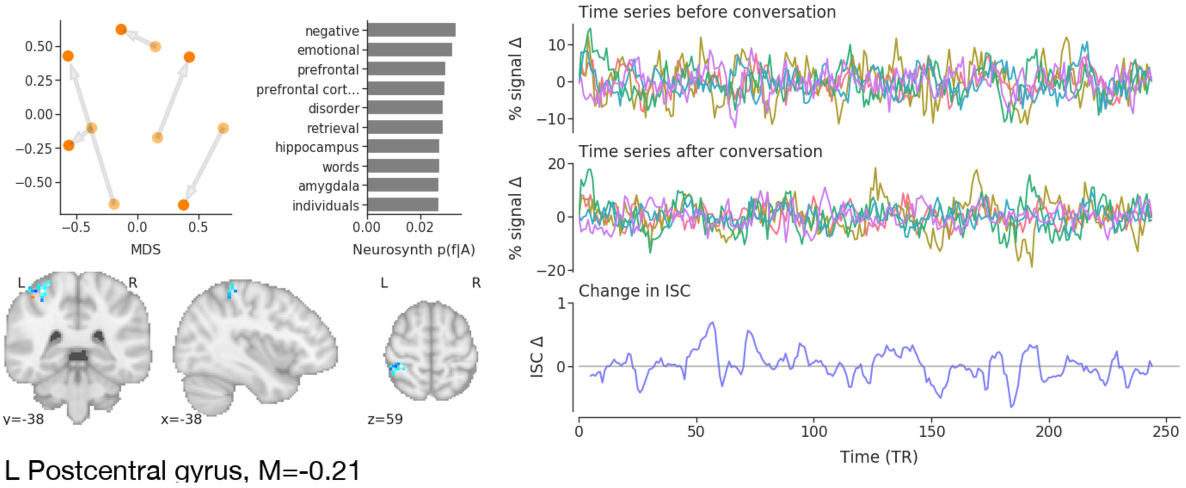


**Supplementary Figure 72:** Change in ISC. Movie clip: The Assassination of Jesse James by the Coward Robert Ford. Group: H

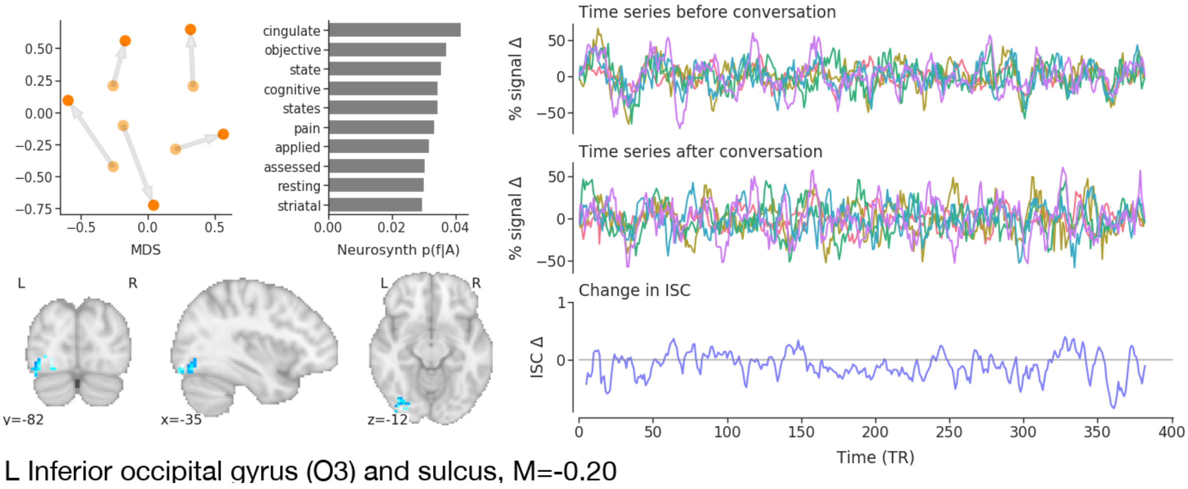


**Supplementary Figure 73:** Change in ISC. Movie clip: Sexy Beast. Group: H

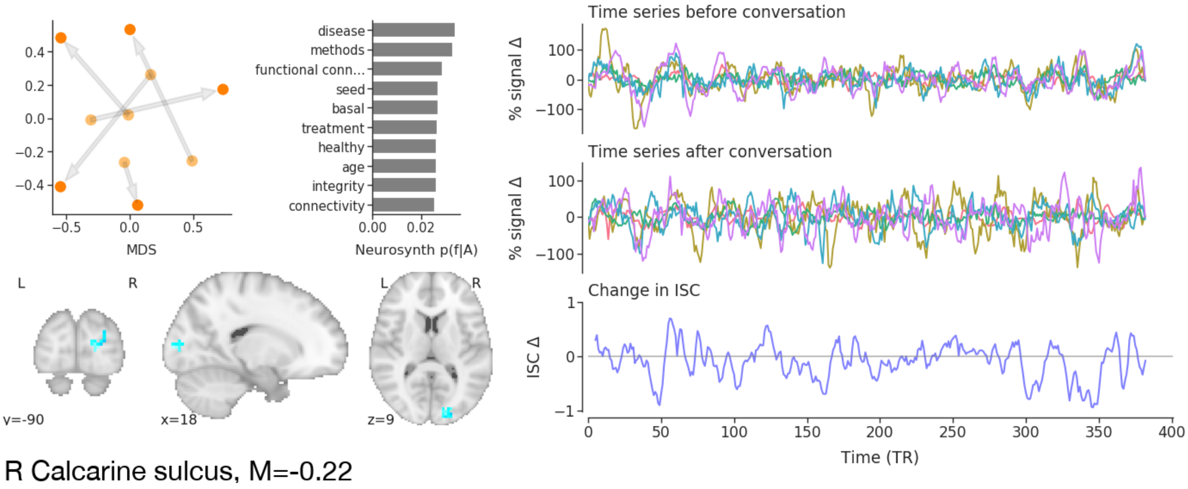


**Supplementary Figure 74:** Change in ISC. Movie clip: Sexy Beast. Group: H

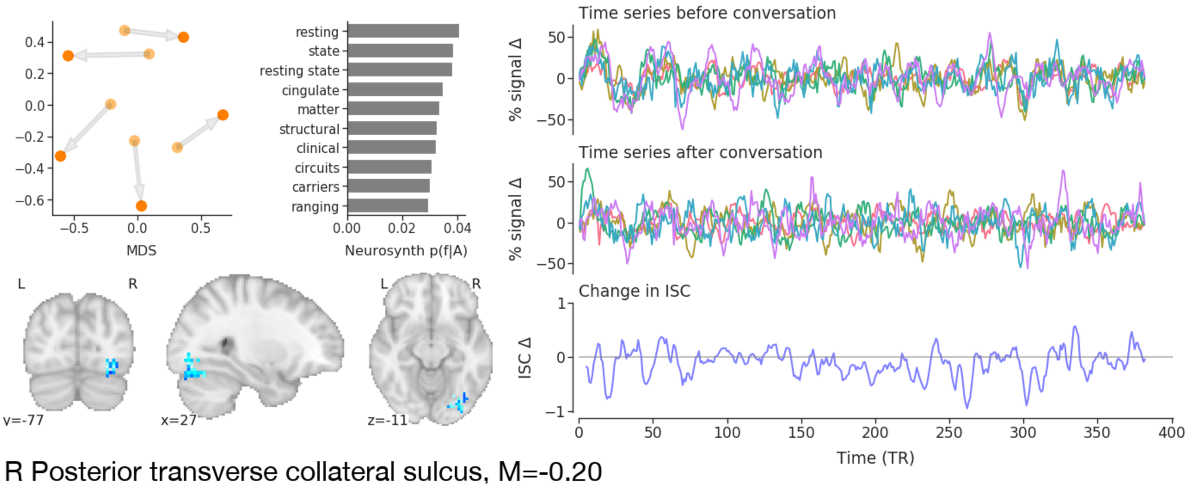


**Supplementary Figure 75:** Change in ISC. Movie clip: Sexy Beast. Group: H

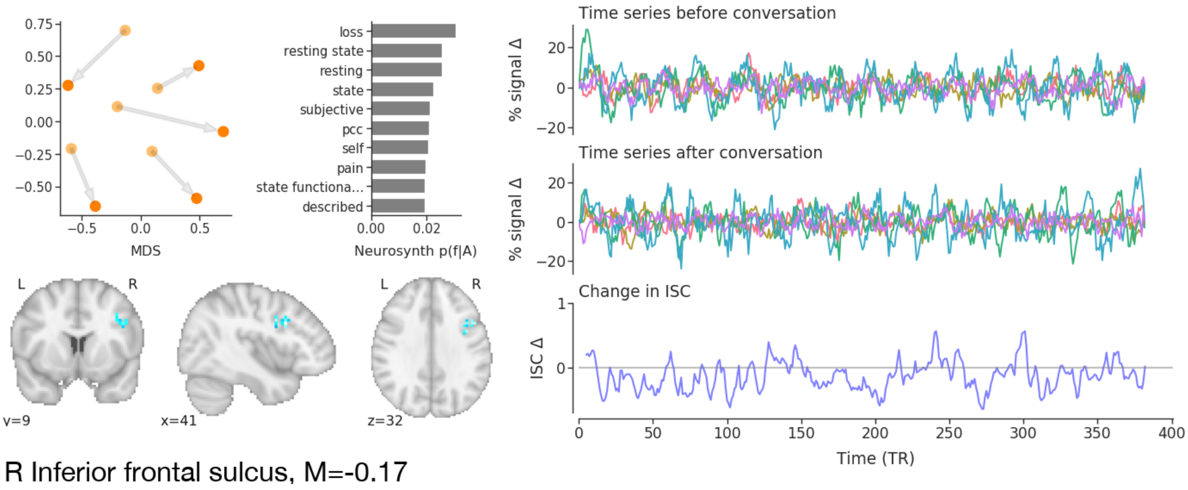


**Supplementary Figure 76:** Change in ISC. Movie clip: Sexy Beast. Group: H

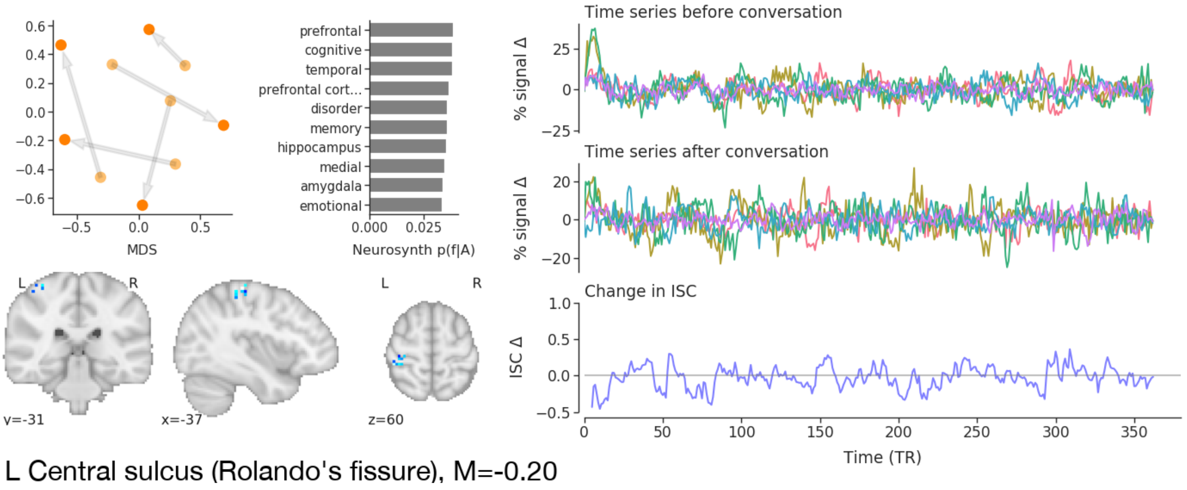


**Supplementary Figure 77:** Change in ISC. Movie clip: Y tu mamá también. Group: H

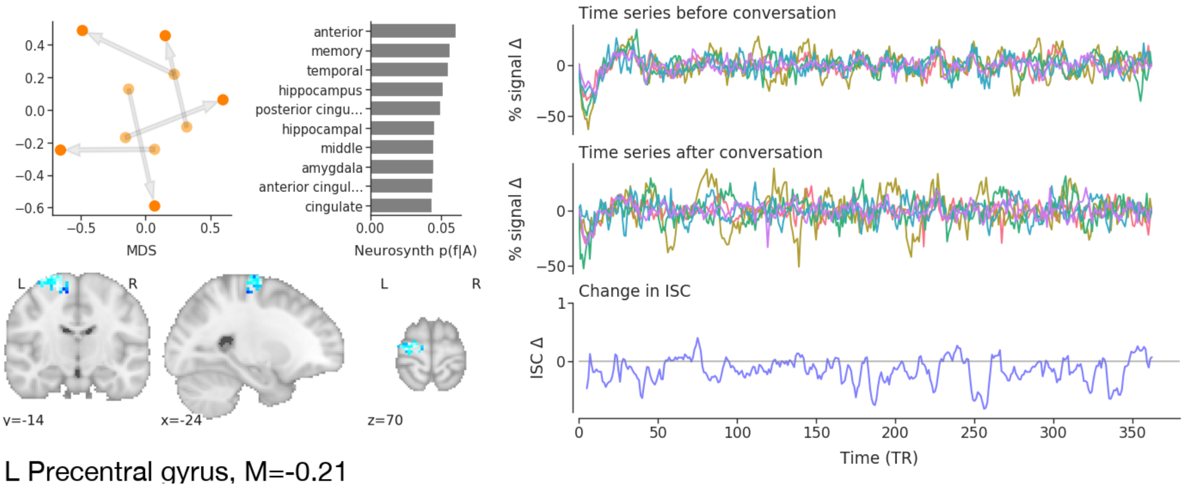


**Supplementary Figure 78:** Change in ISC. Movie clip: Y tu mamá también. Group: H

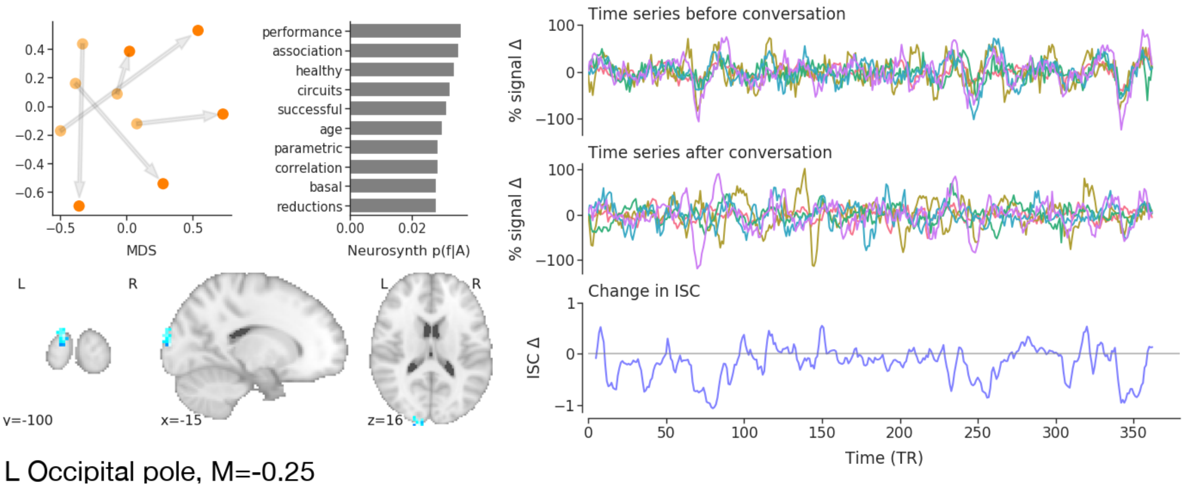


**Supplementary Figure 79:** Change in ISC. Movie clip: Y tu mamá también. Group: H

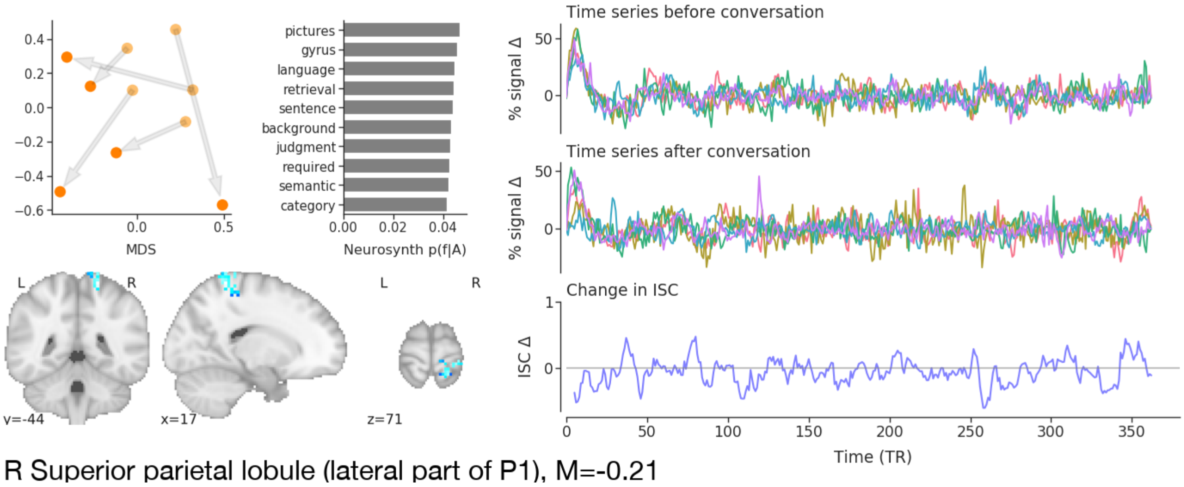


**Supplementary Figure 80:** Change in ISC. Movie clip: Y tu mamá también. Group: H

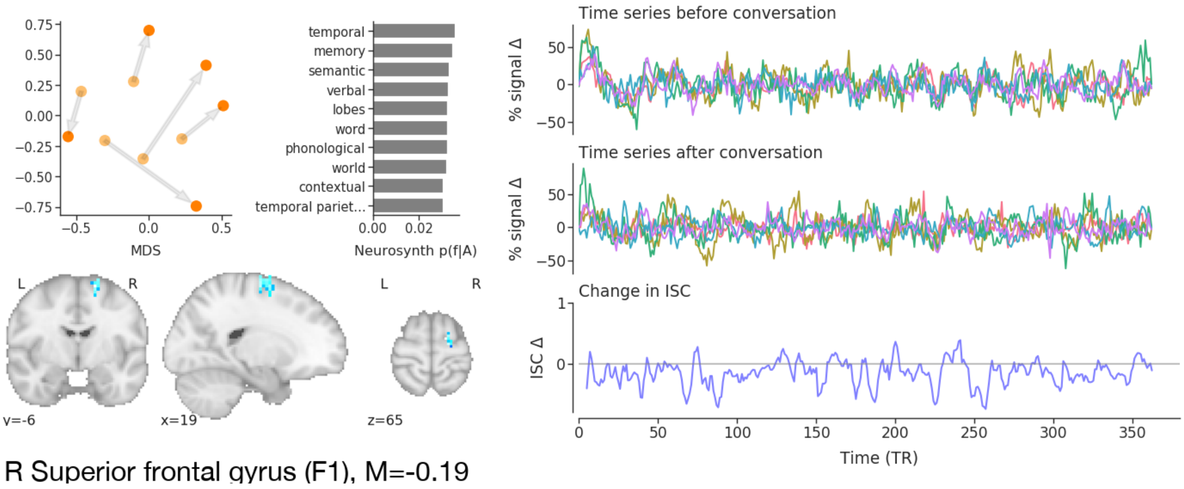


**Supplementary Figure 81:** Change in ISC. Movie clip: Y tu mamá también. Group: H

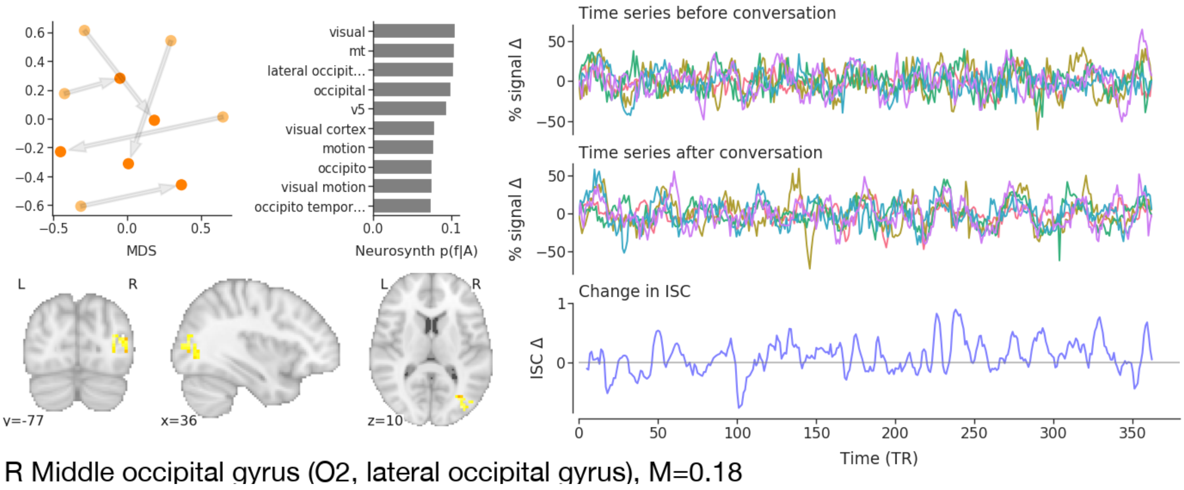


**Supplementary Figure 82:** Change in ISC. Movie clip: Y tu mamá también. Group: H

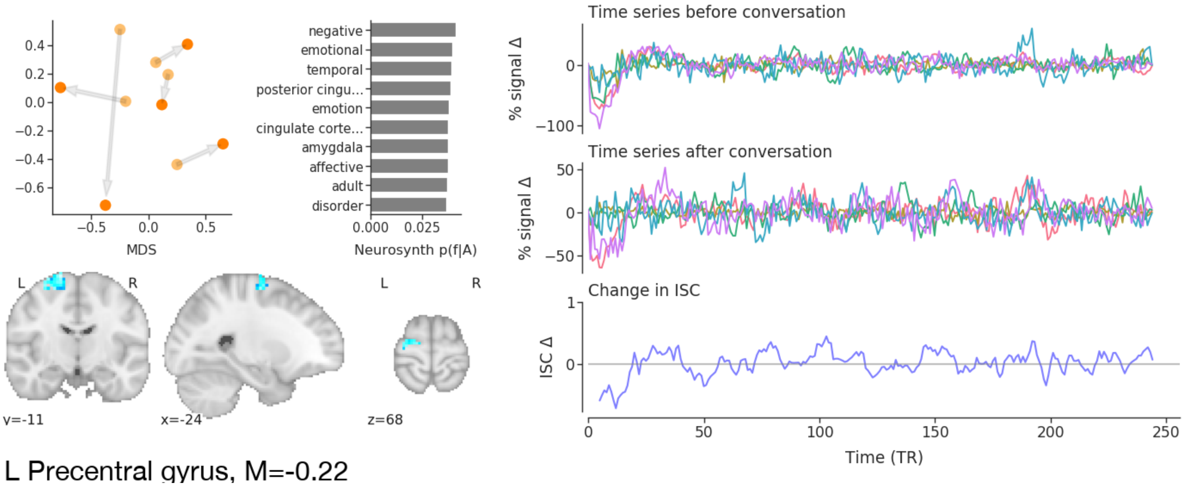


**Supplementary Figure 83:** Change in ISC. Movie clip: The Assassination of Jesse James by the Coward Robert Ford. Group: I

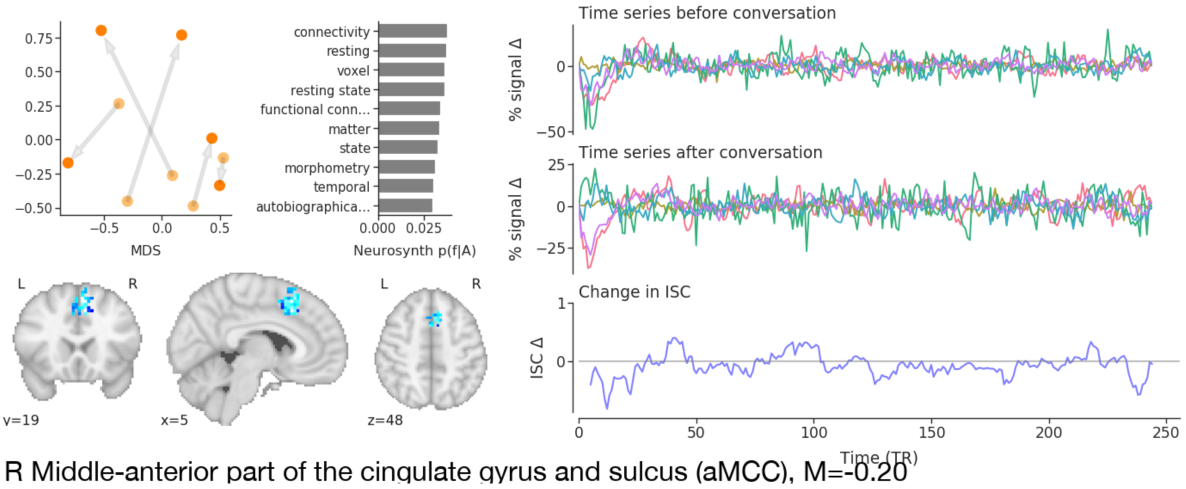


**Supplementary Figure 84:** Change in ISC. Movie clip: The Assassination of Jesse James by the Coward Robert Ford. Group: I

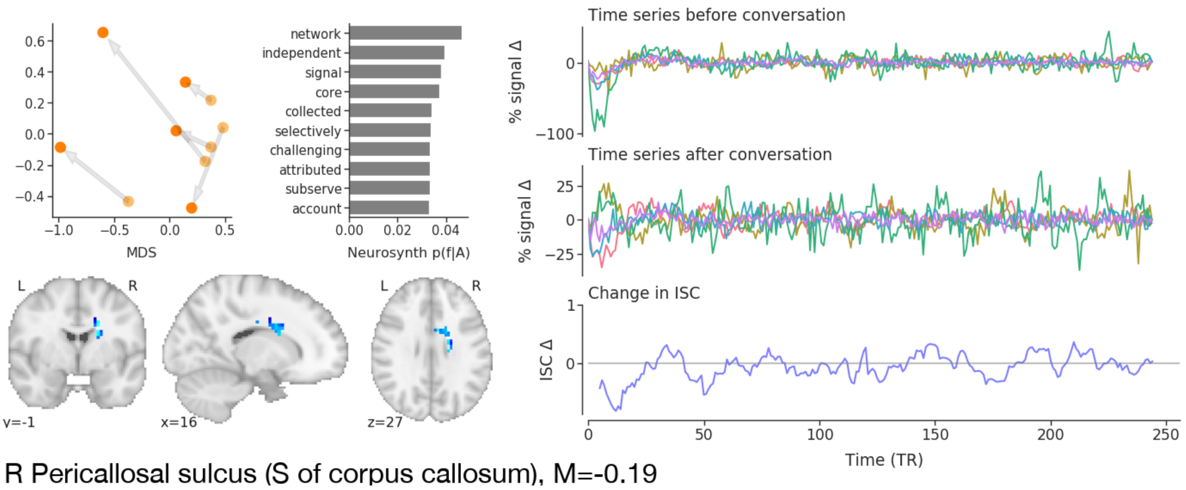


**Supplementary Figure 85:** Change in ISC. Movie clip: The Assassination of Jesse James by the Coward Robert Ford. Group: I

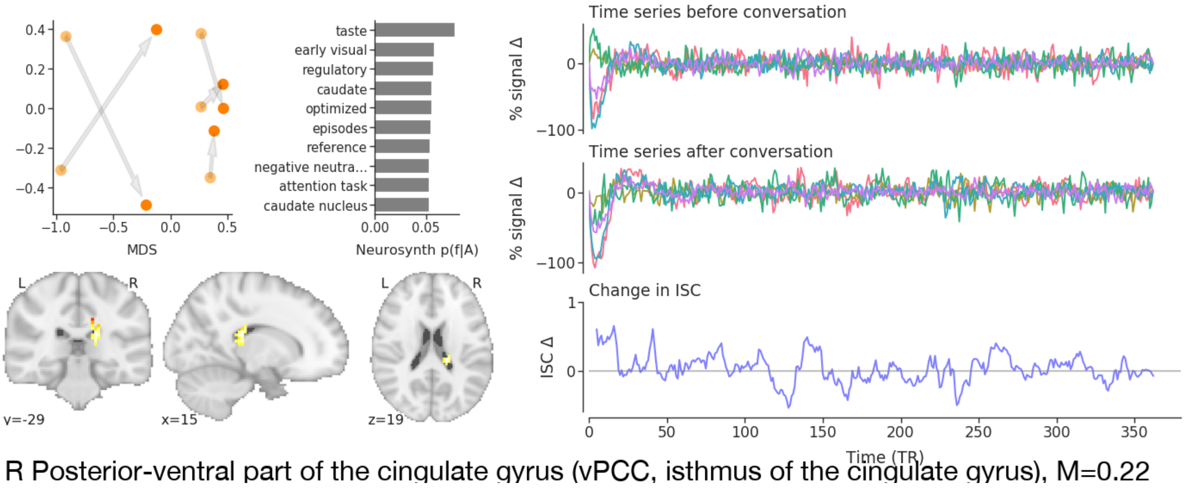


**Supplementary Figure 86:** Change in ISC. Movie clip: Y tu mamá también. Group: I

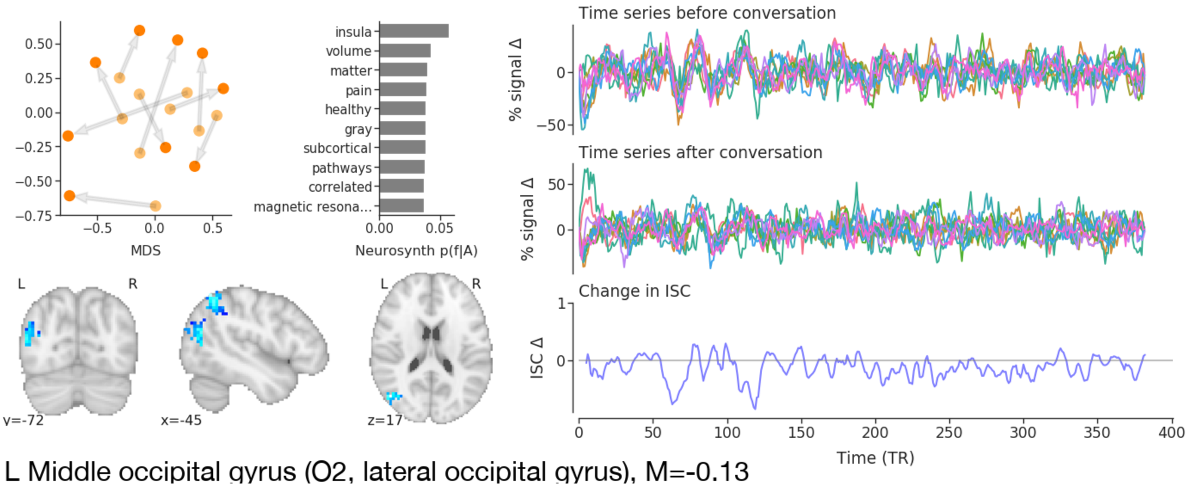


**Supplementary Figure 87:** Change in ISC. Movie clip: Sexy Beast. Group: Control

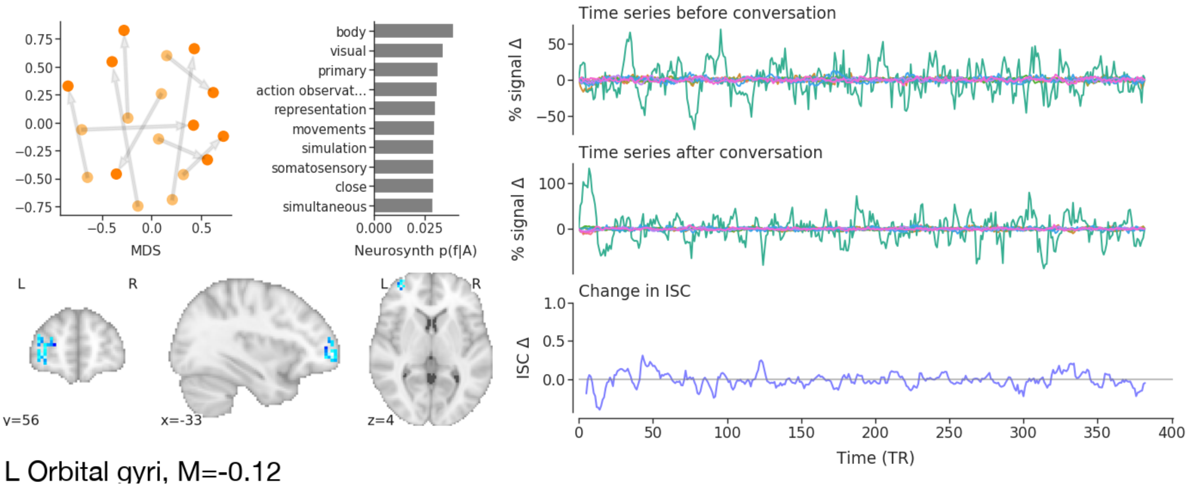


**Supplementary Figure 88:** Change in ISC. Movie clip: Sexy Beast. Group: Control

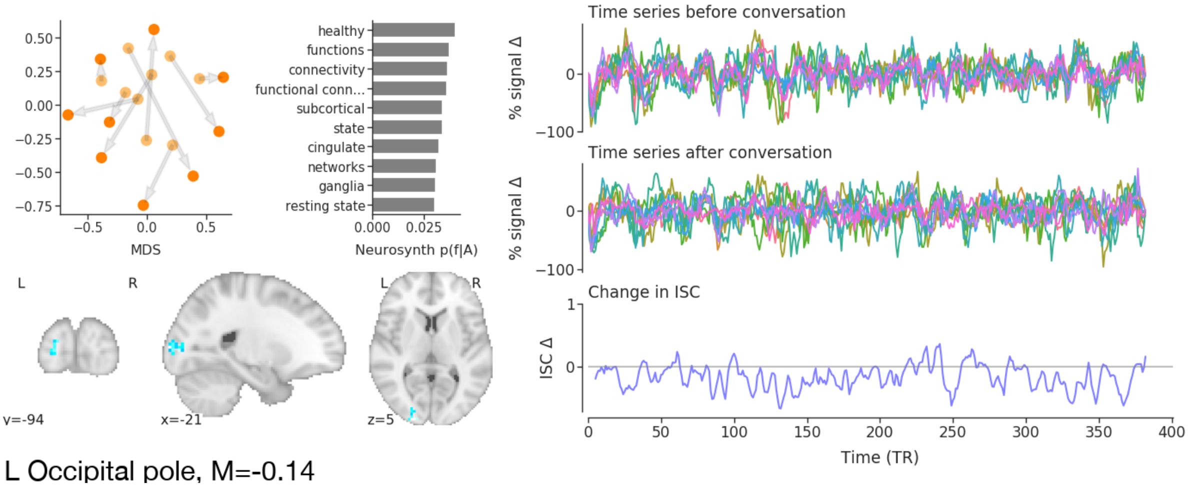


**Supplementary Figure 89:** Change in ISC. Movie clip: Sexy Beast. Group: Control

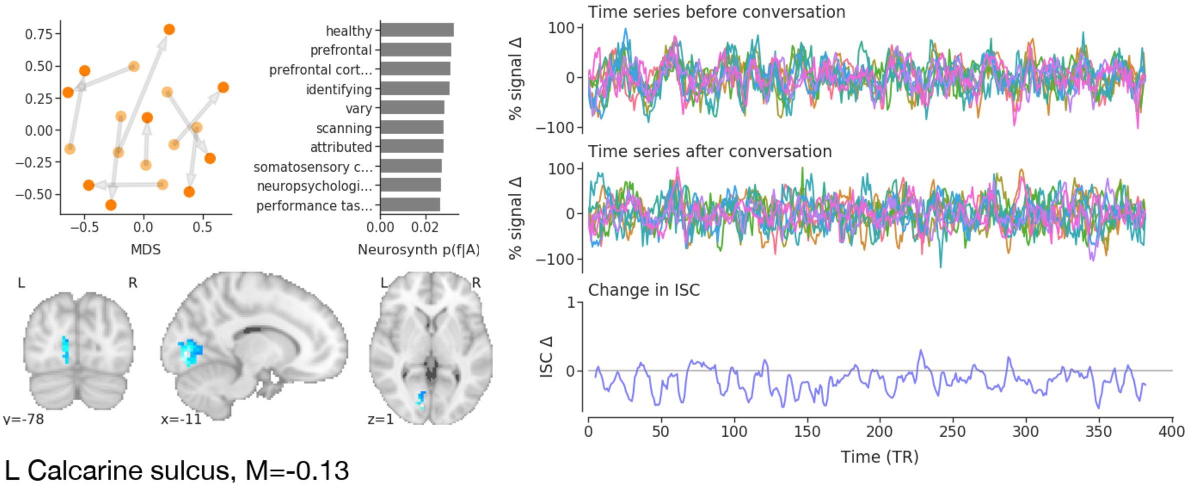


**Supplementary Figure 90:** Change in ISC. Movie clip: Sexy Beast. Group: Control

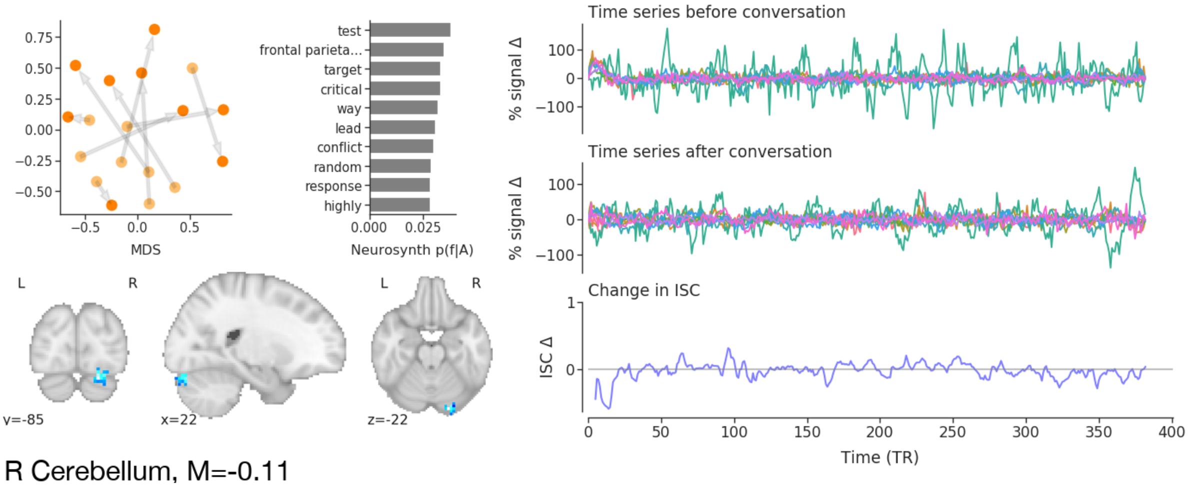


**Supplementary Figure 91:** Change in ISC. Movie clip: Sexy Beast. Group: Control

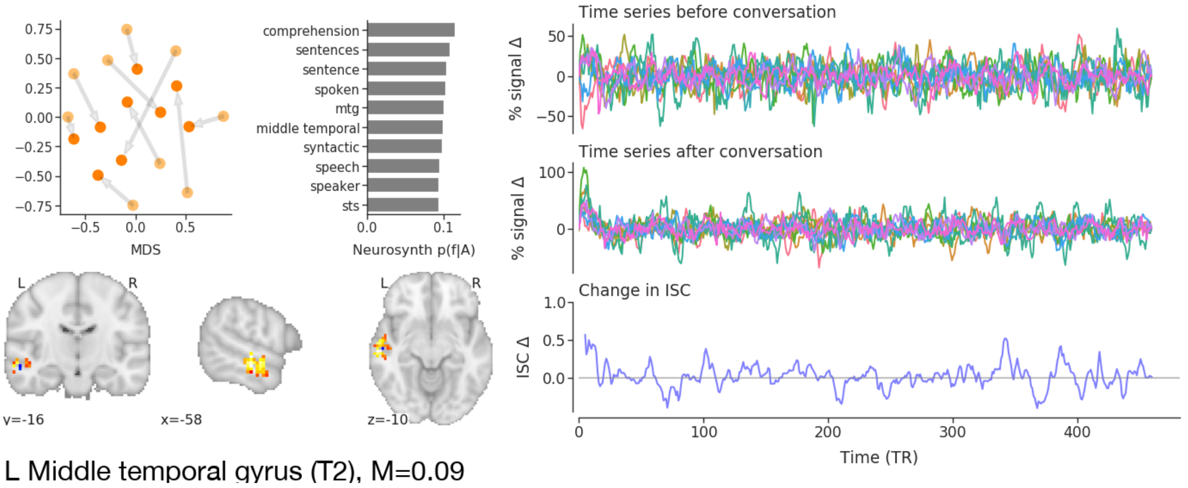


**Supplementary Figure 92:** Change in ISC. Movie clip: The Master. Group: Control

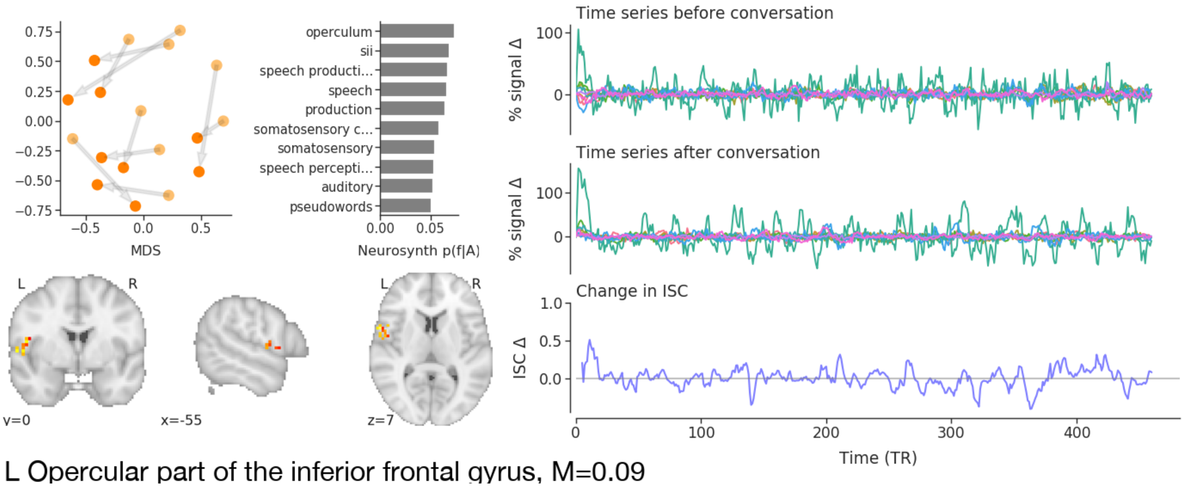


**Supplementary Figure 93:** Change in ISC. Movie clip: The Master. Group: Control

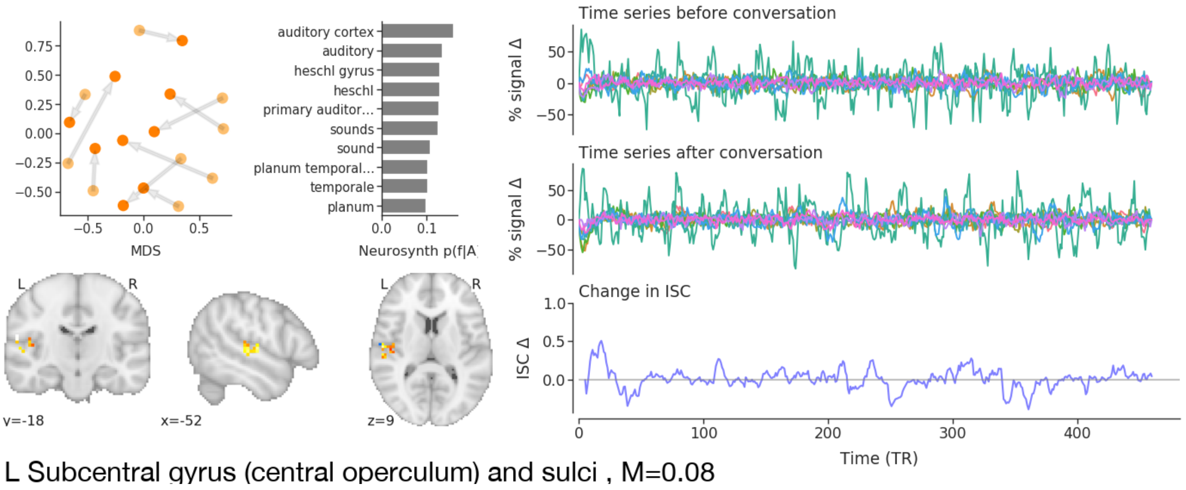


**Supplementary Figure 94:** Change in ISC. Movie clip: The Master. Group: Control

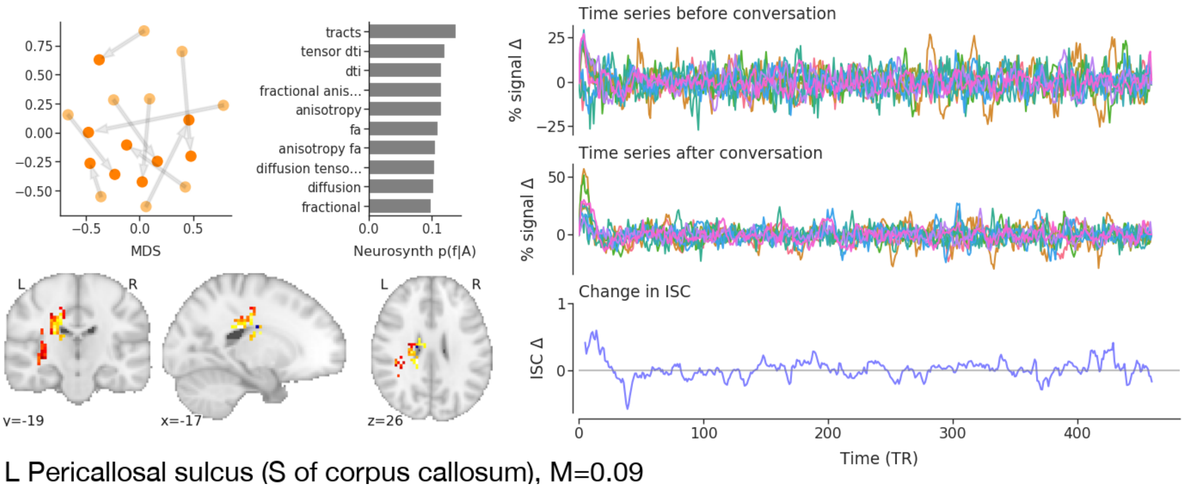


**Supplementary Figure 95:** Change in ISC. Movie clip: The Master. Group: Control

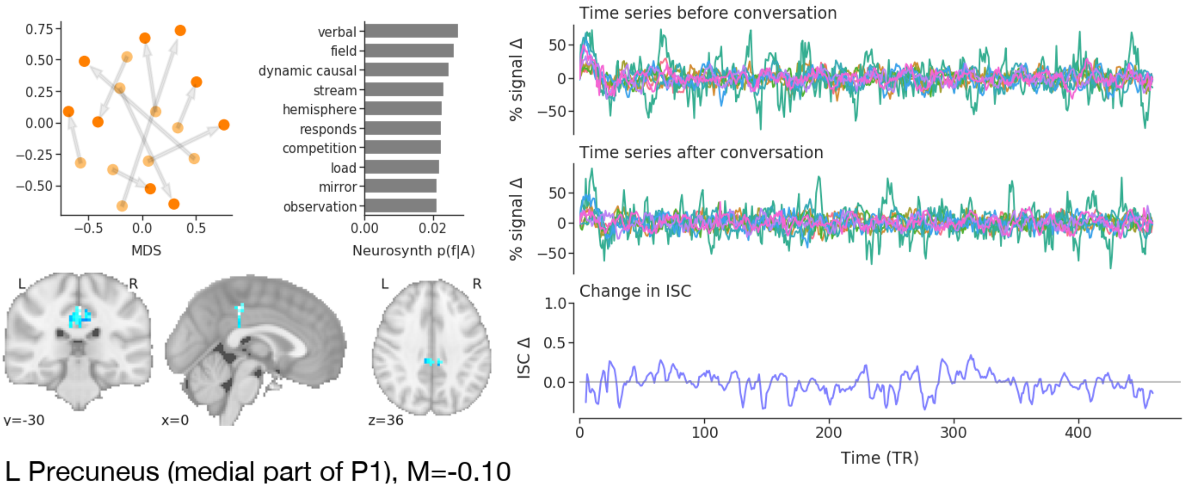


**Supplementary Figure 96:** Change in ISC. Movie clip: The Master. Group: Control

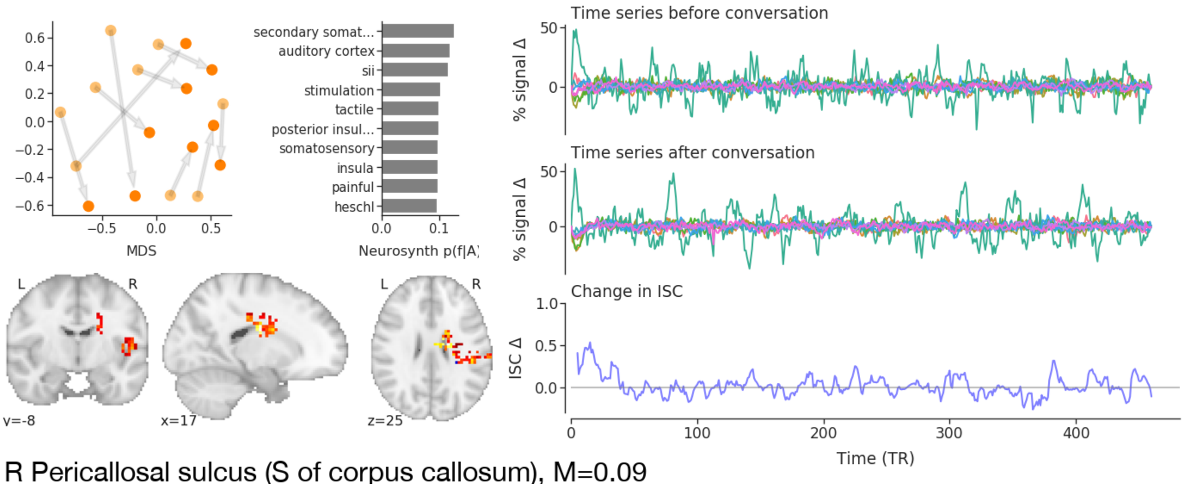


**Supplementary Figure 97:** Change in ISC. Movie clip: The Master. Group: Control

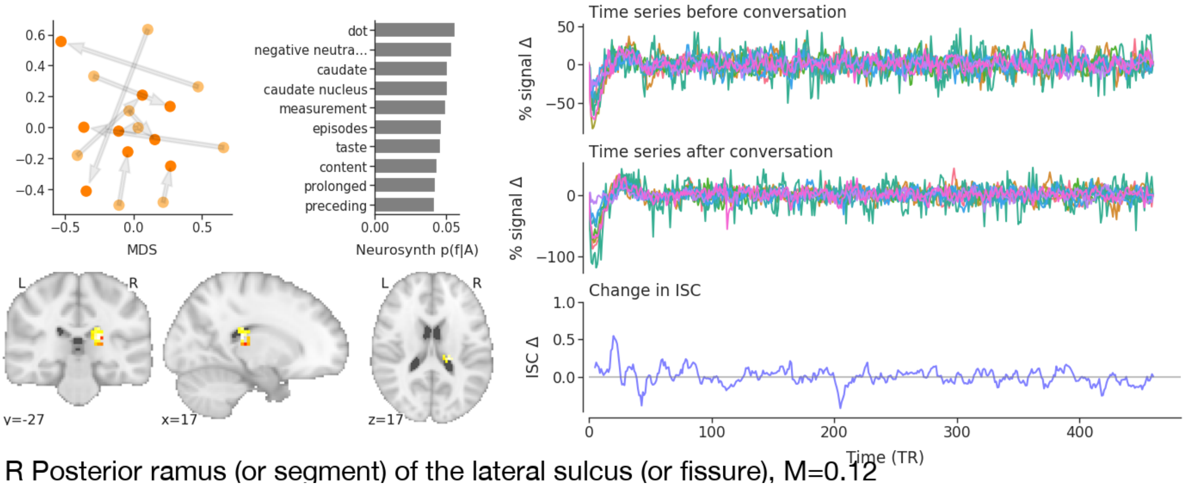


**Supplementary Figure 98:** Change in ISC. Movie clip: The Master. Group: Control

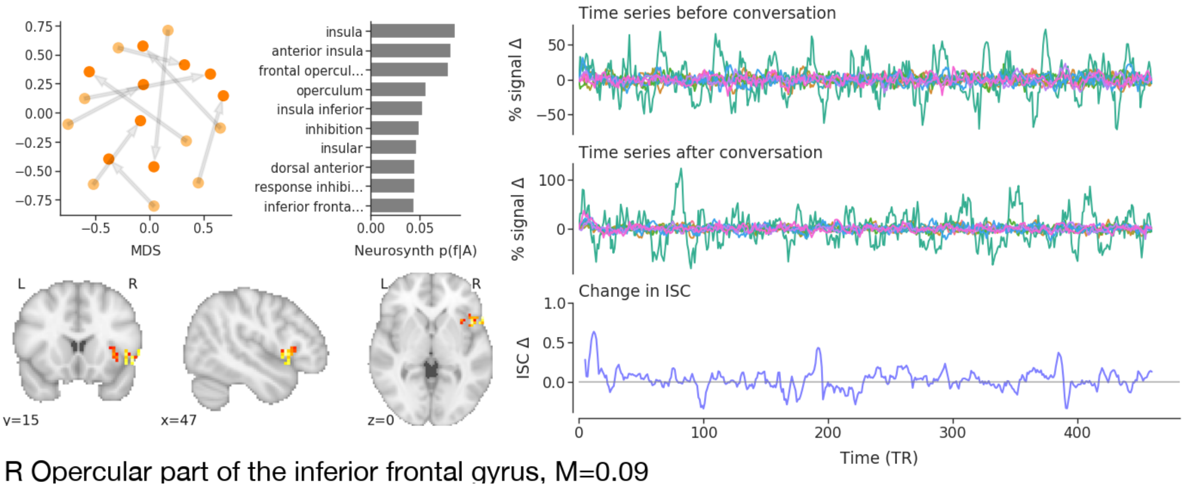


**Supplementary Figure 99:** Change in ISC. Movie clip: The Master. Group: Control

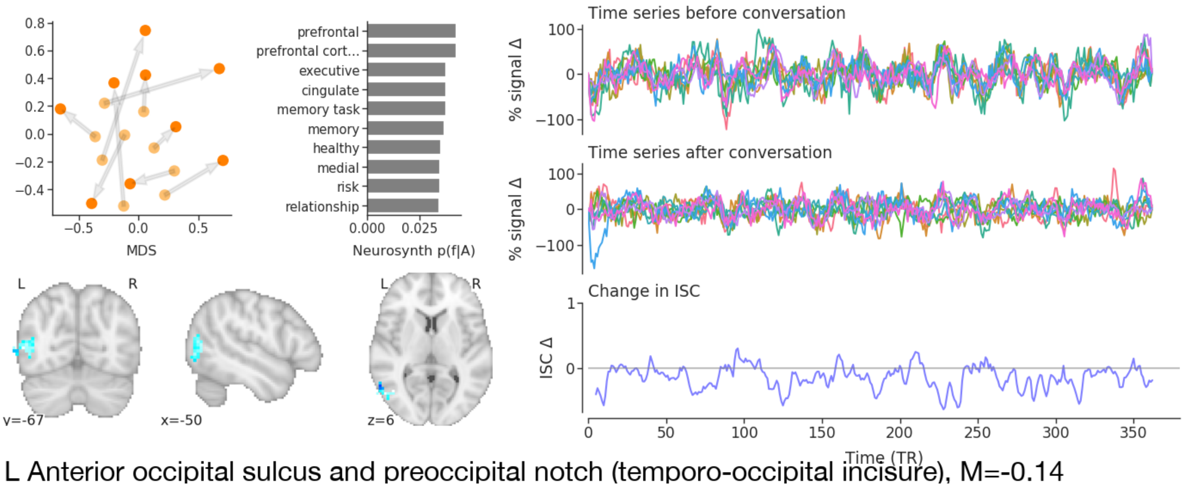


**Supplementary Figure 100:** Change in ISC. Movie clip: Y tu mamá también. Group: Control

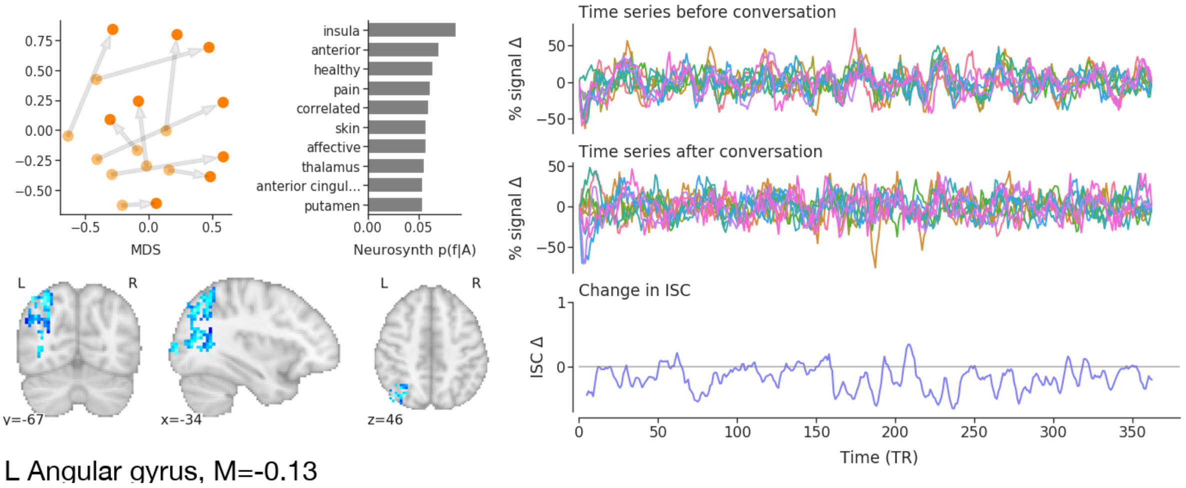


**Supplementary Figure 101:** Change in ISC. Movie clip: Y tu mamá también. Group: Control

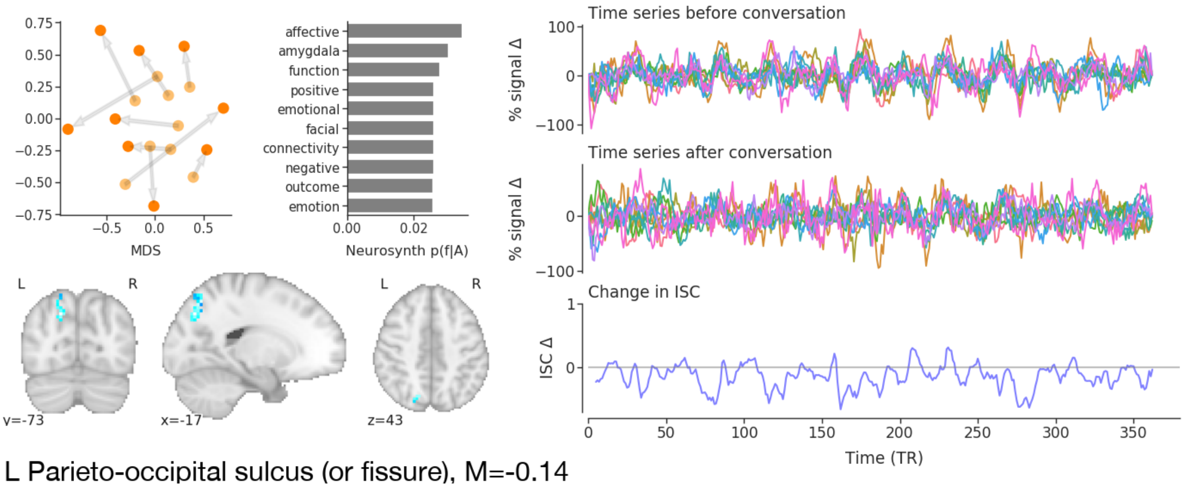


**Supplementary Figure 102:** Change in ISC. Movie clip: Y tu mamá también. Group: Control

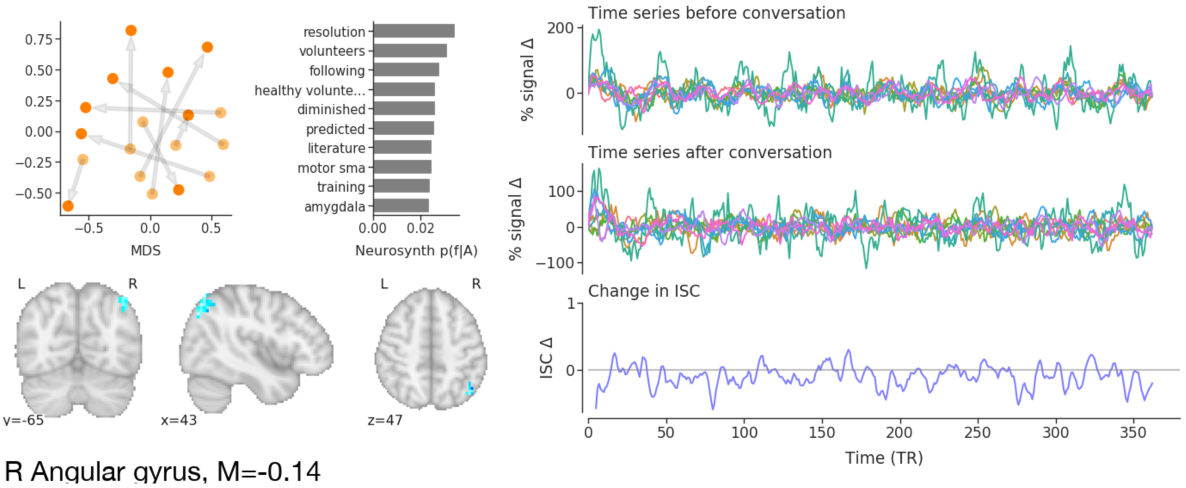


**Supplementary Figure 103:** Change in ISC. Movie clip: Y tu mamá también. Group: Control

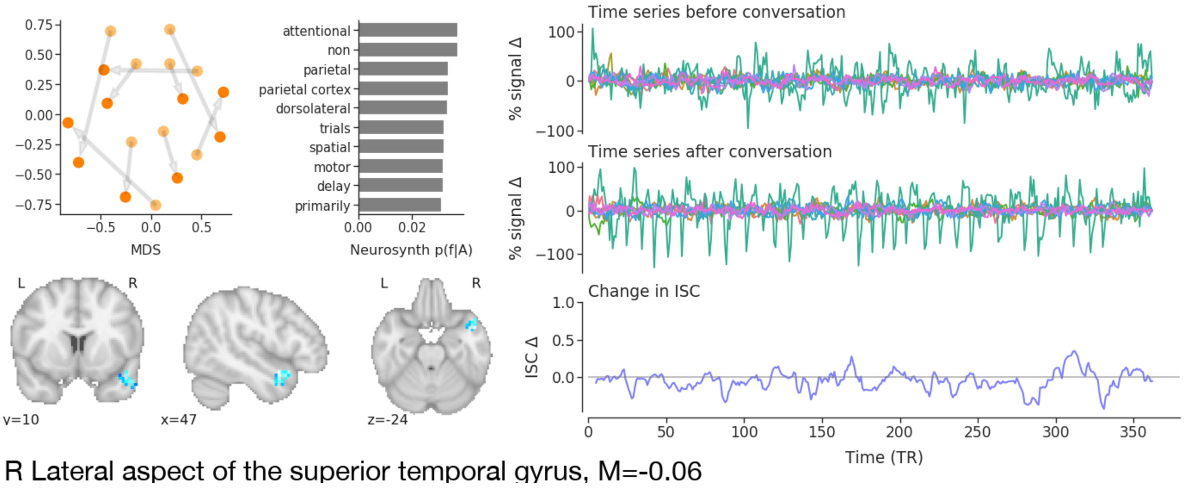


**Supplementary Figure 104:** Change in ISC. Movie clip: Y tu mamá también. Group: Control

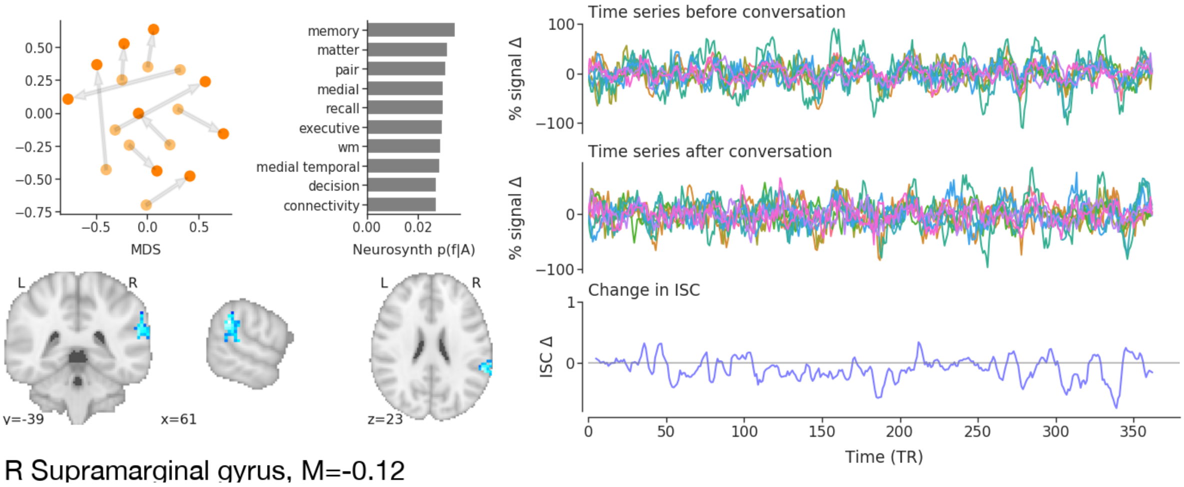


**Supplementary Figure 105:** Change in ISC. Movie clip: Y tu mamá también. Group: Control

## Supplementary references

1. Destrieux, C., Fischl, B., Dale, A. & Halgren, E. Automatic parcellation of human cortical gyri and sulci using standard anatomical nomenclature. *NeuroImage* **53**, 1–15 (2010).
