# Supplementary material for: Consensus-building conversation leads to neural alignment
Source: Nat Commun. 2024 May 10;15:3936. doi: 10.1038/s41467-023-43253-8 (PMC11087652; doi:10.1038/s41467-023-43253-8)
Supplement: Supplementary file 3 — Description of Additional Supplementary Files [file 41467_2023_43253_MOESM3_ESM.pdf]

Description of Additional Supplementary Files

File name: Supplementary Data 1
Description: Table of significant clusters across all fMRI analyses, corrected for multiple comparisons (cluster forming threshold: p=.01, minimum cluster size: 32 voxels, p<.05 corrected), with mean, peak, and 95% CI columns describing beta estimates for each significant cluster and atlas labels from Destrieux, Fischl, Dale, & Halgren (2010).

File name: Supplementary Data 2
Description: Regression table for word use analysis. Beta values are interpretable as predicted increases in the number of standard deviations from the network-wide mean PCA centrality and experiment-wide perceived status values.
