# Supplementary material for: Consensus-building conversation leads to neural alignment
Source: Nat Commun. 2024 May 10;15:3936. doi: 10.1038/s41467-023-43253-8 (PMC11087652; doi:10.1038/s41467-023-43253-8)
Supplement: Supplementary file 6 — Reporting Summary [file 41467_2023_43253_MOESM6_ESM.pdf]

nature portfolio

Corresponding author(s): Beau Sievers
Last updated by author(s): Sep 4, 2023

Reporting Summary

Nature Portfolio wishes to improve the reproducibility of the work that we publish. This form provides structure for consistency and transparency in reporting. For further information on Nature Portfolio policies, see our Editorial Policies and the Editorial Policy Checklist.

Statistics

For all statistical analyses, confirm that the following items are present in the figure legend, table legend, main text, or Methods section.

n/a	Confirmed	
☐	☒	The exact sample size (*n*) for each experimental group/condition, given as a discrete number and unit of measurement
☐	☒	A statement on whether measurements were taken from distinct samples or whether the same sample was measured repeatedly
☐	☒	The statistical test(s) used AND whether they are one- or two-sided<br>*Only common tests should be described solely by name; describe more complex techniques in the Methods section.*
☐	☒	A description of all covariates tested
☐	☒	A description of any assumptions or corrections, such as tests of normality and adjustment for multiple comparisons
☐	☒	A full description of the statistical parameters including central tendency (e.g. means) or other basic estimates (e.g. regression coefficient) AND variation (e.g. standard deviation) or associated estimates of uncertainty (e.g. confidence intervals)
☐	☒	For null hypothesis testing, the test statistic (e.g. F, t, r) with confidence intervals, effect sizes, degrees of freedom and P value noted<br>*Give P values as exact values whenever suitable.*
☒	☐	For Bayesian analysis, information on the choice of priors and Markov chain Monte Carlo settings
☐	☒	For hierarchical and complex designs, identification of the appropriate level for tests and full reporting of outcomes
☐	☒	Estimates of effect sizes (e.g. Cohen's *d*, Pearson's *r*), indicating how they were calculated

Our web collection on statistics for biologists contains articles on many of the points above.

Software and code

Policy information about availability of computer code

Data collection	fMRI data collected using a 3T Siemens Prisma scanner with a 32-channel head coil. fMRI data collection is described in Detailed Methods in the Supplementary Materials. Survey data were collected using SurveyMonkey.
Data analysis	All software used for data analysis is described in Detailed Methods in the Supplementary Materials. Software packages used included Python 2.7 and 3.10, NumPy 1.24.2, AFNI 21.2.00, ANTs 2.3.5, and FSL 6.0.5, and R 4.3.1. Custom code is available in a repository with a DOI, cited in the manuscript.

For manuscripts utilizing custom algorithms or software that are central to the research but not yet described in published literature, software must be made available to editors and reviewers. We strongly encourage code deposition in a community repository (e.g. GitHub). See the Nature Portfolio guidelines for submitting code & software for further information.

Data

Policy information about availability of data

All manuscripts must include a data availability statement. This statement should provide the following information, where applicable:

- Accession codes, unique identifiers, or web links for publicly available datasets
- A description of any restrictions on data availability
- For clinical datasets or third party data, please ensure that the statement adheres to our policy

The raw fMRI data generated in this study have been deposited in the NIMH Data Archive database under collection ID #771, available at http://

dx.doi.org/10.15154/1504150.101 Stimuli, anonymized conversation transcripts, conversation ratings, social network analysis derivatives, fMRI analysis derivatives, preprocessing code, analysis code, and figure rendering code are available in the Open Science Framework database, available at https://osf.io/kr9fb/.102 The raw social network data are protected and are not available due to data privacy laws.

Research involving human participants, their data, or biological material

Policy information about studies with human participants or human data. See also policy information about sex, gender (identity/presentation), and sexual orientation and race, ethnicity and racism.

Reporting on sex and gender	Findings do not apply to only one sex or gender. 49 participants participated in the fMRI study; 23 male, 26 female based on free-response self-report. We had no hypotheses concerning sex or gender, so sex and gender were not considered in the study design beyond recruiting a gender-balanced participant sample. Gender self-report data is available in the released dataset.
Reporting on race, ethnicity, or other socially relevant groupings	No race or ethnicity data were collected or used.
Population characteristics	See below.
Recruitment	Participants were recruited via an announcement made in the classroom, and volunteered to participate. Accordingly, we limit the scope of generalization.
Ethics oversight	Study was approved by the Dartmouth Committee for the Protection of Human Subjects

Note that full information on the approval of the study protocol must also be provided in the manuscript.

Field-specific reporting

Please select the one below that is the best fit for your research. If you are not sure, read the appropriate sections before making your selection.

☐ Life sciences ☒ Behavioural & social sciences ☐ Ecological, evolutionary & environmental sciences

For a reference copy of the document with all sections, see nature.com/documents/nr-reporting-summary-flat.pdf

Behavioural & social sciences study design

All studies must disclose on these points even when the disclosure is negative.

Study description	Quantitative experimental
Research sample	MBA students at a business school in the rural United States (n=49, 23 male, 26 female based on free-response self-report; age range 26–32, mean age=27.66). Detailed information in manuscript. Sample not representative of the general US population. Sample chosen because students provided information suitable for mapping their social networks as part of their coursework.
Sampling strategy	Convenience sample. Because the methods are novel, no sample size calculation was performed. Sample sizes were decided based on practical scheduling limitations. Accordingly, we explicitly limit our conclusions (see Discussion and Limitations on generalization in Supplementary Material).
Data collection	fMRI data collected on a 3T Siemens Prisma scanner with a 32-channel head coil. Survey data recorded using SurveyMonkey. Audio recordings of participants (used for transcriptions) were made using a consumer-grade webcam. The researcher was not hypothesis blind, but was in a separate control room during fMRI scanning and was not in the room for group conversations.
Timing	Data were collected from February, 2017 through May, 2019.
Data exclusions	A total of 59 participants underwent fMRI scanning, however, 10 participants were excluded, yielding 49 participants. 5 participants were excluded because of technical difficulties during scanning, 1 was excluded because the scanner compatible glasses were insufficient and they couldn't see actors' facial expressions, 2 were excluded because they terminated the scan session due to discomfort, 1 was excluded because they were absent from the group discussion, and 1 was excluded because an anatomical anomaly was detected (this participant was referred to a neurologist for follow-up in accordance with Dartmouth Brain Imaging Center safety policies). Due to a technical error, three groups were given an incorrect version of the survey for fMRI session 2 (after conversation) that did not include run-by-run agreement ratings or yes-or-no questions for the repeated movie clips. These same three groups were also not given pen-and-paper surveys after the group session, but instead verbally confirmed that they agreed with the group consensus (the realization that this was inadequate led to the introduction of the pen-and-paper survey).
Non-participation	See "data exclusions," above.
Randomization	Participants were allocated into groups randomly, subject to scheduling constraints.

Reporting for specific materials, systems and methods

We require information from authors about some types of materials, experimental systems and methods used in many studies. Here, indicate whether each material, system or method listed is relevant to your study. If you are not sure if a list item applies to your research, read the appropriate section before selecting a response.

Materials & experimental systems

n/a	Involved in the study
☒	☐ Antibodies
☒	☐ Eukaryotic cell lines
☒	☐ Palaeontology and archaeology
☒	☐ Animals and other organisms
☒	☐ Clinical data
☒	☐ Dual use research of concern
☒	☐ Plants

Methods

n/a	Involved in the study
☒	☐ ChIP-seq
☒	☐ Flow cytometry
☐	☒ MRI-based neuroimaging

Magnetic resonance imaging

Experimental design

Design type: Movie viewing

Design specifications: fMRI session 1: 7 movie viewing runs
fMRI session 2: 10 movie viewing runs

Behavioral performance measures: Survey on participants' understanding of the movie narrative

Acquisition

Imaging type(s): Functional, structural

Field strength: 3T

Sequence & imaging parameters: Participants were scanned at the Dartmouth Brain Imaging Center using a 3T Siemens Prisma scanner with a 32-channel head coil. A high resolution T1-weighted MPRAGE anatomical scan (2.32ms TE; 2300ms TR; .9x.938x.938mm resolution) was performed at the beginning of each scanning session. Functional images were acquired using an echo-planar sequence (32ms TE; 727ms TR; 53° flip angle; 3x3x3mm resolution). The number of scans per run varied depending on the stimulus presented. Sound was delivered using an in-ear headphone system. Foam padding was placed around participants' heads to minimize motion.

Area of acquisition: Whole brain

Diffusion MRI ☐ Used ☒ Not used

Preprocessing

Preprocessing software: ANTs: brain extraction; FSL: motion correction, tissue segmentation. AFNI: deobliquing, registration, smoothing (iteratively smoothed to 6mm FWHM)

Normalization: Nonlinear transformation using ANTs

Normalization template: MNI152 non-linear asymmetrical

Noise and artifact removal: Nuisance regressors included: 6 motion parameters, framewise displacement outliers (one binary regressor per outlier), tissue confounds including the average time course of high SD voxels outside the grey matter mask, the average BOLD signal in cerebrospinal fluid, and the average white matter signal, linear and quadratic trends, and an intercept term.

Volume censoring: EPI images were motion corrected using FSL mcflirt and motion outliers (framewise displacement > .9) were detected using FSL fsl_motion_outliers.

Statistical modeling & inference

Model type and settings: Mass univariate intersubject correlation model, calculated using AFNI 3dTcorrelate, with additional testing using NumPy

Effect(s) tested: Changes in intersubject correlation after conversation, corresponding to several kinds of social influence

Specify type of analysis: ☒ Whole brain ☐ ROI-based ☐ Both

Statistic type for inference (See Eklund et al. 2016)	Cluster-wise
Correction	Multiple comparisons correction was performed at the cluster level using AFNI 3dClustSim. Cluster simulation used a non-Gaussian ACF model allowing for heavy tails, using model parameters estimated from the mean of the collected fMRI data using AFNI 3dFWHMx.

Models & analysis

n/a	Involved in the study
☒	☐ Functional and/or effective connectivity
☒	☐ Graph analysis
☒	☐ Multivariate modeling or predictive analysis
